# A database-driven approach identifies additional diterpene synthase activities in the mint family (Lamiaceae)

**DOI:** 10.1074/jbc.RA118.006025

**Published:** 2018-11-29

**Authors:** Sean R. Johnson, Wajid Waheed Bhat, Jacob Bibik, Aiko Turmo, Britta Hamberger, Evolutionary Mint Genomics Consortium, Björn Hamberger

**Affiliations:** From the Departments of ‡ Biochemistry and Molecular Biology and; § Pharmacology and Toxicology,; ¶ Michigan State University, East Lansing, Michigan 48824; Purdue University; Michigan State University; Michigan State University; Purdue University; Florida Museum of Natural History; Michigan State University, Florida Museum of Natural History; Purdue University; The John Innes Centre; Florida Museum of Natural History; Purdue University; The John Innes Centre; Florida Museum of Natural History; Michigan State University; John Innes Centre; John Innes Centre; Florida Museum of Natural History, University of Florida; Florida Museum of Natural History; Michigan State University; Michigan State University; Michigan State University

**Keywords:** secondary metabolism, terpenoid, plant biochemistry, transcriptomics, biosynthesis, chemotaxonomy, diterpene synthase, enzymes, Lamiaceae, phytochemistry

## Abstract

Members of the mint family (Lamiaceae) accumulate a wide variety of industrially and medicinally relevant diterpenes. We recently sequenced leaf transcriptomes from 48 phylogenetically diverse Lamiaceae species. Here, we summarize the available chemotaxonomic and enzyme activity data for diterpene synthases (diTPSs) in the Lamiaceae and leverage the new transcriptomes to explore the diTPS sequence and functional space. Candidate genes were selected with an intent to evenly sample the sequence homology space and to focus on species in which diTPS transcripts were found, yet from which no diterpene structures have been previously reported. We functionally characterized nine class II diTPSs and 10 class I diTPSs from 11 distinct plant species and found five class II activities, including two novel activities, as well as a spectrum of class I activities. Among the class II diTPSs, we identified a *neo*-cleroda-4(18),13*E*-dienyl diphosphate synthase from *Ajuga reptans*, catalyzing the likely first step in the biosynthesis of a variety of insect-antifeedant compounds. Among the class I diTPSs was a palustradiene synthase from *Origanum majorana*, leading to the discovery of specialized diterpenes in that species. Our results provide insights into the diversification of diterpene biosynthesis in the mint family and establish a comprehensive foundation for continued investigation of diterpene biosynthesis in the Lamiaceae.

## Introduction

Diterpenoid specialized metabolites are widespread among plants but are particularly diverse and abundant in the Lamiaceae (mint) family. According to the Dictionary of Natural Products (DNP)^4^ (version 26.2) (1), more than 13,000 distinct diterpenes have been reported from plants, about 3,000 of those in at least one species from the Lamiaceae. In the Lamiaceae, the majority of diterpenes share a decalin core, characteristic of labdane-related diterpenes. In angiosperms, biosynthesis of labdane-related diterpenes starts with the action of a class II diterpene synthase (diTPS) from the TPS-c subfamily (2, 3), which catalyzes the conversion of the central precursor, geranylgeranyl diphosphate (GGPP), into a bicyclic prenyl diphosphate intermediate (*e.g.* copalyl diphosphate (CPP)). A class I diTPS from the TPS-e subfamily then acts to remove the diphosphate moiety and form additional rings, double bonds, or hydroxyl groups. TPS-c and TPS-e enzymes are ubiquitous in angiosperms, catalyzing the first two steps in the gibberellin phytohormone biosynthesis pathway, the conversion of GGPP to *ent*-CPP and of *ent*-CPP to *ent*-kaurene, respectively. Labdane-related diterpene-specialized metabolites may arise from alternative decoration of *ent*-kaurene after the diTPS-catalyzed reactions, from gene duplication and neo-functionalization of *ent*-CPP and *ent*-kaurene synthases (4), or from further functional diversification of diTPSs already involved in specialized metabolism. Most class I diTPSs can act on multiple class II products, leading to an increase in the total number of distinct products that can be formed through different combinations of class II and class I enzymes (5). Together, the diTPSs give shape to the diterpene skeleton, which can then undergo further modification by cytochromes P450, acyl transferases, or other enzymes (6–10).

Previous investigations into diterpenoid biosynthetic pathways in the Lamiaceae have focused on medicinal diterpenes, such as the cAMP booster forskolin, from *Plectranthus barbatus* (syn. *Coleus forskohlii*); the tanshinones, from *Salvia miltiorrhiza*, which have many uses in Chinese traditional medicine; the dopaminergic vitexilactone from *Vitex agnus-castus*; the potential anti-diabetic and vasorelaxant marrubiin, from *Marrubium vulgare*; and the potent hallucinogen salvinorin A from *Salvia divinorum* (8, 11–17). Other research was motivated by the industrial value of diterpenes such as sclareol, from *Salvia sclarea*, which can be used in the semisynthesis of the commodity chemical Ambrox and antioxidant carnosic acid (18–20).

Recently, we made available leaf transcriptomes of 48 species from the Lamiaceae (21). In the present work, we performed a detailed analysis of the available chemotaxonomic and enzyme function data from the Lamiaceae, showing hundreds of diterpene skeletons that could not be accounted for by known enzymes. We therefore saw an opportunity to mine for diTPSs with previously unknown activities. Using homology searches with known diTPSs from mints (Data set S1), we identified a total of 163 candidate diTPSs from the new transcriptomes (Data set S2). By combining and cross-referencing the transcriptome data, chemotaxonomic data, and earlier enzyme data, we narrowed down the list of candidates to select genes with minimal homology to known enzymes, genes from species where the reported diterpenes could not be explained by known activities, and genes from species where no diterpenes have been reported, but where the transcriptome data show an enlargement of the TPS-c or TPS-e gene families.

We report nine class II diTPSs accounting for five diphosphate intermediates and 10 class I diTPSs accounting for a wide variety of additional products. Some of the new enzymes give access to intermediates that were previously difficult or impossible to produce biosynthetically. Specifically, we identified *Ajuga reptans* ArTPS2 as producing *neo*-cleroda-4(18),13*E*-dienyl diphosphate, the likely precursor to a wide variety of bioactive diterpenoids, including antifeedants against insects (22–24). Another class II diTPS, *Pogostemon cablin* PcTPS1, was found to catalyze the formation of (10*R*)-labda-8,13*E*-dienyl diphosphate, a likely precursor to an entire suite of diterpenes from the Lamioideae clade within the Lamiaceae. Further, in *Origanum majorana*, a culinary herb without reported diterpene accumulation, the characterization of multiple new diTPSs led us to the discovery of some of the corresponding diterpenes *in planta*. We anticipate that this work will serve as a new foundation for continued discovery of diTPSs in the Lamiaceae. To this end, we have endeavored to make our analyses and results, including gene sequences, raw and processed spectroscopic data, code, and extensive data tables, available in human- and machine-readable formats to be used, adapted, and extended by ourselves and other researchers in the future.

## Results

### Estimating the diversity of diterpenoids in the Lamiaceae

To help determine the most promising species to find previously unknown diTPS activities, it was necessary to compile a data set of diterpene occurrence in Lamiaceae species and a data set of functionally characterized diTPS genes from the Lamiaceae. Information about diterpene occurrence was collected from three sources: SISTEMAT, DNP, and NAPRALERT. SISTEMAT (25) contains Lamiaceae diterpenes reported up to 1997, including 91 unique carbon skeletons (the core alkanes, disregarding all desaturation, acyl-side chains, heteroatoms, and stereochemistry) from 295 species and 51 genera. We were unable to obtain an electronic copy of SISTEMAT, so we reconstructed it based on the figures and tables in the paper.

The DNP (1) includes a wealth of information on diterpenes from the Lamiaceae, including full structures and the species where those structures have been reported. NAPRALERT (26) identifies compounds by their common name rather than their structure or skeleton, associates the compound names to genus and species names, and gives various other information, such as the tissue where the compound was found.

To enable comparison among the databases and cross-referencing with transcriptome and enzyme data, all genus and species names were converted into TaxIDs from the NCBI Taxonomy database (27). To put structure occurrence into clearer evolutionary context, we annotated each genus as a member of one of the 12 primary, monophyletic clades that form the backbone of Lamiaceae, as delineated by Li *et al.* (28) on the basis of chloroplast genome sequence. In the context of diTPSs, examination of skeletons can be helpful because the skeleton often resembles the diTPS product more obviously than a highly decorated downstream product would. We therefore extracted the skeletons from the DNP structures (Fig. S1 shows a graphical example of skeleton extraction). We assigned each skeleton according its reported presence in the 12 clades, as well as to genera, to help distinguish skeletons that may have arisen early in Lamiaceae evolution *versus* those that arose more recently. A full tabulation of the skeletons from SISTEMAT and DNP can be found as Data set S3.

**Figure 1.**
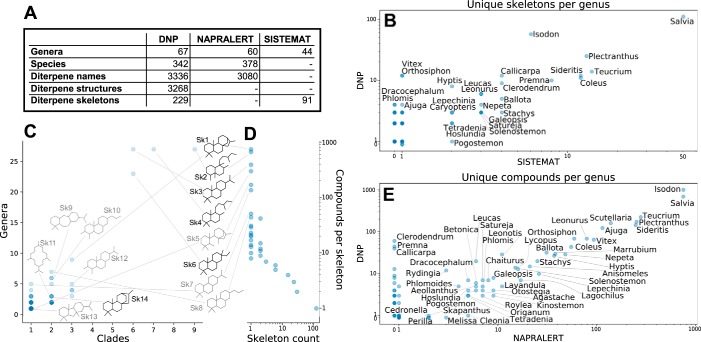
**Distribution of diterpenes in the Lamiaceae.** *A*, comparison of different sources for data about Lamiaceae diterpene chemotaxonomy. *B*, diterpene skeletons per genus according to both the DNP and SISTEMAT. *C*, distribution of skeletons among the genera and 12 primary clades of Lamiaceae, based on the DNP. A *circle* in *C* represents one skeleton, with its *vertical position* indicating how many genera that skeleton has been reported in and its *horizontal position* indicating how many of the 12 clades are represented by those genera. Structures are shown for selected skeletons; in *black* are those where a biosynthetic route is known from the Lamiaceae, and in *gray* are those for which the pathway remains unknown. *D*, distribution of compounds among skeletons, based on the DNP. Each *circle* in *D* represents a number of skeletons indicated by the *horizontal position* of the *circle*, with *vertical position* indicating the number of compounds reported with each of those skeletons; the data point in the *bottom right* shows that there are more than 100 skeletons that are represented by only one compound apiece. *E*, diterpene structures per genus according to both the DNP and NAPRALERT. Data points in *B–E* are represented by *semitransparent circles*, so *darker spots* indicate overlapping data points. Some genus name labels in *B* and *E* have been omitted due to space constraints. An exhaustive list of the occurrence of skeletons in genera of Lamiaceae is given in Data set S3, skeleton distribution.

The three databases were relatively consistent in their estimations of the diversity and distribution of diterpenes and diterpene skeletons (Fig. 1, *A*, *B*, and *E*). The data are snapshots of reports in the literature and certainly do not comprehensively reflect the true chemical diversity existing in nature. Some genera, such as *Salvia*, may be overrepresented due to their large number of species or their use in traditional medicine.

A total of 239 skeletons are represented, with five (the kaurane (Sk1), clerodane (Sk2), abietane (Sk3), labdane (Sk4), and pimarane (Sk6)) being, by far, the most widely distributed and accounting for most of the total structures (Fig. 1, *C* and *D*). The clerodane skeleton, for example, has the widest distribution, having been reported in 27 genera representing 9 of the 12 primary clades, absent only in *Tectona* and two clades from which no diterpenes have yet been reported. The large number of less common, taxonomically restricted skeletons, including over 100 skeletons with only one associated compound (Fig. 1*D*), suggests that new diterpene synthase activities are continuously and independently arising across the Lamiaceae family tree. Therefore, a search across many species and genera should be a good strategy for finding diterpene synthases with new activities.

### Identifying candidate diterpene synthase genes

Through a comprehensive literature search, we built a reference set of known Lamiaceae diTPSs and their activities. 54 functional diTPSs have been reported in this family, corresponding to 30 class II and 24 class I enzymes (Data set S1). Combinations of these diterpene synthases account for 27 distinct products accounting for six different skeletons, the five widely distributed skeletons (Sk1–4 and Sk6) as well as the less common atisane (Sk14) skeleton. This leaves 233 skeletons for which the biosynthetic route remains unknown. It is important to note that a single skeleton can correspond to multiple diTPS products, distinguished through stereochemical configuration, position of double bonds, or functionalization, so there is also a possibility of finding new diTPS activities for skeletons already accounted for by known enzymes.

**Figure 2.**
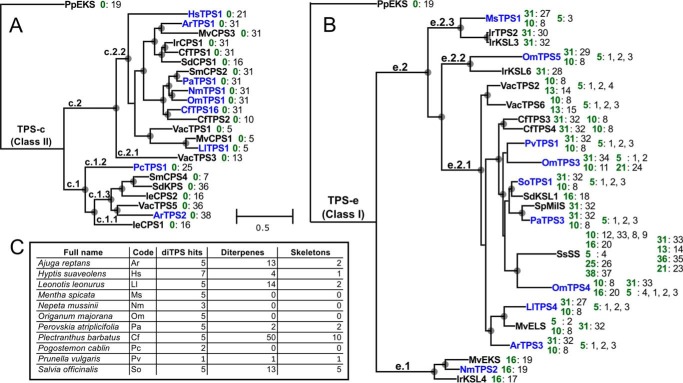
**Maximum likelihood trees of newly characterized (*blue*) class II (*A*), and class I (*B*) diTPS enzymes in the context of selected previously reported (*black*) diTPSs from the Lamiaceae.** The bifunctional *ent*-kaurene synthase from *P. patens* is used as an outgroup. After each enzyme are listed the experimentally verified substrates (*green*) and their products, *numbers* corresponding to *compound numbers* in Fig. 3. The *scale bar* applies to both trees, and units are substitutions per site. *Circles* at branch points indicate bootstrap support of at least 75%. *C*, all of the species we chose to clone diTPSs from, their total number of diTPS candidate sequences, and the number of unique diterpene structures and skeletons for those species, based on the DNP. Abbreviations for species not listed in *C* are as follows. *Ie*, *Isodon eriocalyx*; *Ir*, *Isodon rubescens*; *Mv*, *Marrubium vulgare*; *Sd*, *Salvia divinorum*; *Sm*, *Salvia miltiorrhiza*; *Sp*, *Salvia pomifera*; *Ss*, *Salvia sclarea*; *Vac*, *Vitex agnus-castus*.

We used BLAST searches (29) with the list of known Lamiaceae diTPSs as query sequences to mine the 48 new Lamiaceae leaf transcriptomes (21) for candidate diTPSs. A total of 163 candidate sequences met our search criteria. The count of diTPS candidates was cross-referenced to the count of diterpenes and diterpene skeletons reported from each species and genus (Fig. 2*C* and Data set S2). Finally, a phylogenetic tree was generated from the peptide sequences from the reference set, alongside those from the new transcriptome data, including established substrates and products for each enzyme (Fig. 2 (*A* and *B*) and Fig. S20). We selected candidate genes from species, such as *Mentha spicata* and *O. majorana*, where the transcriptome data showed multiple candidate diTPSs but where few or no diterpene structures have been reported. We also selected genes that had relatively low homology to known enzymes, as judged by visual inspection of the phylogenetic trees, and in this way attempted to evenly cover the sequence homology space. Finally, we chose a few candidates from *P. barbatus* and *Salva officinalis*, based on the great diversity of diterpenes that have been reported from their genera.

### Characterization of class II diTPSs

**Figure 3.**
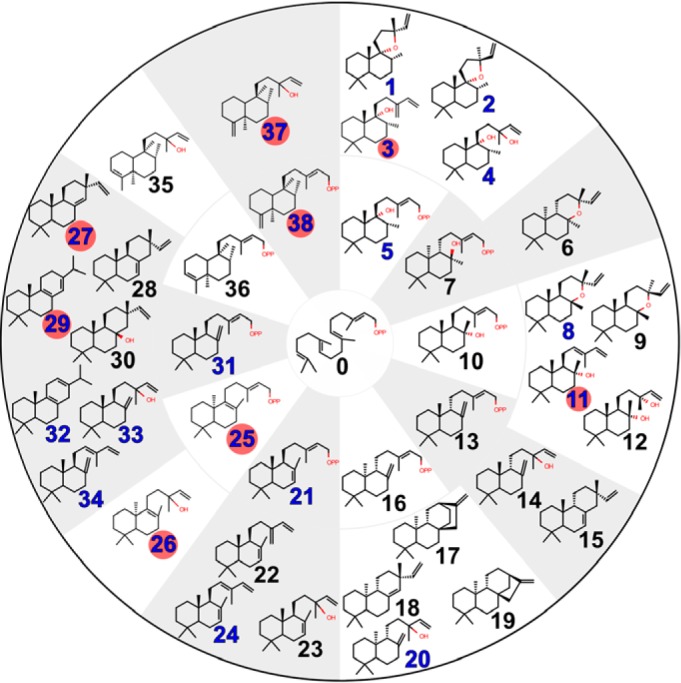
**Reported products of diterpene synthases from the Lamiaceae.** *Blue numbers*, compounds experimentally verified to be products of new enzymes from this study. *Red circle*, compounds that previously were inaccessible biosynthetically or were accessible only as minor components of multiproduct enzymes but are the single product of a newly characterized enzyme. At the center is GGPP, a precursor to all of these diterpenes. *Inner ring*, class II products; *outer ring*, class I products derived from the compound in the connected segment of the inner ring.

Fig. 3 presents a summary of Lamiaceae diTPS activities reported from previous work, together with our newly characterized diTPS activities. Class II activities were established based on comparisons of extracts from *Nicotiana benthamiana* transiently expressing new genes with those expressing known diTPS combinations. Class II diTPS products retain the diphosphate group from the GGPP substrate. When expressed *in vivo*, whether in *Escherichia coli* or *N. benthamiana*, without a compatible class I diTPS, a diphosphate product degrades to the corresponding alcohol, presumably by the action of nonspecific endogenous phosphatases. Due to difficulties in purifying and structurally characterizing diphosphate class II products, it is customary in the field to instead characterize these derivative alcohols (14, 17), which is the approach we have taken. For clarity, we indicate the alcohol by appending an “**a**” to the compound number; for example, **16a** refers to *ent*-copalol.

ArTPS1, PaTPS1, NmTPS1, OmTPS1, and CfTPS16 were identified as (+)-CPP (**31**) synthases by comparison with products of *P. barbatus* (syn. *C. forskohlii*) CfTPS1, and the reference combination of CfTPS1 combined with CfTPS3, yielding miltiradiene (30) (Fig. S7). LlTPS1 was identified as a peregrinol diphosphate (PgPP) (**5**) synthase based on a comparison of products with *M. vulgare* MvCPS1 (15), and MvCPS1 combined with *M. vulgare* 9,13-epoxylabdene synthase (MvELS) (Fig. S5*B*) and *S. sclarea* sclareol synthase (SsSS) (Fig. S5*D*) (31).

**Figure 4.**
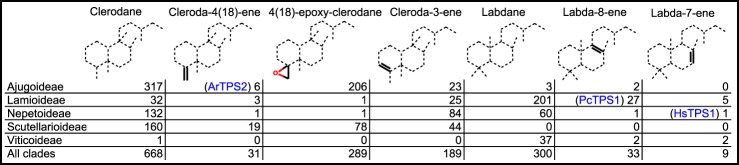
**Distribution among selected Lamiaceae clades of diterpenes with various structural patterns.** *Blue enzyme names* are placed according to the pattern they install and the clade of the species they were cloned from. A *solid line* indicates that only compounds with the bond type shown at that position are counted. A *dashed line* indicates that all types of bonds and substituents are counted at that position. Based on data from the DNP (1).

HsTPS1 was identified as a (5*S*,9*S*,10*S*)-labda-7,13*E*-dienyl diphosphate (**21**) synthase based on comparison with the product of an enzyme from *Grindelia robusta*, GrTPS2 (32) (Fig. S8*B*) and by NMR of the alcohol derivative (**21a**) (Fig. S11). Normal absolute stereochemistry was assigned to the HsTPS1 product based on the optical rotation of **21a**, [α]_D_ +8.3° (c. 0.0007, CHCl_3_) (c.f. lit. [α]_D_ +5°, c. 1.0, CHCl_3_ (33); [α]_D_^25^ +12°, c. 0.69, CHCl_3_ (34)). When HsTPS1 was expressed in *N. benthamiana*, we also noticed the formation of labda-7,13(16),14-triene (**22**) (Fig. S12), which seemed to be enhanced by co-expression with CfTPS3 (Fig. S8*B*). The combination of HsTPS1 with OmTPS3 produced labda-7,12*E*,14-triene (**24**) (Fig. S13) (35), both in *N. benthamiana* and *in vitro* (Figs. S8*B* and S9*A*), which has previously been accessible only by combinations of bacterial enzymes (36). Labdanes with a double bond at the 7-position have not been reported in *Hyptis suaveolens* and do not seem to be common in the Lamiaceae. Of nine compounds with the labdane skeleton and a double bond at 7 (Fig. 4), only one was from the same clade as *H. suaveolens*. (13*E*)-ent-labda-7,13-dien-15-oic acid, from *Isodon scoparius* (37), has the opposite absolute stereochemistry to the HsTPS1 product, likely not deriving from a paralog of HsTPS1 because the absolute stereochemistry of labdane skeletons is not known to change after the diTPS steps.

**Figure 5.**
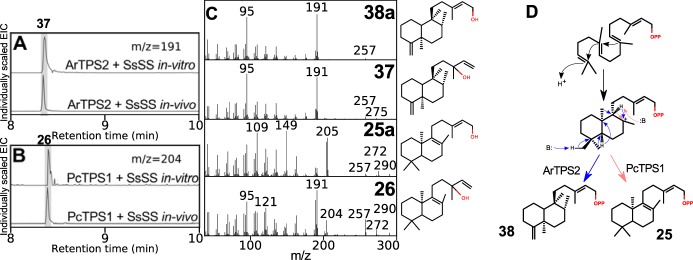
**Activities of ArTPS2 and PcTPS1.** *A* and *B*, GC-MS–extracted ion chromatograms of activity assays for ArTPS2 + SsSS (*A*) and PcTPS1 + SsSS (*B*), *in vitro* with purified protein fed with GGPP and *in vivo* from *N. benthamiana* transiently expressing the gene combinations. *C*, mass spectra for the products of ArTPS2 and PcTPS1, and their combinations with SsSS. *D*, proposed mechanisms for ArTPS2 (*blue*) and PcTPS1 (*pink*).

ArTPS2 was identified as a (5*R*,8*R*,9*S*,10*R*)-*neo*-cleroda-4(18),13*E*-dienyl diphosphate (**38**) synthase. The combination of ArTPS2 and SsSS generated *neo*-cleroda-4(18),14-dien-13-ol (**37**) (Fig. 5*A*). The structures of **37** and **38a** were determined by NMR (Figs. S17 and S18), including the comparison of **37** with chelodane (38), which, based on small differences in ^13^C shifts, may be a stereoisomer at the 13 position, and the comparison of **38a** with NMR of its enantiomer (39). Carbon 20 to 19, and 20 to 17 NOE interactions in **37** and **38a** (Figs. S17*G* and S18*F*) closely resembled those reported for (−)-kolavelol (**36a**) (17), suggesting (5*R*,8*R*,9*S*,10*R*) relative stereochemistry. The “*neo*” absolute configuration was established through optical rotation of **38a**, [α]_D_ +30° (c. 0.0025, CHCl_3_) (c.f. lit. [α]_D_ +20.9°, c. 0.7, CHCl_3_) (40). Previously reported clerodane diTPSs from the Lamiaceae produce kolavenyl diphosphate (**36**) (14, 16, 17), which has a double bond at the 3-position. Clerodanes with desaturation at 3 are spread throughout multiple clades but are most common in Nepetoideae (Fig. 4), which includes *S. divinorum*, one source of a kolavenyl diphosphate synthase. A plausible cyclization mechanism for ArTPS2 can readily be proposed (Fig. 5*D*). Clerodanes with a double bond at the 4(18) position are rare by comparison, but those with a 4(18)-epoxy moiety make up nearly half of the clerodanes reported in the Lamiaceae, including two-thirds of those reported from the Ajugoideae clade (Fig. 4). One such clerodane is clerodin (41), from which the clerodane skeleton gets its name. *Neo*-cleroda-4(18),13*E*-dienyl diphosphate is a logical biosynthetic precursor for the 4(18)-epoxy clerodanes, as we are not aware of any diTPSs that directly produce an epoxide moiety.

PcTPS1 was identified as a (10*R*)-labda-8,13E-dienyl diphosphate (**25**) synthase. The structure was established by comparison of ^13^C NMR of **25a** with previously reported spectra (34) (Fig. S14). The 10*R* (*ent*-) absolute stereochemistry was established by optical rotation of **25a** [α]_D_ −64° (c. 0.0008, CHCl_3_), (c.f. lit. [α]_D_^25^ −71.2°, c. 1.11, CHCl_3_) (42). The combination of PcTPS1 and SsSS, both *in vitro*, and in *N. benthamiana* expression produced (10*R*)-labda-8,14-en-13-ol (**26**) (Fig. 5*B*). The structure was determined by comparison of ^13^C NMR with a published spectrum (43) (Fig. S15*G*). We can propose a plausible mechanism for PcTPS1 activity (Fig. 5*D*). The double bond between positions 8 and 9 is present in 33 distinct compounds isolated from the Lamiaceae (Fig. 4). Most occur in the Lamioideae clade, which includes *P. cablin*, the source of PcTPS1. Absolute stereochemistries of the reported compounds are mixed, with some in the normal configuration (44) and others in the *ent-*configuration (45). As normal configuration 9-hydroxy labdanes are also abundant in Lamioideae, it is possible that the normal configuration 8(9) desaturated labdanes arise from dehydratase activities downstream of a PgPP synthase (MvCPS1 and its paralogs), whereas those in the *ent-*configuration arise from paralogs of PcTPS1. Another possibility is that some of the 8(9) desaturated labdanes reported as having normal absolute stereochemistry are actually *ent*-labdanes that were misassigned, as has occurred in at least one documented case (45).

### Characterization of class I diTPSs

**Figure 6.**
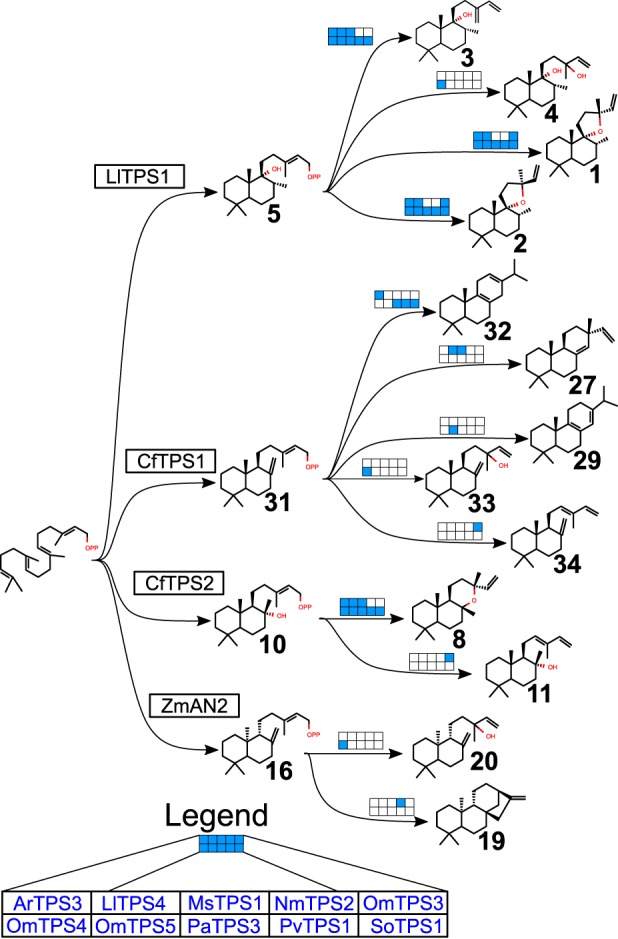
**Activities of new class I diTPSs.** *Filled-in blue boxes* indicate which enzymes are capable of each conversion. A diagram including new class II enzymes is available as Fig. S2.

Class I diTPS candidates were characterized by transient expression in *N. benthamiana* in combination with four class II enzymes: CfTPS1, a (+)-CPP (**31**) synthase; CfTPS2, a labda-13-en-8-ol diphosphate ((+)-8-LPP) (**10**) synthase (30); LlTPS1, a PgPP (**5**) synthase; or *Zea mays* ZmAN2, an *ent*-CPP (**16**) synthase (46) (GenBank^TM^ accession number AY562491). Substrates accepted by each enzyme and the products are indicated in Figs. 2*B* and 6, and GC-MS chromatograms of all combinations tested can be found as Figs. S3–S6.

NmTPS2 was identified as an *ent*-kaurene (**19**) synthase, converting *ent*-CPP into *ent*-kaurene (identified using *Physcomitrella patens* extract as a standard (47)) but not showing activity with any other substrate. The only other enzyme to show activity with *ent*-CPP was OmTPS4, which produced *ent*-manool (**20**), just as SsSS produces from *ent*-CPP (31).

PaTPS3, PvTPS1, SoTPS1, ArTPS3, OmTPS4, LlTPS4, OmTPS5, and MsTPS1 converted (+)-8-LPP to 13*R*-(+)-manoyl oxide (**8**), verified by comparison with the product of CfTPS2 and CfTPS3 (30). OmTPS3 produced *trans*-abienol (**11**), both *in vitro* and in *N. benthamiana* (Figs. S4*E* and S9*D*). The *trans*-abienol structure was determined by NMR (Fig. S10), with the configuration of the 12(13) double bond supported by comparison of the NOESY spectrum with that of a commercial standard for *cis*-abienol (Toronto Research Chemicals, Toronto, Canada). The product from CfTPS2 with OmTPS3 showed clear NOE correlations between positions 16 and 11 (Fig. S10*F*), whereas the *cis*-abienol standard showed correlations between 14 and 11 (Fig. S19). *Trans*-abienol is an alternative precursor to sclareol for semisynthesis of ambroxides, valuable amber odorants in the fragrance industry.

PaTPS3, PvTPS1, SoTPS1, and ArTPS3, LlTPS4, and OmTPS5 converted PgPP to a combination of **1**, **2**, and **3**, with some variation in the ratios between the products. Because perigrinol (**5a**) spontaneously degrades into **1**, **2**, and **3** under GC conditions (15), it was difficult to distinguish whether these enzymes have low activity, but specific products, or moderate activity with a mix of products. Nevertheless, differences in relative amounts of the products observed between LlTPS1 alone and in combination with these class I enzymes suggest that they do have some activity on PgPP. OmTPS4 produced **1**, **2**, **3**, and **4**. MsTPS1 produced only **3**, and OmTPS3 produced only **1** and **2**. PgPP products were established by comparison with MvCPS1, MvCPS1 with MvELS (15), and MvCPS1 with SsSS (31).

PaTPS3, PvTPS1, SoTPS1, and ArTPS3 converted (+)-CPP to miltiradiene (**32**), similarly to CfTPS3. OmTPS4 produced manool (**33**), as compared with SsSS. LlTPS4 and MsTPS1 produced sandaracopimaradiene (**27**), by comparison with a product from *Euphorbia peplus* EpTPS8 (48). OmTPS5 produced palustradiene (**29**), both *in vitro* and in *N. benthamiana*, as compared with a minor product from *Abies grandis* abietadiene synthase (GenBank^TM^ accession number U50768) (49) (Figs. S3*A* and S9*E*). Finally, OmTPS3 produced *trans*-biformene (**34**) *in vitro* and in *N. benthamiana* (Figs. S3*A* and S9*C*), as established by comparison of ^13^C NMR with the previously reported reference compound (50) (Fig. S16*G*), with *trans*-configuration further supported by clear NOE correlations between carbon 16 and 11 and the absence of NOE correlations between 14 and 11 (Fig. S16*F*).

### O. majorana accumulates palustradiene and other diterpenoids

**Figure 7.**
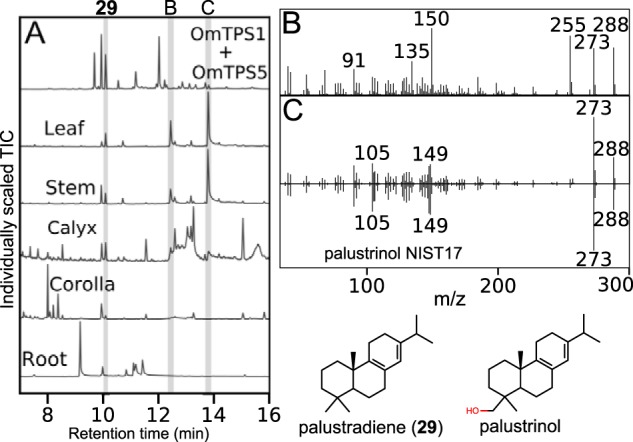
**Detection of diterpenoids in *O. majorana* tissues.** *A*, GC-MS total ion chromatograms of extract from *N. benthamiana* expressing OmTPS1 and OmTPS5, compared with extracts from various tissues of *O. majorana. B*, the mass spectrum of peak B, from *O. majorana* leaf. *C*, the mass spectrum of peak C from *O. majorana* leaf compared with reference spectrum for palustrinol from the NIST17 library.

The class I enzymes from *O. majorana*, OmTPS3, OmTPS4, and OmTPS5, all produced different products from (+)-CPP, which itself is the product of OmTPS1, from the same species. Despite the apparent richness of diterpene synthase activities of enzymes from *O. majorana*, we did not find any reports of diterpenes from that species either in our database searches (Fig. 2*C*) or in a subsequent literature search. To determine whether diterpene synthases are active in *O. majorana*, we looked for the products of the enzyme combinations with extracts from leaf, stem, calyx, corolla, and root. We detected palustradiene (**29**), the product of OmTPS1 and OmTPS5, in all tissues except roots (Fig. 7). In addition, we detected two diterpene alcohols in the stem, leaf, and calyx. One diterpene alcohol we could not identify, but the other was a close match to a reference spectrum for palustrinol, the 19-hydroxy derivative of palustradiene.

## Discussion

### Diversification of diterpene synthases in the Lamiaceae

Due to an increase in resolution at the taxonomic level and consistent clustering of enzymes with identical or related function, we propose a hierarchical scheme for classifying TPS genes in the Lamiaceae from the TPS-e and TPS-c subfamilies. TPS-c genes (class II diTPSs) from the Lamiaceae fall broadly into two clades (Fig. 2*A* and Fig. S20), which we have called c.1 and c.2, further divided into three and two subclades, respectively. The characterized genes from c.1.1 are all *ent-*CPP (**16**) synthases, presumably involved in gibberellin biosynthesis. The taxonomic organization among c.1.1 sequences closely resembles the consensus phylogeny generated from 520 genes from each species (21), which, together with the short branch lengths compared with other TPS-c clades, suggests that diTPSs in c.1.1 are highly conserved and evolve slowly. All three of the previously reported enzymes and 14 of 17 new candidates from c.1.1 contained a conserved histidine, which has been implicated in Mg^2+^-mediated inhibition of TPS-c enzymes involved in primary metabolism (Fig. S21) (51). The conserved histidine is also present in most enzymes in c.1.2 and 1.3, including PcTPS1, and the kolavenyl diphosphate synthases from *S. divinorum* and *V. agnus-castus*, demonstrating that it can also be present in enzymes of specialized metabolism (Fig. S21).

The remaining TPS-c clades contain genes involved in specialized metabolism. The only characterized gene from clade c.1.2 is PcTPS1, which makes an *ent-*labda-8,13E-dienyl diphosphate product (**25**). Enzymes from clade c.1.3 catalyze the formation of a variety of products, including *ent-*CPP (52), *ent*-8-LPP (**7**) (11), kolavenyl-PP (**36**), and *neo*-cleroda-4(18),13*E*-dienyl diphosphate (**38**). **36** and **38** are the only products without the labdane (Sk4) skeleton produced by Lamiaceae class II diTPSs. Compounds apparently derived from **36** are widespread among Lamiaceae (Fig. 4), so it is tempting to hypothesize that the progenitor of c.1.3 was a kolavenyl-PP synthase present in an early common ancestor. A histidine residue previously identified as important for coordination of water in the active site of *ent*-CPP synthases (53), but mutated to an aromatic amino acid in clerodenyl diphosphate synthases (17, 54, 55), was found as a histidine in all c.1.1 enzymes but as phenylalanine in ArTPS2 and PcTPS1 (Fig. S21). In *ent*-CPP synthases, the water acts as a base to abstract a proton from C-17, leading to the formation of a double bond between C-8 and C-17. The proposed mechanisms for ArTPS2 and PcTPS1 (Fig. 5) require proton abstraction at the C-18 and C-9 positions, respectively, and are consistent with disruption of the coordination of water near C-17 by mutation of the histidine into an aromatic amino acid.

The labdane compounds produced by enzymes in c.1 are all in the *ent*-configuration. On the other hand, with two exceptions, the known enzymes from clade c.2 all make products with the labdane skeleton in the normal configuration, suggesting that the founder of that clade may have been a normal configuration labdadiene diphosphate synthase. The exceptions are VacTPS3 (14), the only characterized member of c.2.1, which produces *syn*-CPP (**13**), and the curious case of SdCPS1 (17), which produces *ent-*CPP. Consistent with recent results regarding stereocontrol in Lamiaceae TPS-c enzymes (56), most of the enzymes in c.2 feature aromatic amino acids and histidine (Fig. S21), in place of the conserved histidine and arginine, respectively, of c.1.1.

Among TPS-e (class I) genes, all but one of the characterized enzymes from e.1 are *ent-*kaurene (**19**) synthases, presumably involved in gibberellin biosynthesis. As with the c.1.1 clade, e.1 reflects the taxonomic distribution among the species. Notable in e.1 are IrKSL4 (57), which is an *ent*-atiserene synthase, and SmKSL2 (11), which, in addition to *ent*-kaurene synthase activity, can convert *ent*-8-LPP **7** into *ent*-13-epi-manoyl oxide (**6**). In recent work (48, 58), *ent*-kaurene synthases from a broad range of species were found to have the ability to convert **7** to **6**, so it is likely that this is a general characteristic of all enzymes in the e.1 group. Conserved leucine and isoleucine residues previously implicated in *ent*-kaurene synthase activities (59, 60) were found in all but three of the new candidate sequences from e.1 (Fig. S22).

Most of the specialized class I diTPSs in the Lamiaceae fall into clade e.2. Enzymes in e.2 have lost the γ domain, present in many diTPSs and located on the opposite end of the peptide from the class I active site (57, 61). Characteristic of enzymes in e.2 is their ability to act on multiple substrates. The extreme example is SsSS (18), which so far has been able to catalyze the dephosphorylation and minor rearrangement of all class II enzyme products that it has been tested with (31, 48). The range of substrates accepted by other enzymes in this group has not been tested systematically, but among the e.2 enzymes characterized in this study, only one (OmTPS4) accepted *ent*-CPP, and all accepted (+)-CPP (**31**), (+)-8-LPP (**10**), and PgPP (**5**). There is great diversity among the products of e.2 enzymes, with over 20 distinct compounds represented. Most of the enzymes in e.2 convert (+)-CPP to miltiradiene (**32**) and (+)-8-LPP to 13*R*-(+)-manoyl oxide (**8**), with other activities arising sporadically across the clade. Both characterized enzymes in the Nepetoideae-specific e.2.2 have unusual activities; IrKSL6 converts (+)-CPP to isopimara-7,15-diene (**28**) (57), and OmTPS5 converts (+)-CPP to palustradiene (**29**). Most of the enzymes in e.2 fall into the e.2.1 clade, which also accounts for most of the known products. Enzymes that we characterized from e.2.1 lent support to emerging functionally consistent subclades. OmTPS4 activity, for three of four substrates tested, mimics that of its nearest homolog (SsSS), notably accepting *ent*-CPP as a substrate to produce *ent*-manool (**20**). LlTPS4 likewise has activities most similar to its closest homolog, MvELS (15), converting PgPP into 9,13(*S*)-epoxy-labd-14-ene (**2**) with greater specificity than other enzymes tested, although the products from (+)-CPP are different. From the remaining clade, e.2.3, the three characterized enzymes all come from Nepetoideae and convert (+)-CPP into different products: IrKSL3 produces miltiradiene (57), IrTPS2 produces nezukol (**30**) (62), and MsTPS1 produces sandaracopimaradiene (**27**). As noted earlier (63), the known activity-determining residues are almost completely replaced in e.2 compared with e.1. Superficially, there seems to be a correlation between differences in this region and differences in product specificity among e.2 enzymes (Fig. S22), but detailed mutational studies will be needed to assess the importance of individual residue switches.

The existence of two strongly supported subclades of specialized diTPSs within c.1, together with the presence of an *ent*-atiserene synthase in e.1, suggest that the emergence of specialized diTPSs from the *ent*-CPP and *ent*-kaurene synthases of gibberellin biosynthesis is an ongoing process that has occurred multiple times in the Lamiaceae lineage. Whereas it is evident that candidates selected from anywhere in the phylogenetic tree may have novel activities, clades that seem particularly promising and underexplored are c.2.1, c.1.2, and e.2.3. So far, including this work and previous work, diTPSs have been characterized from only four of the 12 major Lamiaceae clades: Ajugoideae, Lamioideae, Nepetoideae, and Viticoideae. Further expanding to the remaining eight Lamiaceae clades may also be a promising strategy for finding new enzyme activities.

### The diterpene skeletons of Lamiaceae and how to make them

By considering our newly characterized enzyme activities in the context of chemotaxonomic data and previously described enzymes, we can make some informed speculations about how diverse skeletons arise and what strategies may be used for finding more of the enzymes responsible. All of the six diterpene skeletons with a known biosynthetic route in the Lamiaceae contain a decalin core; Sk2, and Sk4 (Fig. 1, *C* and *D*) are skeletons of the direct products of TPS-c enzymes, whereas Sk1, Sk3, Sk6, and Sk14 are skeletons of the products of a TPS-e enzyme acting on a labdadiene diphosphate (Sk4) precursor.

Many diterpene skeletons with an intact decalin core can be plausibly explained by as-yet-undiscovered diTPSs from the TPS-c and TPS-e subfamilies (*e.g.* through methyl shifts during cyclization). Examples of diTPSs that catalyze methyl shifts are the TPS-c enzymes SdKPS (16, 17) and ArTPS2, which produce the clerodane skeleton (Sk2), and the TPS-e enzyme OmTPS5, which has a product with the abietane skeleton (Sk3). The same mechanisms may form skeletons such as Sk8 and Sk12. Other decalin-containing skeletons, for example the norditerpenes (missing one or more methyl side chains (*e.g.* Sk7)), are readily explainable by oxidative decarboxylation occurring after the TPS steps. Ring rearrangements catalyzed by TPS-e enzymes also have precedent, for example the generation of *ent*-kaurene (with skeleton Sk1) or *ent*-atiserene (with skeleton Sk14) from *ent*-CPP (with skeleton Sk4) (2, 57), but always preserve the decalin core structure.

Diterpenoids lacking a decalin core are taxonomically restricted within the Lamiaceae, with no single skeleton being reported in more than two clades (Fig. 1*C*). Many can be explained as modifications occurring after the TPS steps to decalin-containing skeletons. Cytochrome P450–driven ring contraction, akin to that in the gibberellin pathway (64), may play a role in the formation of skeletons such as Sk13 (65). Ring opening and ring expansion may also occur, for example in proposed pathways to compounds with the 6,7-*seco-*kaurane (Sk5) (66) and icetaxane (Sk9) (67) skeletons, respectively. Skeletons such as cembrane (Sk11) (68, 69), lacking any apparent biosynthetic connection to a decalin core, may arise from diTPSs outside the TPS-c and TPS-e subfamilies. In Euphorbiaceae and Solanaceae, where cembranoid compounds are common, the relevant TPSs come from the TPS-a subfamily (70, 71). Elucidation of pathways to the remaining diterpene skeletons in the Lamiaceae will depend on broadening the search to new genera and species and new TPS subfamilies, eventually moving beyond TPSs to look at cytochromes P450 and other enzyme families.

### Implications for biotechnology

Previous work has explored the possibility of producing arrays of compounds by combining class II diTPSs with different class I diTPSs (31, 48). Particularly prolific enzymes for combinatorial biosynthesis have been Cyc2 from the bacterium *Streptomyces griseolosporeus* (72, 73), which generates alkene moieties on prenyl-diphosphate substrates, and SsSS (18, 20) which installs an alcohol at the 13-position and a double bond at the 14-position; both of these enzymes have demonstrated activity on 12 different class II enzyme products (31). We have found that SsSS is also active on the products of PcTPS1 and ArTPS2. In addition, we have found class I enzymes that provide routes to products that previously were biosynthetically inaccessible or poorly accessible. OmTPS3 is active on class II products with a labdane skeleton and normal absolute configuration, typically generating a 12*E*-moiety, as in **11**, **34**, and **24**. An enzyme with similar activity, producing **24** and **34**, was recently reported from the bacterium *Streptomyces cyslabdanicus* (36, 74), but was not tested against additional substrates. LlTPS4 produces sandaracopimaradiene (**27**) from **31**, with greater specificity than the earlier enzyme, *Euphorbia peplus* TPS8 (48). Finally, OmTPS5 enables efficient and specific production of palustradiene (**29**) from **31.** The other known biosynthetic route to **29** is as a minor spontaneous degradation product of 13-hydroxy-8(14)-abietane from *Picea abies* levopimaradiene/abietadiene synthase (75) and related enzymes.

**Figure 8.**
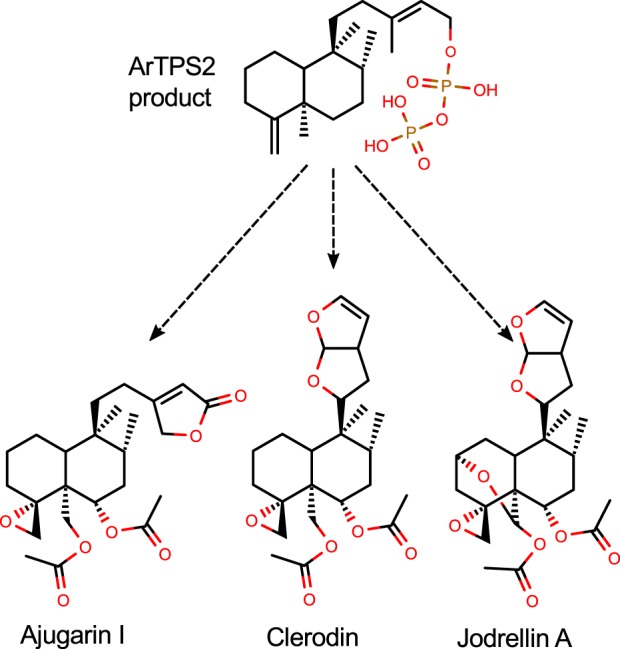
**The ArTPS2 product, (5*R*,8*R*,9*S*,10*R*)-*neo*-cleroda-4(18),13*E*-dienyl diphosphate, is the likely precursor to bioactive epoxy-clerodane diterpenoids.**

ArTPS2 is of particular interest for applications in agricultural biotechnology. *Neo*-clerodane diterpenoids, particularly those with an epoxide moiety at the 4(18) position, such as clerodin, the ajugarins, and the jodrellins (76–78) (Fig. 8), have garnered significant attention for their ability to deter insect herbivores (22–24). The 4(18) desaturated product of ArTPS2, for which no enzyme was previously known, could be used in biosynthetic or semisynthetic routes to these potent insect antifeedants.

### Database-driven phytochemistry

In the traditional plant natural product discovery workflow, compounds are first identified from bulk extractions, and only later, often by decades, are the associated biosynthetic genes identified. In recent years, rapid advances in genome sequencing and transcript profiling have made it much easier and cheaper to obtain detailed genomic data than to obtain comprehensive metabolite data from an organism. A number of strategies have been developed for leveraging genomic data to find novel enzymes, which may or may not be part of pathways to known compounds. Mining of genomes, metagenomes, and metatranscriptomes has led to the identification, from plants, bacteria, and fungi, of natural product pathways that would not have been possible in a metabolomics-first approach (79). In plant terpenoid pathways, genome mining has been used to identify prenyltranferases, cytochromes P450, and TPSs, which have been observed to sometimes occur in genomic clusters (80). In the same spirit as these earlier studies, we took advantage of recently available Lamiaceae leaf transcriptomes to mine for diTPS sequences. Genomics-first approaches may lead back to metabolomics studies, as was the case when we found palustradiene in our *O. majorana* individual. Alternatively, metabolomics in the source species may be bypassed entirely, with new compounds moved directly to screening for useful biological activities.

In genomics-driven pathway discovery workflows, there is a temptation to not pay much attention to chemotaxonomic data. Part of the reason may be the lack of a comprehensive, user-friendly, open-access database of natural product distribution. Despite this obstacle, in this work we have tried to give equal attention to the decades of accumulated data on natural product distribution as we have to the genomics data. We have found that the chemotaxonomic analysis gives valuable context to enzyme function data (Fig. 4), and its availability helped with interpreting our multispecies diTPS phylogenetic tree and suggesting promising species and genera to target in the future. Aside from its application to terpenoid-related gene discovery, large scale chemotaxonomic analysis has also recently been used to help understand specialty wax biosynthetic pathways across the plant kingdom (81).

Considering that transcriptome data sets for thousands of plant species are already available in public databases, such as the NCBI Sequence Read Archive (82) and Transcriptome Shotgun Assembly (83) archive, and the influx of new data sets is only getting faster, our success in mining the transcriptomes of just 48 species suggests that additional systematic mining of existing transcriptome data could yield many more examples of diverse TPSs and other kinds of enzymes. Systematic, broad transcriptome sampling of additional plant families will help give context to transcriptome mining and improve the chances of finding new enzyme activities. To further enable others to build off our work, we have included detailed data tables of Lamiaceae diTPSs and skeletons as supporting data sets, and, to the extent possible, we have made the data and code used in this study publicly available.

## Experimental procedures

### Chemotaxonomic database curation

A subset of the NAPRALERT database including all of the occurrences of diterpenoids in mints was obtained as a gift from James Graham (University of Illinois, Chicago, IL). NAPRALERT reports chemical names, but not structures. For the Lamiaceae, the species reported in NAPRALERT largely overlap with those from the DNP, which does include structures, so we made the simplifying assumption that each unique name represents a unique compound, without trying to find structures for the 3,080 Lamiaceae diterpenes in NAPRALERT.

For SISTEMAT, we obtained structure files by redrawing the structures from Vestri Alvarenga *et al.* (25) into MarvinSketch (ChemAxon, Budapest, Hungary) and the occurrence counts by transcribing the association table into a spreadsheet. A publicly available digital version of SISTEMAT, called SISTEMATX (https://www.sistematx.ufpb.br/),^5^ exists (84), but there is no option for bulk downloads, limiting our ability to assess its completeness or cross-reference it with other data. We hope that in the future, a publicly available digital chemotaxonomy resource such as SISTEMAT X develops to point where it can be used in gene discovery workflows, but for the present work, the proprietary DNP seemed to be the only viable option for most analyses.

Lamiaceae diterpene structures were obtained from the DNP by searching for them through the DNP web interface (1). Additional compounds were found by searching for individual species names of species for which we had transcriptome data. This additional search step was necessary because some species have been reclassified between families, or their family is not correctly annotated in the DNP. Records for all of the Lamiaceae diterpenes were downloaded and converted into a spreadsheet using a Python script. Species names were extracted from the Biological Source field in a semiautomated method. The DNP contains structural information in the form of InChI strings (85). In most cases, the DNP InChIs do not include stereochemical information, so for consistency, we ignored all stereochemical information. Skeletons were extracted from the structures using the RDKit Python interface. Briefly, all bonds were converted into single bonds, bonds involving at least one noncarbon atom were broken, and the fragment with a carbon count closest to 20 was retained as the skeleton. The resulting skeletons were then manually examined to correct those where the algorithm chose the wrong fragment; for example, a small number of diterpenoids contain acyl side chains of more than 20 carbons, in which case the algorithm would incorrectly select the acyl chain as the skeleton. There are a few cases where sesquiterpenes or other terpenes seem to have been misannotated in the DNP as diterpenes. We chose to leave these in the data set, but their presence or absence does not significantly change any of our analyses.

For all three databases, genus and species names were cross-referenced to TaxIDs from the NCBI Taxonomy database (27), first by automated text comparisons and then by manual inspection of unmatched names. Genus-level TaxID assignments were possible for every entry in NAPRALERT and the DNP, but in some cases, species-level TaxID assignments were not possible.

### Candidate gene selection

TransDecoder (version 4.1.0) (86) was used to predict coding sequences from the transcriptome assemblies of the 48 Lamiaceae species. Peptides with 95% or greater identity were merged with CD-HIT (87). The set of known Lamiaceae diTPSs was used as a BLASTP query set against the new peptide sequences. Candidates were filtered to remove sequences with less than 70% coverage or 35% identity to the most similar query peptide. All specific parameters are given in supporting Data set S2, candidate search methods.

To generate phylogenetic trees, peptide sequences were aligned using Clustal Omega (version 1.2.1) (88), and maximum likelihood trees were generated using RAxML (version 8.2.11) (89) using automatic model selection and 1,000 bootstrap iterations. Tree visualizations were generated using ETE3 (90).

Candidates were selected for cloning and characterization based on visual inspection of the phylogenetic trees with the intent to select genes from underrepresented clades. Candidate selection was also influenced by our ability to obtain plant material and by the reported diversity, or lack thereof, of diterpenes in particular genera and species.

### Plant material, RNA isolation, and cDNA synthesis

Plants were obtained from different commercial nurseries or botanical gardens (Table S1) and grown in a greenhouse under ambient photoperiod and 24 °C day/17 °C night temperatures. *N. benthamiana* were grown in a greenhouse under a 16-h light (24 °C) and 8-h dark (17 °C) regime.

Total RNA from leaf tissues of *A. reptans*, *Nepeta mussini*, *Leonotis leonurus*, *Perovskia atriplicifolia*, and *S. officinalis* was extracted according to Hamberger *et al.* (91), whereas total RNA from leaves of *Proteus vulgaris*, *M. spicata*, *P. cablin*, *H. suaveolens*, and *O. majorana* was extracted using the Spectrum Plant Total RNA Kit (Sigma-Aldrich). RNA extraction was followed by DNase I digestion using the DNA-free^TM^ DNA removal kit (Thermo Fisher Scientific). First-strand cDNAs were synthesized from 5 μg of total RNA, with oligo(dT) primer, using the RevertAid First Strand cDNA synthesis kit (Thermo Fisher Scientific). cDNA was diluted 5-fold and used as template for cloning of full-length cDNAs.

### Characterization of diTPS genes by transient expression in N. benthamiana

Full-length coding sequences of diTPSs were cloned into pEAQ-HT vector (92) (kindly provided by Prof. G. Lomonossoff, John Innes Centre, UK) using In-Fusion® HD Cloning Plus (Takara Bio). pEAQ-HT vector contains a copy of anti-post-transcriptional gene silencing protein p19 that suppresses the silencing of transgenes (92). Expression vectors carrying full-length coding sequence of candidate diTPS genes were transformed into the LBA4404 *Agrobacterium tumefaciens* strain by electroporation. DXS and GGPPS are known to be the rate-limiting enzymes in GGPP biosynthesis and have been shown to substantially increase the production of diterpenes in the *N. benthamiana* system (30, 48). We therefore cloned *P. barbatus* 1-deoxy-d-xylulose 5-phosphate synthase (CfDXS) (GenBank^TM^ accession number KP889115) and geranylgeranyl diphosphate synthase (CfGGPPS) (GenBank^TM^ accession number KP889114) and created a chimeric polyprotein with an LP4-2A hybrid linker peptide between CfDXS and CfGGPPS. LP4/2A contains the first nine amino acids of LP4 (a linker peptide originating from a natural polyprotein occurring in seeds of *Impatiens balsamina*) and 20 amino acids of the self-processing FMDV 2A (2A is a peptide from the foot-and-mouth disease virus) (94).

The transformed *A. tumefaciens* were subsequently transferred to 1 ml of SOC medium and grown for 1 h at 28 °C. 100-μl cultures were transferred to LB-agar solid medium containing 50.0 μg/ml rifampicin and 50.0 μg/ml kanamycin and grown for 2 days. A single-colony PCR-positive clone was transferred to 10 ml of LB medium in a Falcon tube containing 50.0 μg/ml rifampicin and 50.0 μg/ml kanamycin and grown at 28 °C overnight (at 225 rpm). About 1% of the primary culture was transferred to 25 ml of fresh LB medium and grown overnight. Cells were pelleted by centrifugation at 4,000 × *g* for 15 min and resuspended in 10 ml of water containing 200 μm acetosyringone. Cells were diluted with water-acetosyringone solution to a final *A*_600_ of 1.0 and incubated at 28 °C for 2–3 h to increase the infectivity. Equal volumes of culture containing the plasmids with cDNA encoding different diTPS genes were mixed. Each combination of *A. tumefaciens* culture mixture was infiltrated into independent 4–5-week-old *N. benthamiana* plants. Plants were grown for 5–7 days in the greenhouse before metabolite extraction. Leaf discs of 2-cm diameter (∼0.1 g fresh weight) were cut from the infiltrated leaves. Diterpenes were extracted in 1 ml of *n*-hexane with 1 mg/liter 1-eicosene as internal standard at room temperature overnight in an orbital shaker at 200 rpm. Plant material was collected by centrifugation, and the organic phase was transferred to GC vials for analysis.

### In vitro enzyme activity assays

To confirm the biosynthetic products obtained in *N. benthamiana*, diTPS combinations were tested in *in vitro* assays as described previously (30). TargetP (95) was used for prediction of the plastidial target sequence. Pseudo-mature variant versions of HsTPS1, ArTPS2, PcTPS1, OmTPS3, OmTPS5, SsSS, CfTPS1, CfTPS2, and codon-optimized CfTPS3 (IDT), lacking the predicted plastidial targeting sequences, were cloned in pET-28b(+) (EMD Millipore, Burlington, MA), expressed, and purified from *E. coli*. pET_diTPS constructs were transformed into chemically competent OverExpress^TM^ C41(DE3) cells (Lucigen, Middleton, WI) and inoculated in a starter culture with Terrific Broth medium and 50 μg/ml kanamycin and grown overnight. About 1% of the starter culture was used to inoculate 50 ml of Terrific Broth medium with 50 μg/ml kanamycin and grown at 37 °C and 200 rpm until *A*_600_ reached 0.4. Cultures were grown at 16 °C until *A*_600_ of ∼0.6–0.8 was achieved, at which point cultures were induced by 0.2 mm isopropyl 1-thio-β-d-galactopyranoside. Expression was done overnight, and cells were harvested by centrifugation at 5,000 × *g* at 4 °C for 15 min. Cell pellets were resuspended in lysis buffer containing 20 mm HEPES, pH 7.5, 0.5 m NaCl, 25 mm imidazole, 5% (v/v) glycerol, one protease inhibitor mixture tablet per 100 ml (Sigma-Aldrich), and 0.1 mg/liter lysozyme. The cell pellet was gently shaken for 30 min and subsequently lysed by sonication. Cell lysate was centrifuged for 25 min at 14,000 × *g*, and the supernatant was subsequently used for purification of the recombinant proteins. Proteins were purified on 1-ml His SpinTrap columns (GE Healthcare) using elution buffer (HEPES, pH 7.5, 0.5 m NaCl, 5% (v/v) glycerol, 350 mm imidazole, and 5 mm DTT) and desalted on PD MiniTrap G-25 columns (GE Healthcare) with a desalting buffer (20 mm HEPES, pH 7.2, 350 mm NaCl, 5 mm DTT, 1 mm MgCl_2_, 5% (v/v) glycerol). *In vitro* diTPS assays were performed by adding GGPP to a final concentration of 15 μm and 50–100 μg of purified enzymes in 400 μl of enzyme assay buffer (50 mm HEPES, pH 7.2, 7.5 mm MgCl_2_, 5% (v/v) glycerol, 5 mm DTT). 500 ml of *n*-hexane (Fluka GC-MS grade) containing 1 ng/ml 1-eicosene as internal standard were gently added as an overlay onto the reaction mix. Assays were incubated for 60–120 min at 30 °C and ∼75 rpm, and the hexane overlay was subsequently removed by centrifugation at 1,500 × *g* for 15 min before GC-MS analysis.

### Metabolite analysis of O. majorana

20–50 mg of fresh leaf, stem, root, and flowers of *O. majorana* were harvested. Flowers were further separated with forceps into two parts: the green part (calyx) and the rest of the flower (corolla). Tissues were extracted overnight in 500 μl of methyl *tert*-butyl ether. Extracts were concentrated to 100 μl and subjected to GC-MS analysis.

### GC-MS

All GC-MS analyses were performed on an Agilent 7890A GC with an Agilent VF-5ms column (30 m × 250 μm × 0.25 μm, with 10-m EZ-Guard) and an Agilent 5975C detector. For *N. benthamiana* and *in vitro* assays, the inlet was set to 250 °C splitless injection, helium carrier gas with column flow of 1 ml/min. The oven program was 45 °C hold 1 min, 40 °C/min to 230 °C, 7 °C/min to 320 °C, hold 3 min. The detector was activated after a 4-min solvent delay. For analysis of *O. majorana* extracts, conditions were the same, except that the solvent cutoff was set to 6 min to allow monoterpenes to pass, and the oven program was 45 °C hold 1 min, 40 °C/min to 200 °C, 5 °C/min to 260 °C, 40 °C/min to 320 °C, hold 3 min.

### Large-scale production of diterpenes in N. benthamiana for NMR analysis

To produce diterpene levels sufficient for structural analysis by NMR, we made several modifications to the experimental setup of the *N. benthamiana* system infiltration. *A. tumefaciens* cultures (500 ml) containing HsTPS1, ArTPS2, CfTPS1, CfTPS2, CfTPS3, PcTPS1, OmTPS3, OmTPS5, and SsSS constructs were separately grown overnight from 20-ml starter cultures and pelleted by centrifugation. Cell pellets were resuspended in water and adjusted to an *A*_600_ of 1.0. Resuspended cells were mixed according to the selected combinations together with strains harboring CfDXS/CfGGPPS polyprotein. *N. benthamiana* plants were submerged in the *Agrobacterium* suspensions and vacuum-infiltrated at 100 mbar for 30–60 s, similar to a method described previously (48). 15–30 *N. benthamiana* plants were vacuum-infiltrated with diTPS combinations as well as CfGGPPS and CfDXS. After 5 days, 100–200 g (fresh weight) of leaves were subjected to two rounds of overnight extractions in 500 ml of hexane, which was then concentrated using a rotary evaporator. Compounds were purified on silica gel columns using a mobile phase of hexane with 0–20% ethyl acetate. In some cases, additional rounds of column purification or preparative TLC using a hexane/ethyl acetate or chloroform/methanol mobile phase were necessary to obtain compounds of sufficient purity for structural determination by NMR.

### NMR and optical rotation

The NMR spectra for *trans*-biformene (**34**) were measured on a Bruker AVANCE 900-MHz spectrometer. All other spectra were measured on an Agilent DirectDrive2 500-MHz spectrometer. All NMR was done in CDCl_3_ solvent. The CDCl_3_ peaks were referenced to 7.24 and 77.23 ppm for ^1^H and ^13^C spectra, respectively. To aid in the interpretation of NMR spectra, we made extensive use of the NAPROC-13 (96) and Spektraris (97) databases. Reconstruction of ^13^C spectra from the literature was performed with MestReNova (Mestrelab Research, Santiago de Compostela, Spain). Optical rotation was measured in chloroform at ambient temperature using a PerkinElmer Life Sciences Polarimeter 341 instrument.

### Availability of data

GenBank^TM^ numbers for new clones are provided in Data set S1. For code and kinds of data not appropriate for supporting data, and where no appropriate centralized repository exists, we have used the generic database Zenodo. Our Zenodo submission (doi: 10.5281/zenodo.1323366) includes all of the NMR and GC-MS data for this study in multiple formats, as well as the code used to generate the figures. We have also submitted our shift-assigned NMR data for inclusion in the NMRShiftDB (93) database. We are not aware of an open access electron ionization-MS library that accepts submissions from the public, so we have made an AMDIS msl formatted library of background-subtracted reference spectra for compounds observed by GC-MS during this study. The GC-MS library is included in the Zenodo submission and is also available in a Git repository (https://bitbucket.org/seanrjohnson/diterpenoid_databases),^5^ which we will continue updating with spectra generated in future studies.

## Author contributions

S. R. J. and Björn Hamberger conceptualization; S. R. J. resources; S. R. J., W. W. B., and E. M. G. C. data curation; S. R. J. software; S. R. J. and W. W. B. formal analysis; S. R. J., W. W. B., and J. B. validation; S. R. J. visualization; S. R. J. and W. W. B. methodology; S. R. J. writing-original draft; S. R. J., W. W. B., and Björn Hamberger writing-review and editing; W. W. B., J. B., A. T., and Britta Hamberger investigation; Björn Hamberger supervision; Björn Hamberger funding acquisition.

## Supplementary Material

Supporting Information

## Footnotes

5 Please note that the JBC is not responsible for the long-term archiving and maintenance of this site or any other third party hosted site.

## Footnotes

4 The abbreviations used are:

DNP: Dictionary of Natural Products
diTPS: diterpene synthase
GGPP: geranylgeranyl diphosphate
CPP: copalyl diphosphate
PgPP: peregrinol diphosphate
MvELS: *M. vulgare* 9,13-epoxylabdene synthase
SsSS: *S. sclarea* sclareol synthase
CfDXS: 1-deoxy-d-xylulose 5-phosphate synthase
CfGGPS: geranylgeranyl diphosphate synthase
LB: lysogeny broth.
