# Supplementary material for: A database-driven approach identifies additional diterpene synthase activities in the mint family (Lamiaceae)
Source: J Biol Chem. 2018 Nov 29;294(4):1349–62. doi: 10.1074/jbc.RA118.006025 (PMC6349103; doi:10.1074/jbc.RA118.006025)
Supplement: Supporting Information [file supp_294_4_1349__index.html]

A database-driven approach identifies additional diterpene synthase activities in the mint family (Lamiaceae) — Mint diterpene synthases — Supporting Information 

# A database-driven approach identifies additional diterpene synthase activities in the mint family (Lamiaceae)

## Supporting Information

- Supporting Information (to be published online) - Supplementary material
- Dataset S1
- Dataset S2
- Dataset S3
