# Supplementary material for: A database-driven approach identifies additional diterpene synthase activities in the mint family (Lamiaceae)
Source: J Biol Chem. 2018 Nov 29;294(4):1349–62. doi: 10.1074/jbc.RA118.006025 (PMC6349103; doi:10.1074/jbc.RA118.006025)
Supplement: Supporting Information [file supp_RA118.006025_141011_1_supp_233936_ph45w2.pdf]

A database-driven approach identifies additional diterpene synthase activities in Lamiaceae

Sean R. Johnson, Wajid Waheed Bhat, Jacob Bibik, Aiko Turmo, Britta Hamberger, Evolutionary Mint Genomics Consortium, Björn Hamberger

Table S1. Sources of plants used in this study.

Table S2. List of Primers used in this study.

Table S3. Index of class I diTPS functional assays by N. benthamiana transient expression.

Table S4. Index of class II diTPS functional assays by *N. benthamiana* transient expression.

Table S5. Index of *in-vitro* assays.

Figure S1. An example of skeleton extraction.

Figure S2. Pathway diagram of newly characterized enzyme activities

Figure S3. Class I diTPS *N. benthamiana* assays with (+)-CPP.

Figure S4. Class I diTPS *N. benthamiana* assays with (+)-8-LPP.

Figure S5. Class I diTPS *N. benthamiana* assays with PgPP.

Figure S6. Class I diTPS *N. benthamiana* assays with *ent*-CPP.

Figure S7. (+)-CPP synthase *N. benthamiana* assays.

Figure S8. Other class II diTPS *N. benthamiana* assays.

Figure S9. Comparison of *in-vitro* and *in-vivo* activities for selected combinations.

Figure S10. NMR of trans-abienol [**11**]

Figure S11. NMR of labda-7,13E-dien-15-ol [**21a**]

Figure S12. NMR of labda-7,13(16),14-triene [**22**]

Figure S13. NMR of labda-7,12E,14-triene [**24**]

Figure S14. NMR of (10R)-labda-8,13E-diene-15-ol [**25a**]

Figure S15. NMR of (10R)-labda-8,14-dien-13-ol [**26**]

Figure S16. NMR of trans-biformene [**34**]

Figure S17. NMR of neo-cleroda-4(18),14-dien-13-ol [**37**]

Figure S18. NMR of neo-cleroda-4(18),13E-diene-15-ol [**38a**]

Figure S19. NOESY of (+)-cis-abienol

Figure S20. Phylogenetic tree of all known and candidate diTPSs from Lamiaceae

Figure S21. Activity-determining regions in an alignment of previously known, newly characterized, and candidate TPS-c enzymes from Lamiaceae

Figure S22. An activity-determining region in an alignment of previously known, newly characterized, and candidate TPS-e enzymes from Lamiaceae

Taxon	Subfamily	Source
Ajuga reptans L.	Ajugoideae	Horizon Herbs, Williams, Oregon, USA
Hyptis suaveolens (L.) Poit.	Nepetoideae	Native seeds, Tucson, Arizona, USA
Leonotis leonurus (L.) R.Br.	Lamioideae	Logee's Greenhouses, Danielson, Connecticut, USA
Mentha spicata L.	Nepetoideae	Richters Herbs, Goodwood, Ontario, Canada
Nepeta mussinii Spreng. ex Henckel.	Nepetoideae	Outside Pride, Independence, Oregon, USA
Origanum majorana L.	Nepetoideae	Richters Herbs, Goodwood, Ontario, Canada
Perovskia atriplicifolia Benth.	Nepetoideae	Department of Horticulture, Michigan State University (https://www.canr.msu.edu/hrt/)
Plectranthus barbatus	Nepetoideae	Companion Plants, Athens, Ohio, USA
Pogostemon cablin (Blanco) Benth.	Lamioideae	Richters Herbs, Goodwood, Ontario, Canada
Prunella vulgaris L.	Nepetoideae	WJ Beal Botanical Garden, Michigan State University
Salvia officinalis L.	Nepetoideae	Department of Horticulture, Michigan State University (https://www.canr.msu.edu/hrt/)

Table S1. Sources of plants used in this study.

Name	**Sequence**	***Gene of interest***
Amplification of full length genes from cDNA synthesized from plant tissues total RNA		
ZmAN2-F	ATGGTTCTTTCATCGTCTTGCACA	*ZmAN2*
ZmAN2-R	TTATTTTGCGGCGGAAACAGGTTCA	
CfTPS2-F	AGATTGAGGATTCCATTGAGTACGTGAAGG	*CfTPS2*
CfTPS2-R	GAAGTTTAATATCCTTCATTCTTTATTACA	
CfTPS3-F	AGCTCCATTCAACTAGAGTCATGTCGT	*CfTPS3*
CfTPS3-R	TTCATCTGGCTTAACTAGTTGCTGACAC	
CfTPS16-F	TTAAAGTACTCTCTCAAAGAGTACTTTGG	*CfTPS16*
CfTPS16-R	GCGACCAACCATCATACGACT	
LlTPS1-F	AATGGCCTCCACTGCATCCACTCTA	*LlTPS1*
LlTPS1-R	CCATACTCATTCAACTGGTTCGAACA	
LlTPS4-F	AGCCTGTGTACTCGAAATGTC	*LlTPS4*
LlTPS4-R	CAAGAGGATGATTCATGTACCAAC	

SoTPS1-F	TCTCTTTCAAGAATATCCCCTCTC	*SoTPS1*
SoTPS1-R	GGCATTCAATGATTTTGAGTCG	
ArTPS1-F	AAATGGCCTCTTTGTCCACTCTC	*ArTPS1*
ArTPS1-R	TTACGCAACTGGTTCGAAAAGCA	
ArTPS2-F	TAATGTCATTTGCTTCCCAAGCCA	*ArTPS2*
ArTPS2-R	GGCCTAGACTACCTTCTCAAACAA	
ArTPS3-F	AATGTCACTCTCGTTCACCATCAA	*ArTPS3*
ArTPS3-R	ACTTCAAGAGGATGAAGTGTTTAGG	
PaTPS1-F	CTCCAAAACTCGGGCCGGTAAAT	*PaTPS1*
PaTPS1-R	TACGTATTTCCTCACAATCGAGCA	
PaTPS3-F	CTAGAAATGTTACTTGCGTTCAAC	*PaTPS3*
PaTPS3-R	GGGTAAGAGTTGAATTTAGATGTCT	
NmTPS1-F	ATGACTTCAATATCCTCTCTAAATTTGAGC	*NmTPS1*
NmTPS1-R	GAATATAGTAATCAGACGACCGGTCCA	
NmTPS2-F	GCCATATCATGTCTCTTCCGCTCT	*NmTPS2*
NmTPS2-R	TTATTCATGCACCTTAAAATCCTTGAGAG	
OmTPS1-F	ATGACCGATGTATCCTCTCTTCGT	*OmTPS1*
OmTPS1-R	AAACACTCACATAACCGGCCCAA	
OmTPS3-F	GTCCTTGCTTTCGGAATACT	*OmTPS3*
OmTPS3-R	GAAGTGATCTACAAGGATTCATAAA	
OmTPS4-F	TCATTGATTTGCCCTGCATCCAC	*OmTPS4*
OmTPS4-R	CAAAGCTAGTGCTGCTTCTGATT	
OmTPS5-F	ATGGTATCTGCATGTCTAAAACTCAA	*OmTPS5*
OmTPS5-R	CTTTCTCTCTCTTGTGCATCTTAGT	
MsTPS1-F	ACGTTCATCTTCAATGAGTTCCA	*MsTPS1*
MsTPS1-R	TACGTGTATGTCGATCTGTTCCAAT	
PcTPS1-F	CATGTCATTTGCTTCTCAATCAC	*PcTPS1*
PcTPS1-R	CCCATTATCTAAAAGTCTACATCACC	
HsTPS1-F	TCCTCATAAAGCAATGGCGTATA	*HsTPS1*
HsTPS1-R	CTAAGATTCAGACAATGGGCTCA	
EpTPS8-F	GCAGACGCCAATCTTTCTTGGT	*EpTPS8*
EpTPS8-R	TTATGAAGTTAAAAGGAGTGGTTCGTTGAC	
PVTPS1-F	GGAACGAGAAATGTCACTCAC	*PVTPS1*
PVTPS1-R	TTCTAGTTTCTCACAGAAGTCAA	
LP4-2A Ver.1 sequence	TCAAATGCAGCAGACGAAGTTGCTACTCAACTTTTGAATTTTGACTTGCTGAAGTTGGCTGGTGATGTTGAGTCAAACCCTGGACCT	Synthesized by Integrated DNA Technologies, Skokie, Illinois, USA
**Cloning of full length diTPS genes into pEAQ-HT for transient expression in *N. benthamiana***		

pEAQ_Infusion _CfTPS1-F	TTCTGCCCAAATTCGATGGGGTCTCTATCCACTATGA	*CfTPS1*
pEAQ_Infusion _CfTPS1-R	AGTTAAAGGCCTCGATCAGGCGACTGGTTCGAAAAGTA	
pEAQ_Infusion _SsSCS-F	TTCTGCCCAAATTCGATGTCGCTCGCCTTCAAC	*SsSS*
pEAQ_Infusion _SsSCS-R	AGTTAAAGGCCTCGATCAAAAGACAAAGGATTTCATA	
pEAQ_Infusion _ZmAN2-F	TTCTGCCCAAATTCGATGGTTCTTTCATCGTCTTGCAC	*ZmAN2*
pEAQ_Infusion _ZmAN2-R	AGTTAAAGGCCTCGATTATTTTGCGGCGGAAACAGGT	
pEAQ_Infusion _CfTPS2-F	TTCTGCCCAAATTCGATGAAAATGTTGATGATCAAAAGT	*CfTPS2*
pEAQ_Infusion _CfTPS2-R	AGTTAAAGGCCTCGATCAGACCACTGGTTCAAATAGTA	
pEAQ_Infusion _CfTPS3-F	TTCTGCCCAAATTCGATGTCGTCCCTCGCCGGCAACCT	*CfTPS3*
pEAQ_Infusion _CfTPS3-R	AGTTAAAGGCCTCGACTAGTTGCTGACACAACTCATT	
pEAQ_Infusion _CfTPS16-F	TTCTGCCCAAATTCGATGCAGGCTTCTATGTCATCT	*CfTPS16*
pEAQ_Infusion _CfTPS16-R	AGTTAAAGGCCTCGATCATACGACTGGTTCAAACATT	
pEAQ_Infusion _LlTPS1-F	TTCTGCCCAAATTCGATGGCCTCCACTGCATCC	*LlTPS1*
pEAQ_Infusion _LlTPS1-R	AGTTAAAGGCCTCGATCATTCAACTGGTTCGAACAA	
pEAQ_Infusion _LlTPS2-F	TTCTGCCCAAATTCGATGATTCCTAATCCCGAAA	*LlTPS2*
pEAQ_Infusion _LlTPS2-R	AGTTAAAGGCCTCGATTACATTGGCAATCCGATGAA	
pEAQ_Infusion _LlTPS4-F	TTCTGCCCAAATTCGATGTCGGTGGCGTTCAACCT	*LlTPS4*
pEAQ_Infusion _LlTPS4-R	AGTTAAAGGCCTCGATCAAGAGGATGATTCATGTACC	
pEAQ_Infusion _SoTPS1-F	TTCTGCCCAAATTCGATGTCCCTCGCCTTCAACG	*SoTPS1*
pEAQ_Infusion _SoTPS1-R	AGTTAAAGGCCTCGATCATTTGCCACTCACATTT	
pEAQ_Infusion _ArTPS1-F	TTCTGCCCAAATTCGATGGCCTCTTTGTCCACTTTCC	*ArTPS1*
pEAQ_Infusion _ArTPS1-R	AGTTAAAGGCCTCGATCACGCAACTGGTTCGAAAAGA	
pEAQ_Infusion _ArTPS2-F	TTCTGCCCAAATTCGATGTCATTTGCTTCCCAAGCCAC	*ArTPS2*
pEAQ_Infusion _ArTPS2-R	AGTTAAAGGCCTCGACTAGACTACCTTCTCAAACAATAC	
pEAQ_Infusion _ArTPS3-F	TTCTGCCCAAATTCGATGTCACTCTCGTTCACCATCA	*ArTPS3*
pEAQ_Infusion _ArTPS3-R	AGTTAAAGGCCTCGATCAAGAGGATGAAGTGTTTAG	

pEAQ_Infusion _PaTPS1-F	TTCTGCCCAAATTCGATGACCTCTATGTCCTCTCTAA	*PaTPS1*
pEAQ_Infusion _PaTPS1-R	AGTTAAAGGCCTCGATCATACGACCGGTCCAAACAGT	
pEAQ_Infusion _PaTPS3-F	TTCTGCCCAAATTCGATGTTACTTGCGTTCAACATAAGC	*PaTPS3*
pEAQ_Infusion _PaTPS3-R	AGTTAAAGGCCTCGATTAATTAGGTAGGTAGAGGGGTT	
pEAQ_Infusion _NmTPS1-F	ATATTCTGCCCAAATTCGATGACTTCAATATCCTCTCTAAATTTGAGCAATG	*NmTPS1*
pEAQ_Infusion _NmTPS1-R	CAGAGTTAAAGGCCTCGATCAGACGACCGGTCCAA	
pEAQ_Infusion _NmTPS2-F	TTCTGCCCAAATTCGATGTCTCTTCCGCTCTCCTCT	*NmTPS2*
pEAQ_Infusion _NmTPS2-R	GATAAGTTAAAGGCCTCGATTATTCATGCACCTTAAAATCCTTGAGAGC	
pEAQ_Infusion _OmTPS1-F	TTCTGCCCAAATTCGATGACCGATGTATCCTCTCTTC	*OmTPS1*
pEAQ_Infusion _OmTPS1-R	AGTTAAAGGCCTCGATCACATAACCGGCCCAAACA	
pEAQ_Infusion _OmTPS3-F	TTCTGCCCAAATTCGATGGCGTCGCTCGCGTTCAC	*OmTPS3*
pEAQ_Infusion _OmTPS3-R	AGTTAAAGGCCTCGACTACAAGGATTCATAAATTAAGGA	
pEAQ_Infusion _OmTPS4-F	TTCTGCCCAAATTCGCGAATGTCACTCGCCTTCAGC	*OmTPS4*
pEAQ_Infusion _OmTPS4-R	AGTTAAAGGCCTCGAGCTAGGAGCTTAGGGTTTTCAT	
pEAQ_Infusion _OmTPS5-F	TTCTGCCCAAATTCGATGGTATCTGCATGTCTAAA	*OmTPS5*
pEAQ_Infusion _OmTPS5-R	AGTTAAAGGCCTCGATCATGAAGGAATTGAAGGAA	
pEAQ_Infusion _MsTPS1-F	TTCTGCCCAAATTCGATGAGTTCCATTCGAAATTTAAGT	*MsTPS1*
pEAQ_Infusion _MsTPS1-R	AGTTAAAGGCCTCGATCACTTGAGAGGCTCAAACATCAT	
pEAQ_Infusion _PcTPS1-F	TTCTGCCCAAATTCGATGTCATTTGCTTCTCAATCAC	*PcTPS1*
pEAQ_Infusion _PcTPS1-R	AGTTAAAGGCCTCGACTACATCACCCTCTCAAACAATAC	
pEAQ_Infusion _HsTPS1-F	TTCTGCCCAAATTCGATGGCGTATATGATATCTATTTCAAATCTC	*HsTPS1*
pEAQ_Infusion _HsTPS1-R	AGTTAAAGGCCTCGATCAGACAATGGGCTCAAATAGAAC	
pEAQ_Infusion _EpTPS8-F	TTCTGCCCAAATTCGATGCAAGTCTCTCTCTCCCTCA	*EpTPS8*
pEAQ_Infusion _EpTPS8-R	AGTTAAAGGCCTCGATTATGAAGTTAAAAGGAGTGGTT	
pEAQ_Infusion _PVTPS1-F	TTCTGCCCAAATTCGCGAATGTCACTCACTTTCAACG	*PVTPS1*
pEAQ_Infusion _PVTPS1-R	AGTTAAAGGCCTCGAGCTAGTTTCTCACAGAAGTCAA	
**Cloning of diTPS genes into pET-28 b (+) for E. coli expression**		

pET28_CfTPS1-F	AGGAGATATACCATGGCCGAGATTCGAGTTGCCAC	*CfTPS1*
pET28_CfTPS1-R	GGTGGTGGTGCTCGAAGGCGACTGGTTCGAAAAGTAC	
pET28_SsSS-F	AGGAGATATACCATGGATTTCATGGCGAAAATGAAAGAGA	*SsSS*
pET28_SsSS-R	GGTGGTGGTGCTCGAAAAAGACAAAGGATTTCATAT	
pET28_CfTPS2-F	AGGAGATATACCATGCAAATTCGTGGAAAGCAAAGATCAC	*CfTPS2*
pET28_CfTPS2-R	GGTGGTGGTGCTCGAAGACCACTGGTTCAAATAGAACT	
pET28_CfTPS3-F	AGGAGATATACCATGTCTAAATCATCTGCAGCTGT	*CfTPS3*
pET28_CfTPS3-R	GGTGGTGGTGCTCGAAGTTGCTGACACAACTCATT	
pET28_OmTPS3-F	AGGAGATATACCATGACCGTCAAATGCTAC	*OmTPS3*
pET28_OmTPS3-R	GGTGGTGGTGCTCGAACAAGGATTCATAAATTAAG	
pET28_OmTPS5-F	AGGAGATATACCATGACTGTCAAGTGCAGC	*OmTPS5*
pET28_OmTPS5-R	GGTGGTGGTGCTCGAATGAAGGAATTGAAG	
pET28_PcTPS1-F	AGGAGATATACCATGTTTATGCCCACTTCCATTAAATGTA	*PcTPS1*
pET28_PcTPS1-R	GGTGGTGGTGCTCGAACATCACCCTCTCAAACAATACTTTGG	
pET28_HsTPS1-F	AGGAGATATACCATGGTAGCAAAAGTGATCGAGAGCCGAGTTA	*HsTPS1*
pET28_HsTPS1-R	GGTGGTGGTGCTCGAAGACAATGGGCTCAAATAGAACTTTAAAT	

Table S2. List of synthetic oligonucleotides used in this study.

	CfTPS1 [**31**]		CfTPS2 [**10**]		LlTPS1 [**5**]		ZmAN2 [**16**]		HsTPS1 [**21**]		PcTPS1 [**25**]		ArTPS2 [**38**]		OmTPS1 [**31**]	
	products	figure	products	figure	products	figure	products	figure	products	figure	products	figure	products	figure	products	figure
ArTPS3	**32**	S-3A	**8**	S-4B	**1**, **2**, **3**	S-5A	np	S-6B	-		-		-	-	-	
LlTPS4	**27**	S-3A	**8**	S-4A	**1**, **2**, **3**	S-5B	np	S-6A	-		-		-		-	
MsTPS1	**27**	S-3A	**8**	S-4C	**3**	S-5A	np	S-6A	-		-		np	S-8A	-	
NmTPS2	np	S-3D	np	S-4D	np	S-5D	**19**	S-6A	-		-		np	S-8A	-	
OmTPS3	**34**	S-3A	**11**	S-4D,E	**1**, **2**	S-5A	np	S-6A	**24**	S-8B	-		np	S-8A	**34**	S-3C
OmTPS4	**33**	S-3A	**8**	S-4C	**1**, **2**, **3**, **4**	S-5D	**20**	S-6A	-		-		-		**33**	S-3C
OmTPS5	**29**	S-3A	**8**	S-4A	**1**, **2**, **3**	S-5A	np	S-6A	-		-		np	S-8A	**29**	S-3C
PaTPS3	**32**	S-3B	**8**	S-4B	**1**, **2**, **3**	S-5C	np	S-6B	-		-		-		-	
PvTPS1	**32**	S-3B	**8**	S-4B	**1**, **2**, **3**	S-5C	np	S-6B	-		-		-		-	
SoTPS1	**32**	S-3B	**8**	S-4B	**1**, **2**, **3**	S-5D	np	S-6B	-		-		-		-	
CfTPS3	**32**		**8**		**1**, **2**, **3**		np		**22**	S-8B	np	S-8C	np		**32**	S-7D
SsSS	**33**		-		**4**		**20**		**23**	S-8B	**26**	S-8C	**37**	S-8A	-	

Table S3. Index of class I diTPS functional assays by N. benthamiana transient expression. Bold umbers refer to compound numbers; “np” indicates that the combination was tested but no product was detected; “-” indicates that the combination was not tested. Blue genes are new to this study.

	Product	Figure
ArTPS1	Copalyl-PP [**31**]	S-7A
CfTPS16	Copalyl-PP [**31**]	S-7B
NmTPS1	Copalyl-PP [**31**]	S-7C
OmTPS1	Copalyl-PP [**31**]	S-7D
PaTPS1	Copalyl-PP [**31**]	S-7A
ArTPS2	Neo-cleroda-4(18),13E-dienyl-PP [**38**]	S-8A
HsTPS1	Labda-7,13*E* -dienyl-PP [**21**]	S-8B
LlTPS1	Peregrinol-PP [**7**]	S-5B
PcTPS1	*Ent* -labda-8,13*E* -dienyl-PP [**25**]	S-8C

Table S4. Index of class II diTPS functional assays by *N. benthamiana* transient expression. Blue genes are new to this study.

Class II	Class I	Product	Figure
CfTPS1 [**31**]	OmTPS3	trans-biformene [**34**]	S-9C
CfTPS2 [**10**]	OmTPS3	trans-abienol [**11**]	S-9D
HsTPS1 [**21**]	OmTPS3	[**24**]	S-9B
CfTPS1 [**31**]	OmTPS5	palustradiene [**29**]	S-9E
ArTPS2 [**38**]	SsSS	[**37**]	5A
HsTPS1 [**21**]	SsSS	[**23**]	S-9A
PcTPS1 [**25**]	SsSS	[**26**]	5B

Table S5. Index of *in-vitro* assays. Blue genes are new to this study.

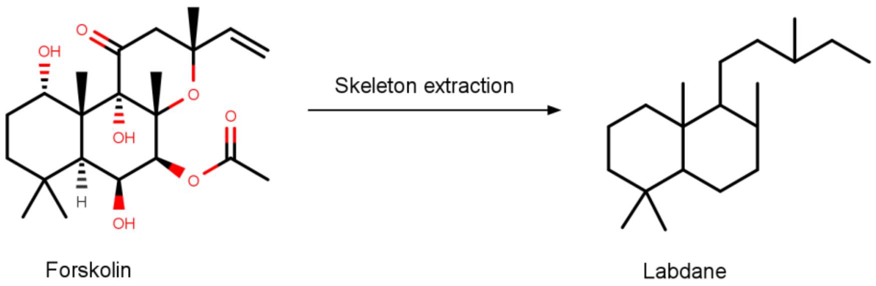

Figure S1. An example of skeleton extraction. By deleting all heteroatoms, desaturation, and stereochemistry, the labdane skeleton is extracted from the forskolin structure.

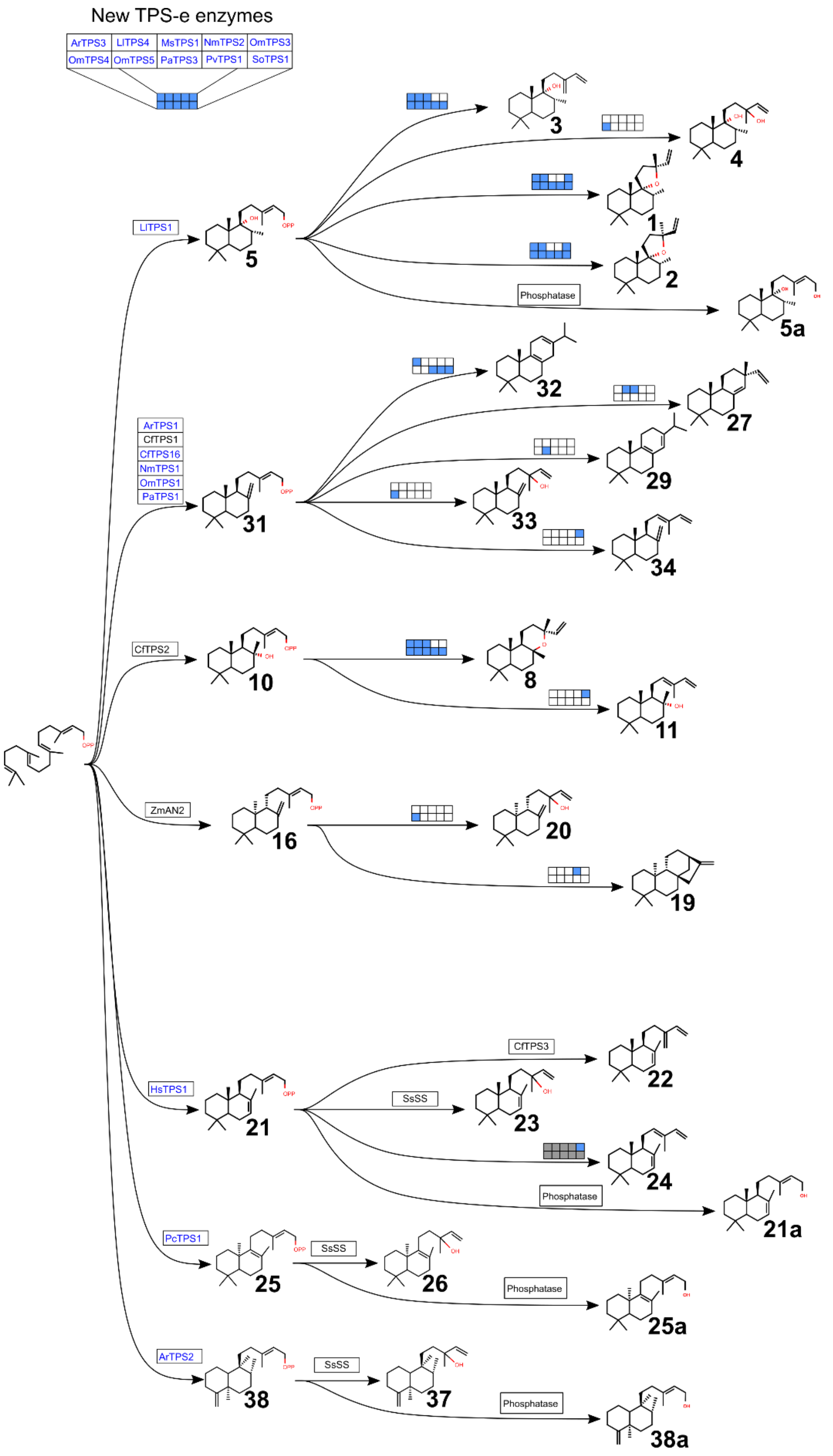

Figure S2. Newly characterized enzyme activities. Blue genes are newly characterized. Blue square: TPS-e from that position on the key catalyzes the shown transformation. White square: corresponding TPS-e does not catalyze the shown activity. Grey square: corresponding TPS-e was not tested on the substrate.

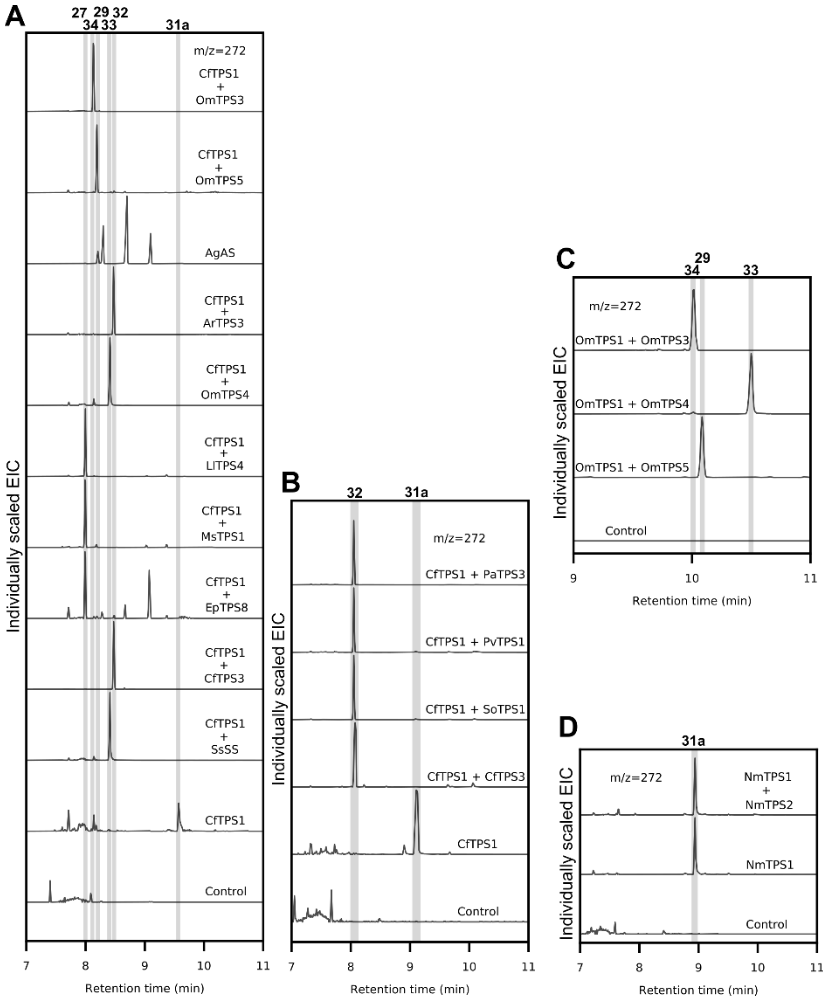

Figure S3. GC-MS chromatograms of hexane extracts from *N. benthamiana* transiently expressing (+)-CPP-producing class II diTPSs along with new class I diTPSs, and reference combinations. AgAS, *Abies grandis* abietadiene synthase; EpTPS8, *Euphorbia peplus* TPS8.

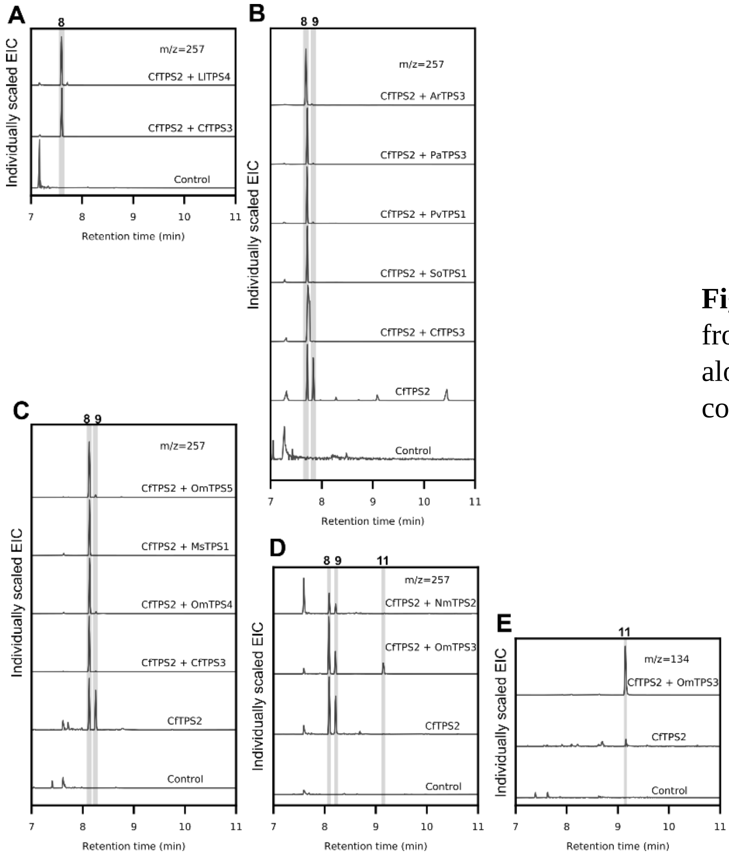

Figure S4. GC-MS chromatograms of hexane extracts from *N. benthamiana* transiently expressing CfTPS2 along with new class I diTPSs, and reference combinations.

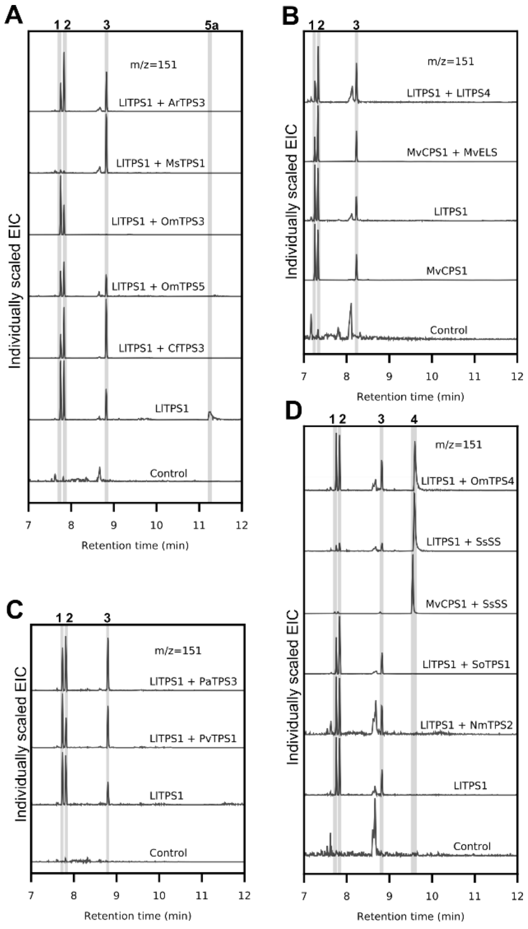

Figure S5. GC-MS chromatograms of hexane extracts from *N. benthamiana* transiently expressing LlTPS1 along with new class I diTPSs, and reference combinations.

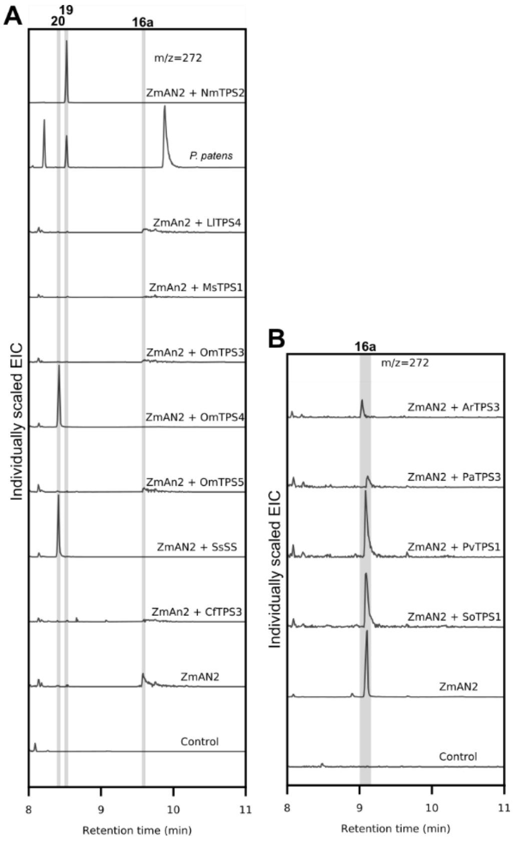

Figure S6. GC-MS chromatograms of hexane extracts from *N. benthamiana* transiently expressing ZmAN2 along with new class I diTPSs, and reference combinations.

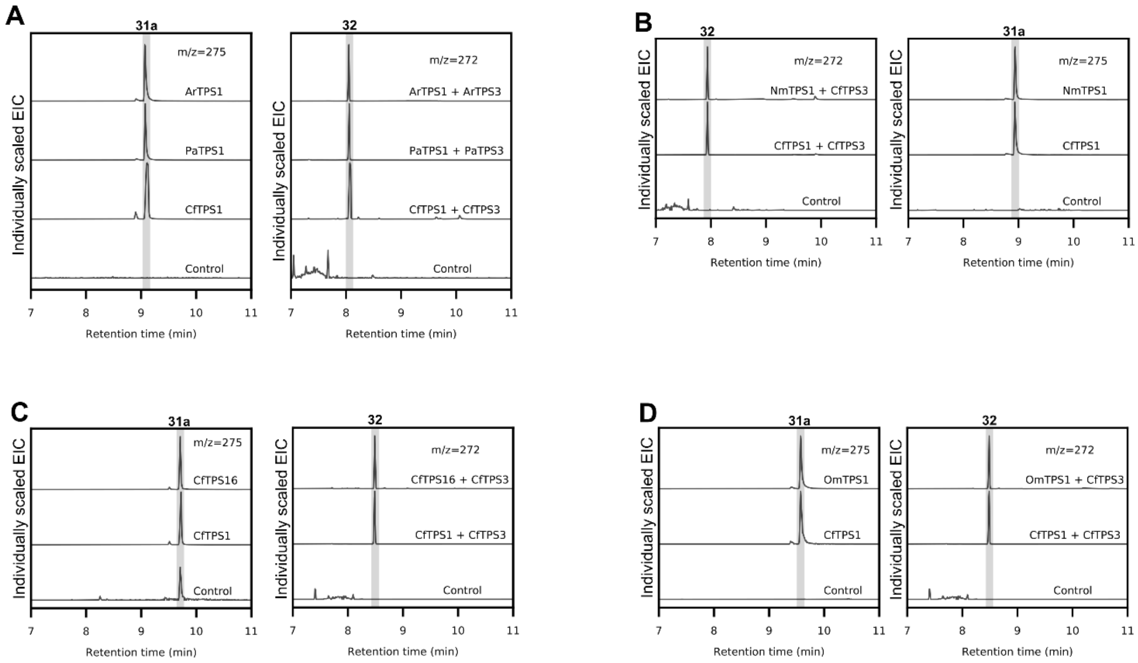

Figure S7. GC-MS chromatograms of hexane extracts from *N. benthamiana* transiently expressing new (+)-CPP synthases along with reference combinations.

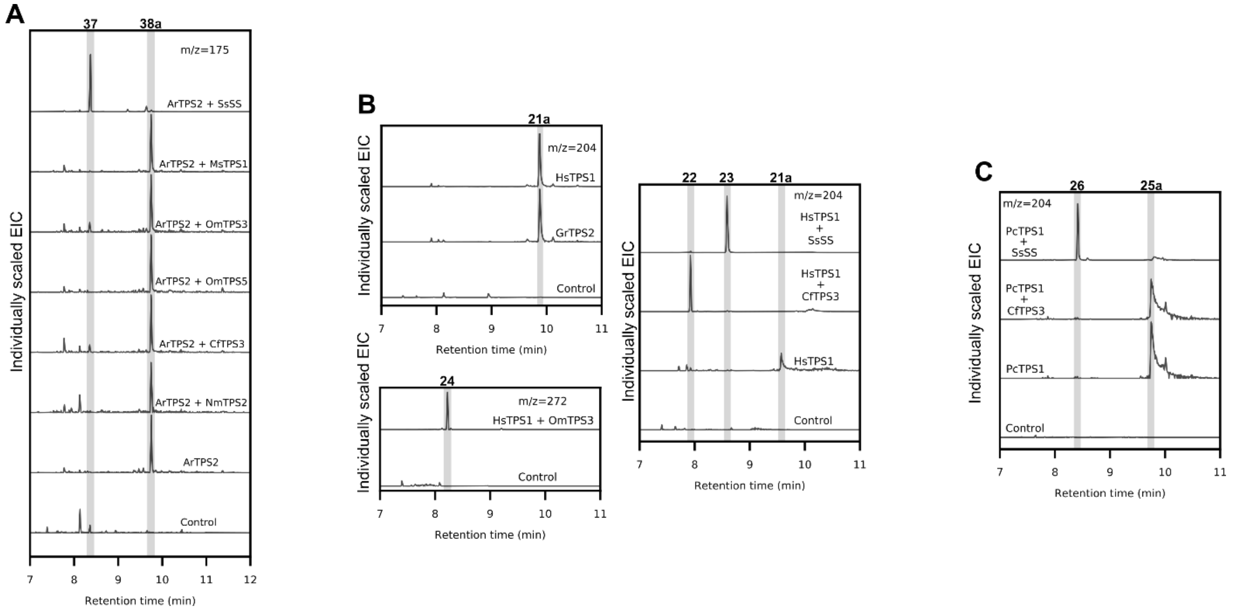

Figure S8. GC-MS chromatograms of hexane extracts from *N. benthamiana* transiently expressing new class II diTPSs, reference combinations, and combinations with new class I diTPSs.

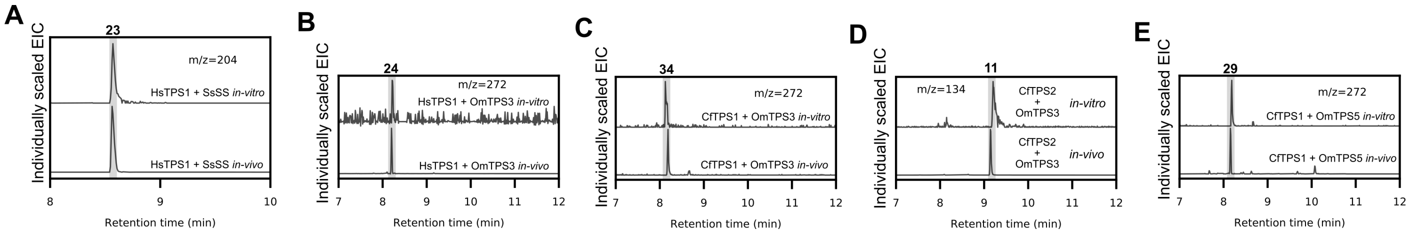

Figure S9. Comparison of GC-MS chromatograms of hexane extracts from *in-vitro* assays of purified diTPSs with extracts from *N. benthamiana* transiently expressing diTPS combinations.

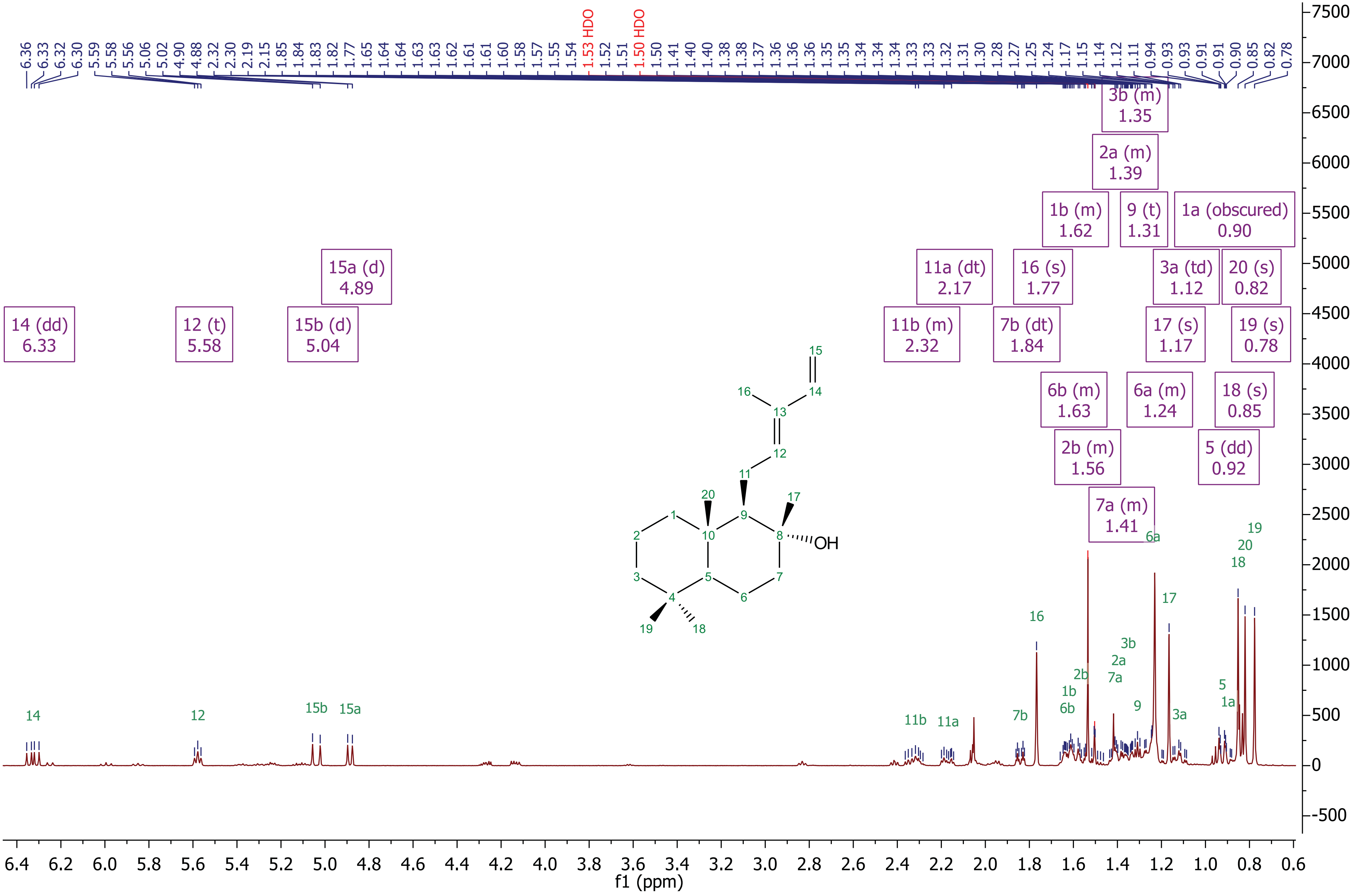

Figure S10-A. ^{1}H NMR of trans-abienol [**11**].

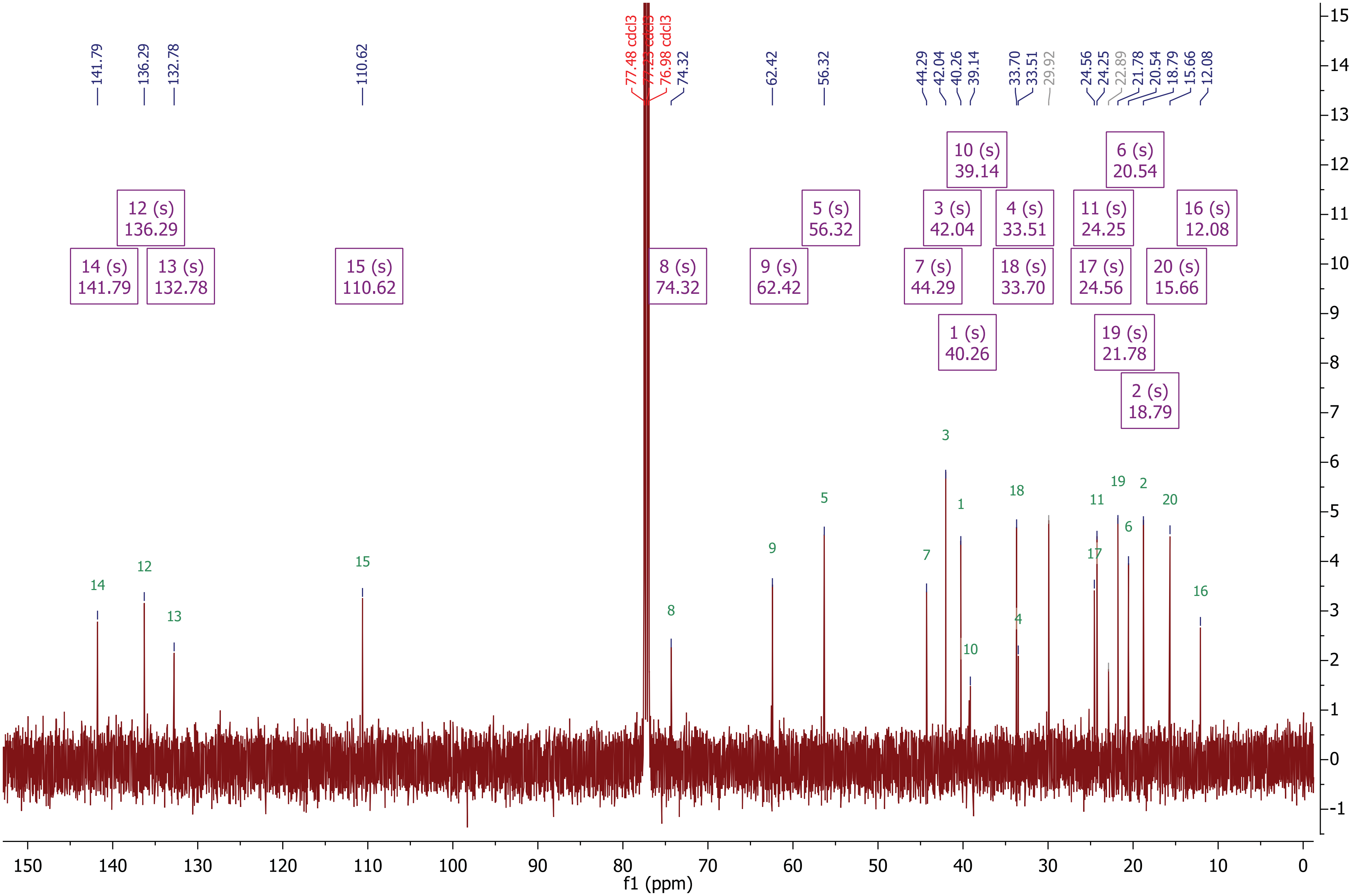

Figure S10-B. ^{13}C NMR of trans-abienol [**11**].

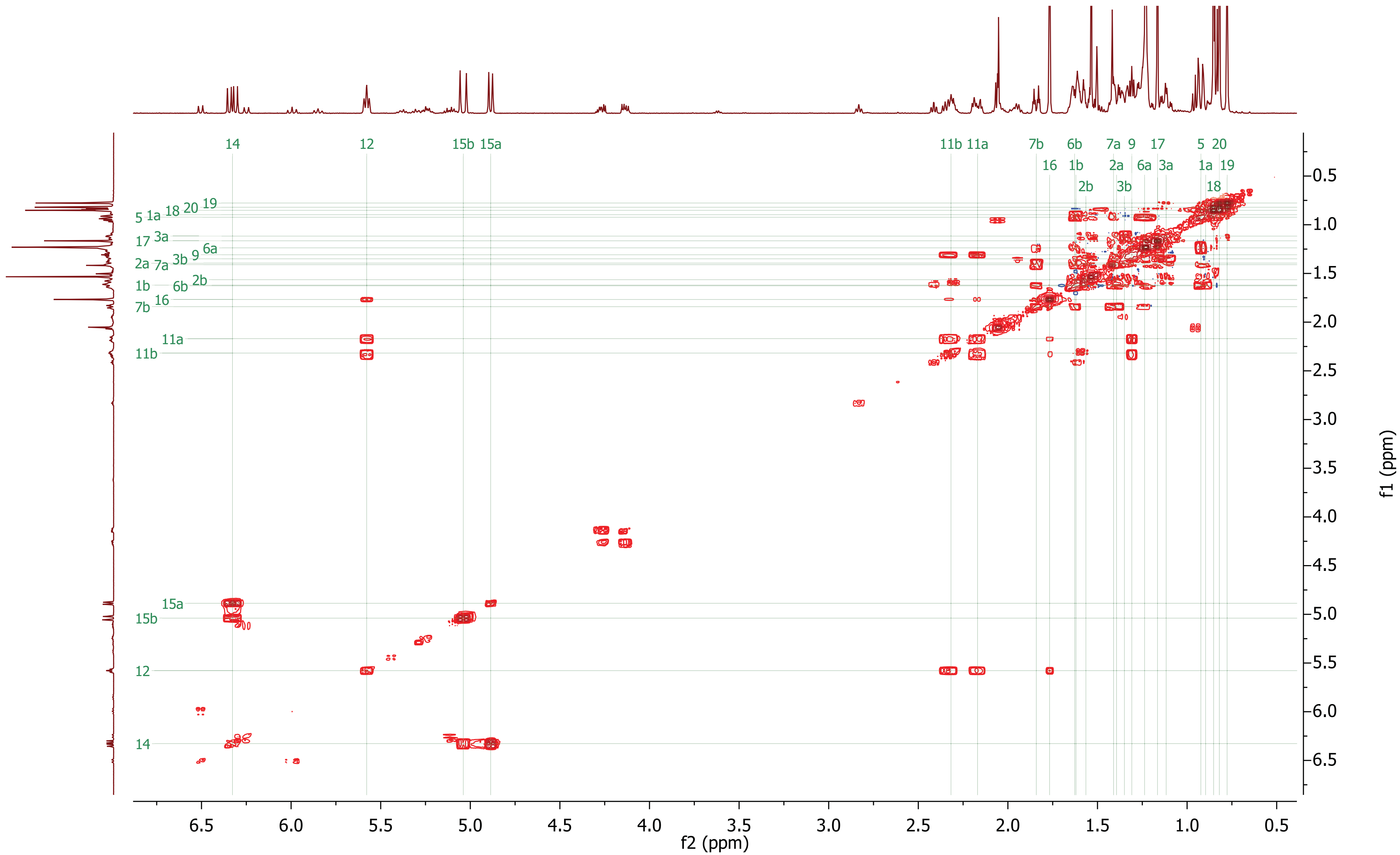

Figure S10-C. ^1H-^1H COSY of trans-abienol [**11**].

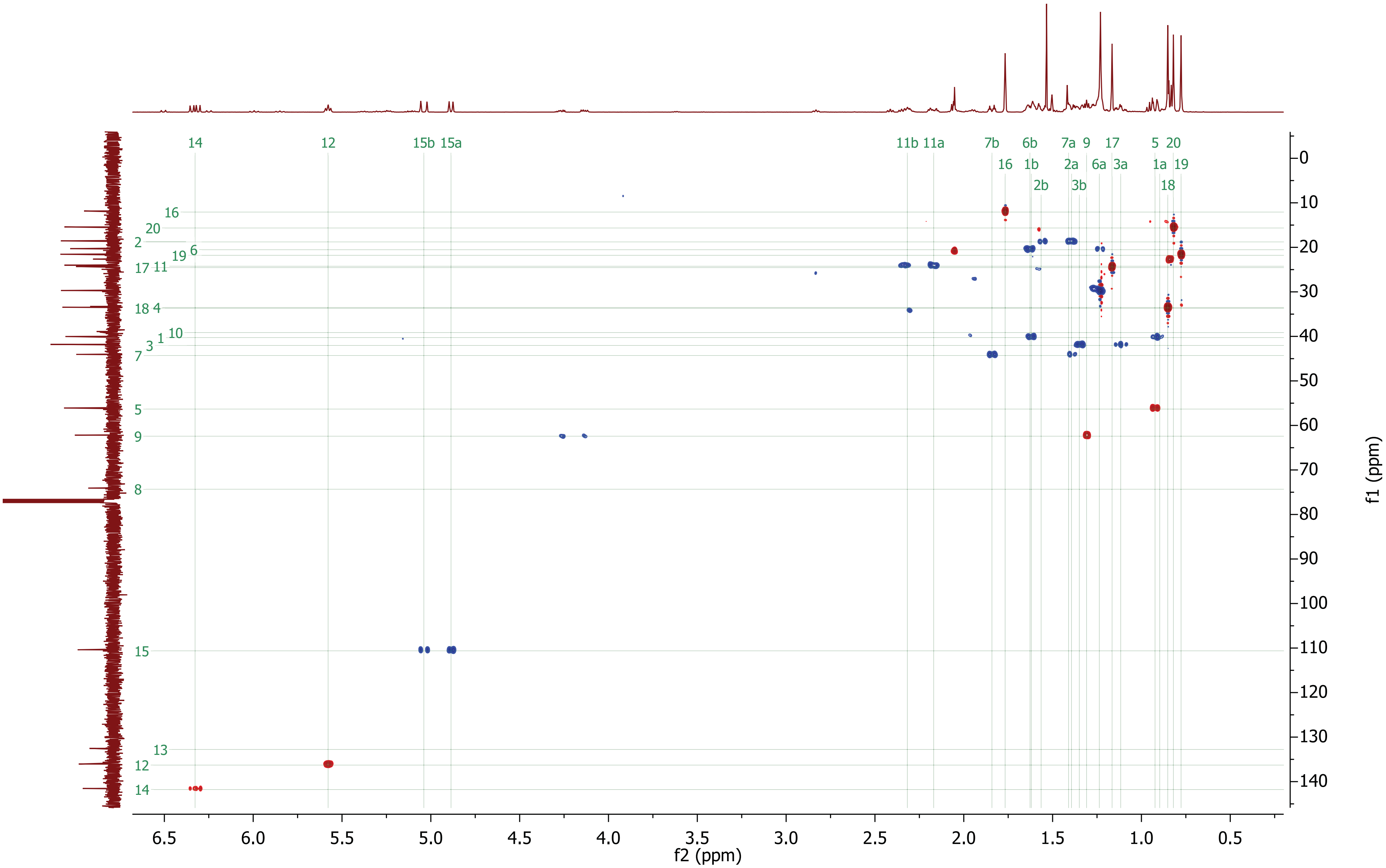

Figure S10-D. ^{1}H-^{13}C HSQC of trans-abienol [**11**].

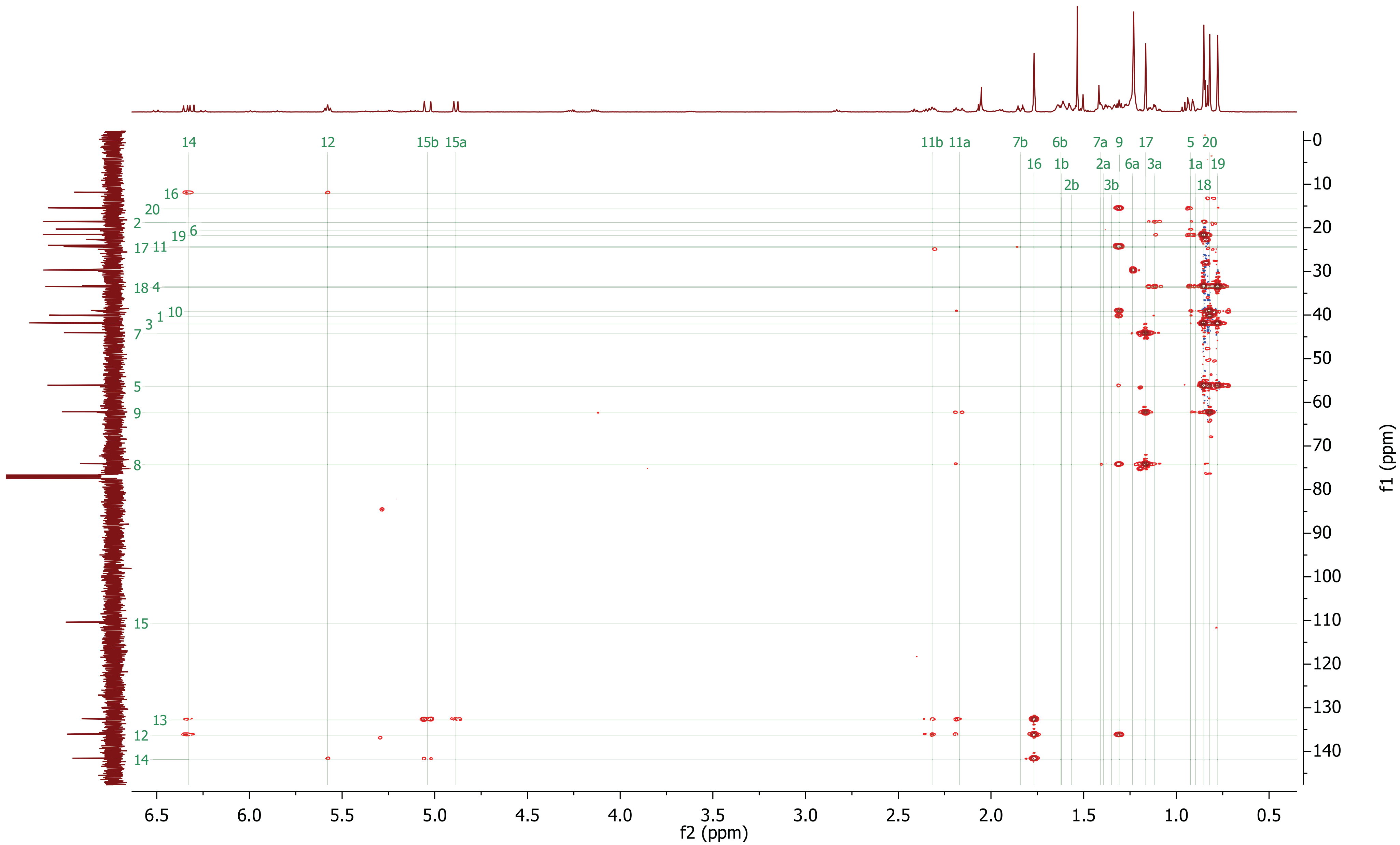

Figure S10-E. ^{1}H-^{13}C HMBC of trans-abienol [**11**].

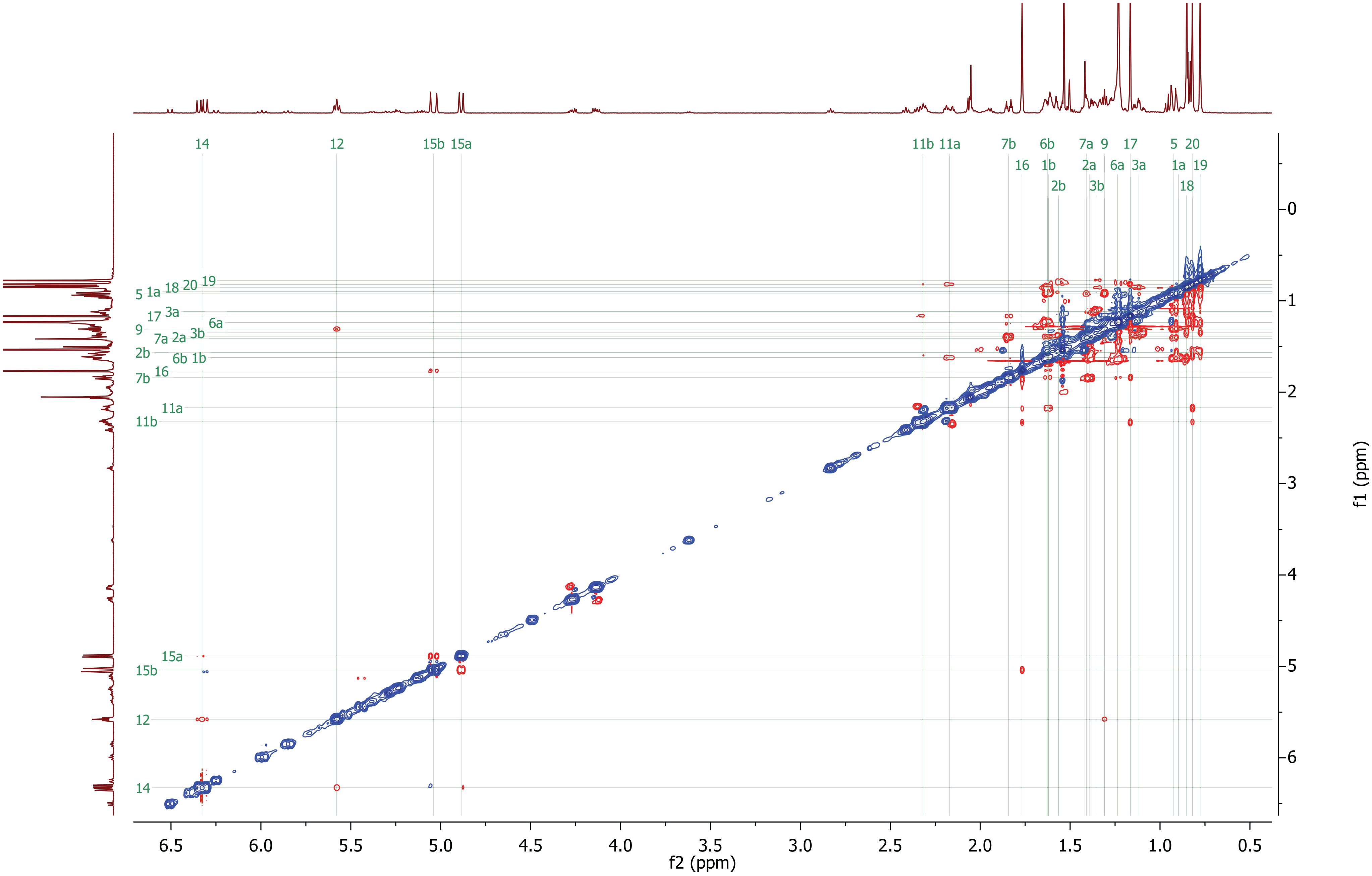

Figure S10-F. ^{1}H NOESY of trans-abienol [**11**].

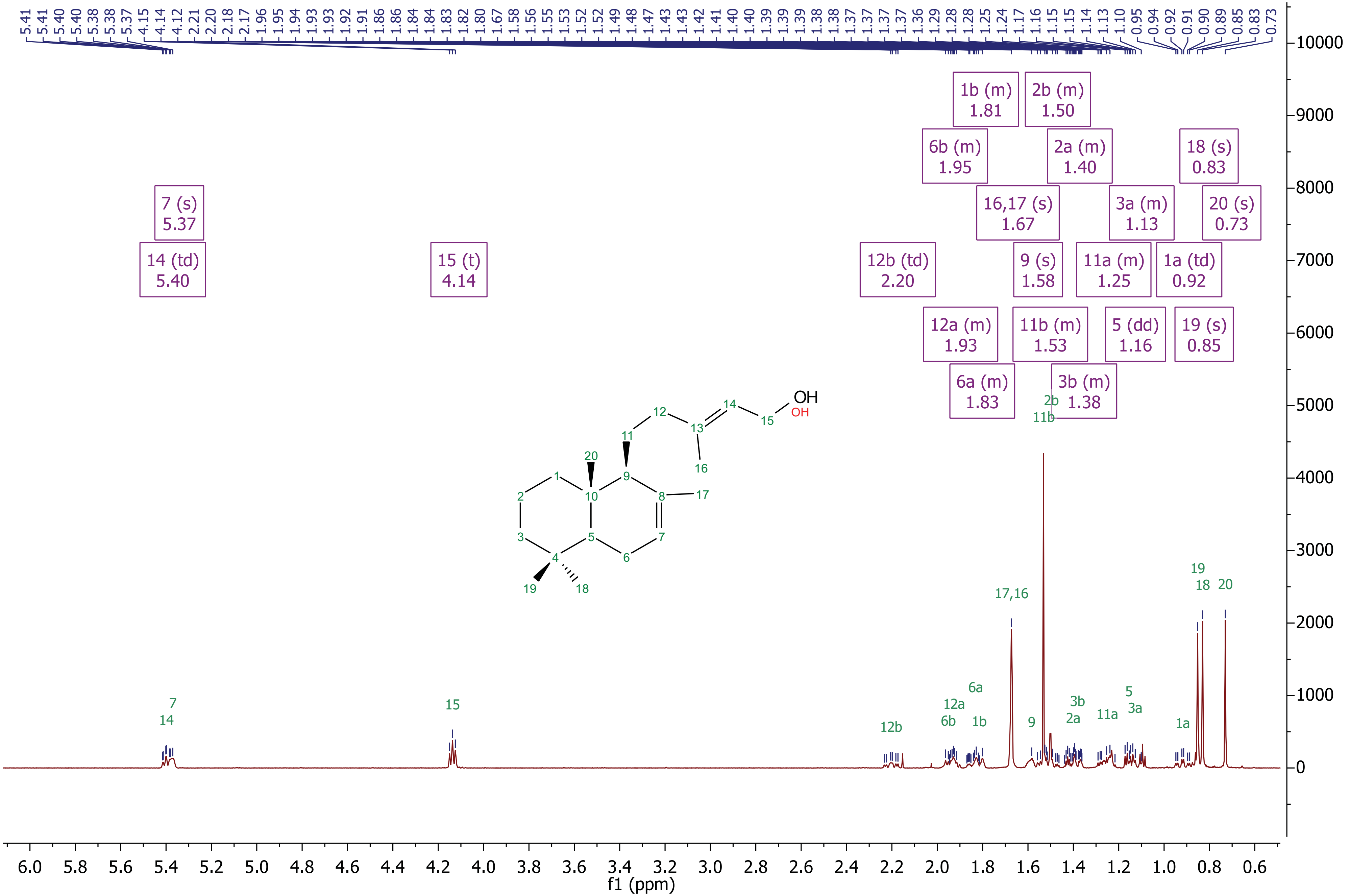

Figure S11-A. ^{1}H NMR of labda-7,13E-dien-15-ol [**21a**].

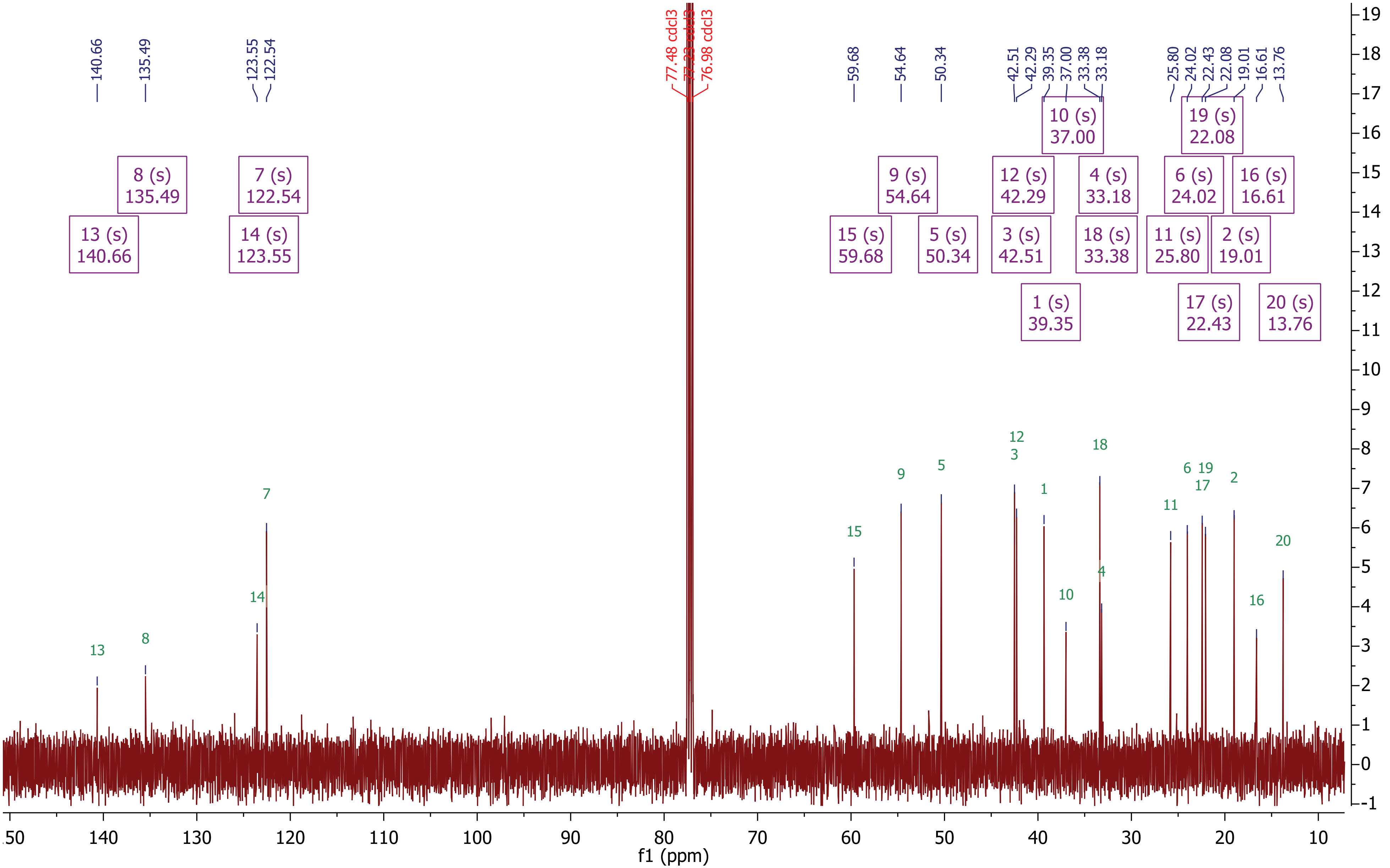

Figure S11-B. ^{13}C NMR of labda-7,13E-dien-15-ol [**21a**].

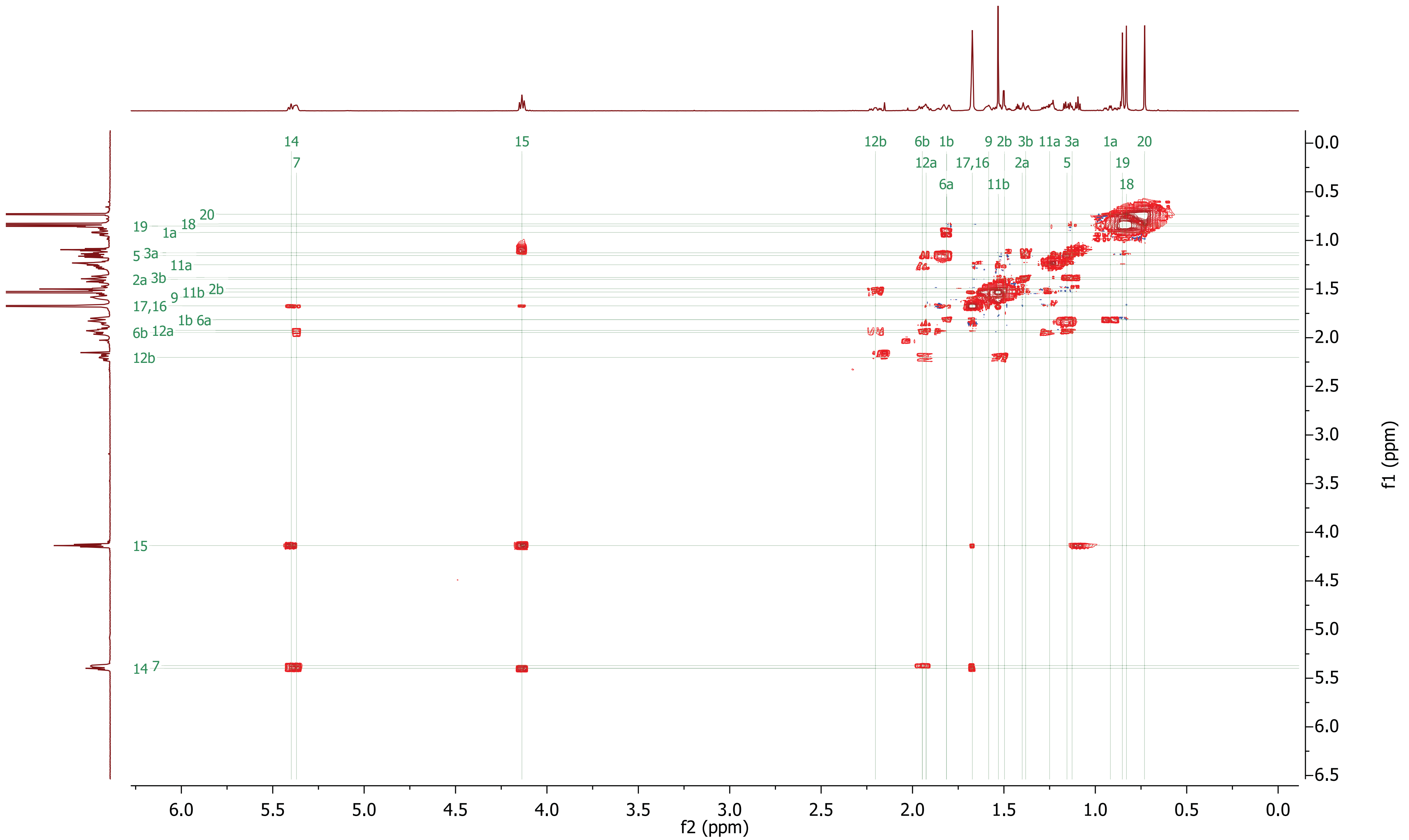

Figure S11-C. ^{1}H-^{1}H COSY of labda-7,13E-dien-15-ol [**21a**].

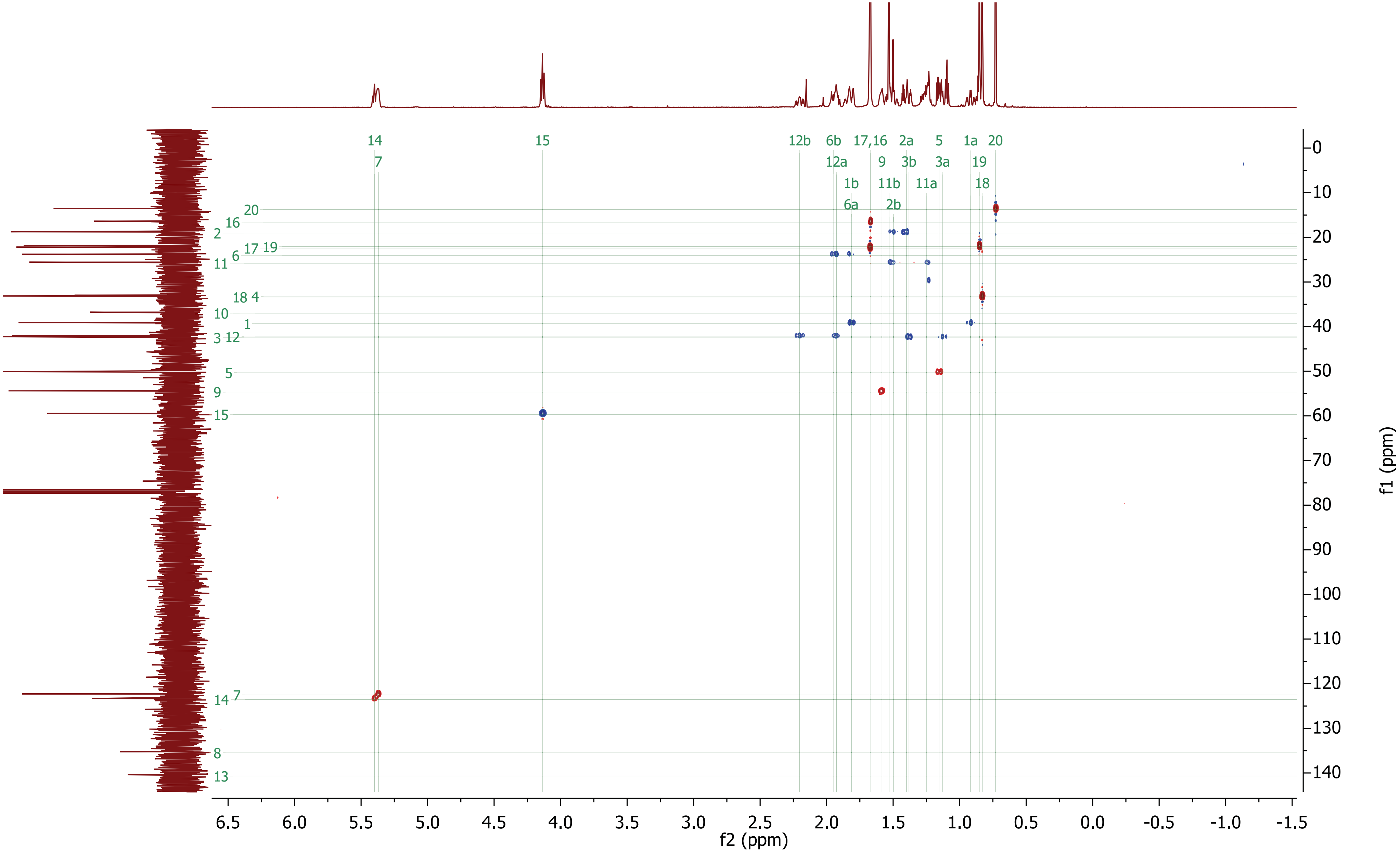

Figure S11-D. ^{1}H-^{13}C HSQC of labda-7,13E-dien-15-ol [**21a**]

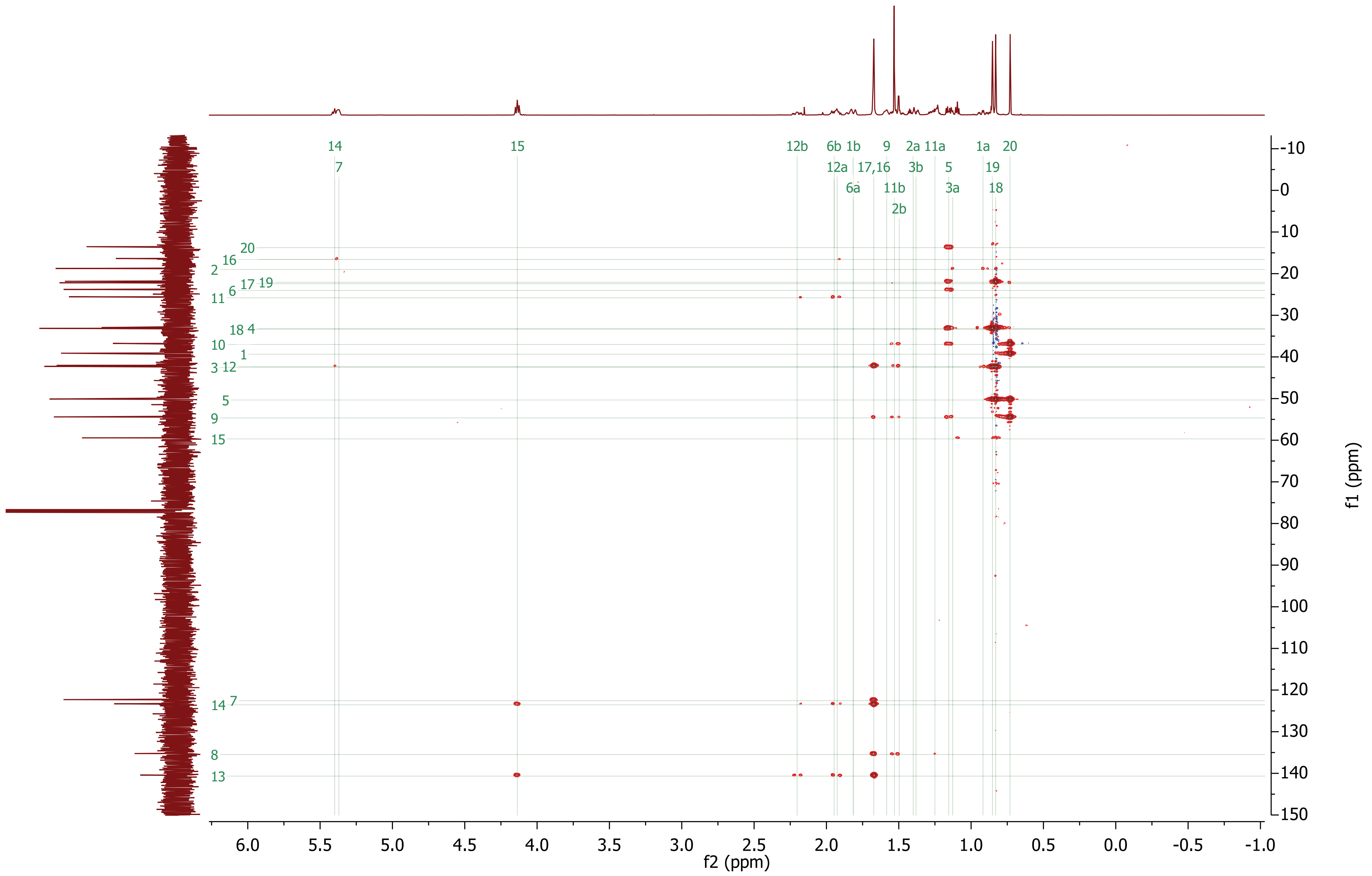

Fig S11-E. ^{1}H-^{13}C HMBC of labda-7,13E-dien-15-ol [**21a**]

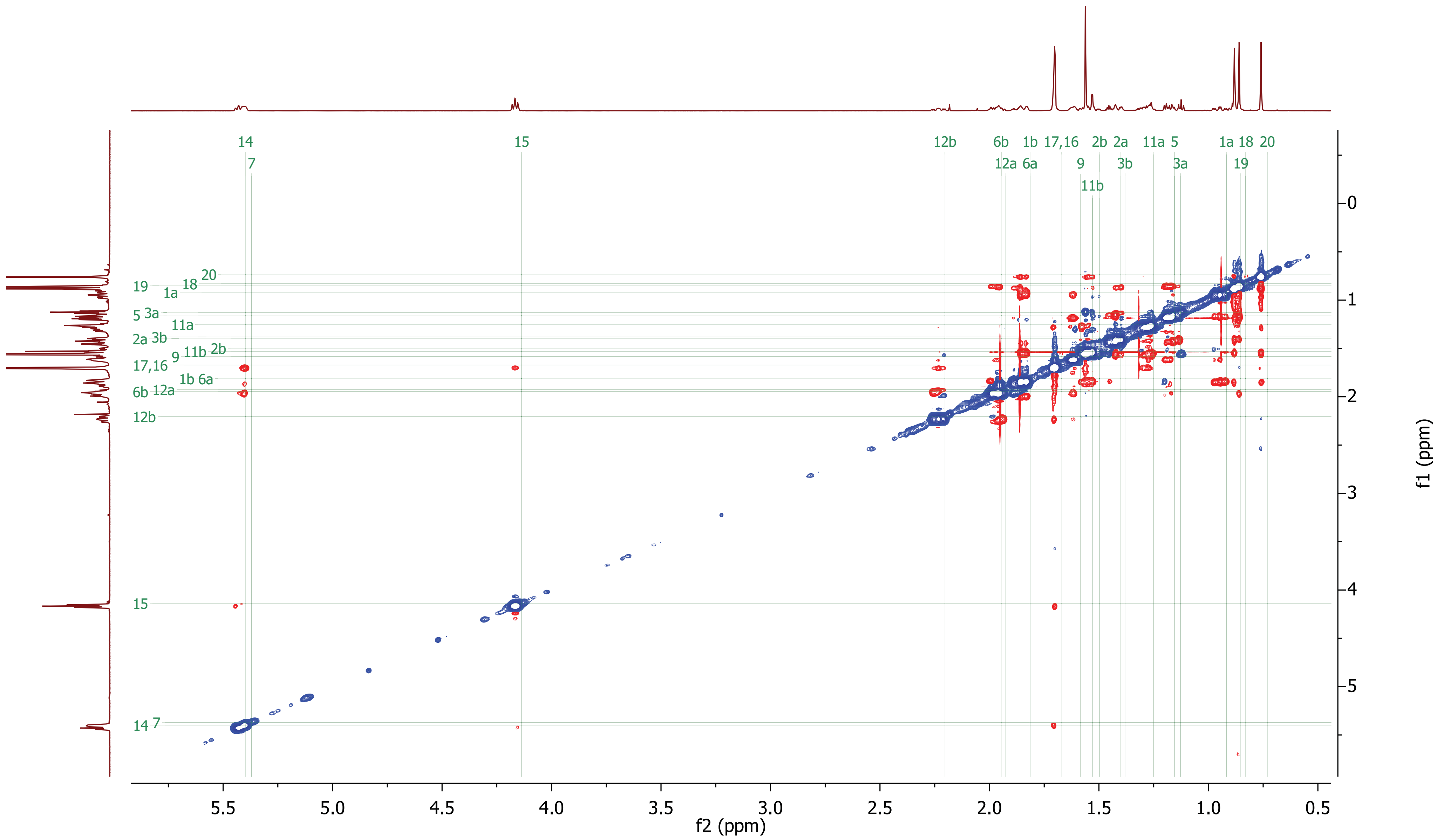

Fig S11-F. ^{1}H NOESY of labda-7,13E-dien-15-ol [**21a**]

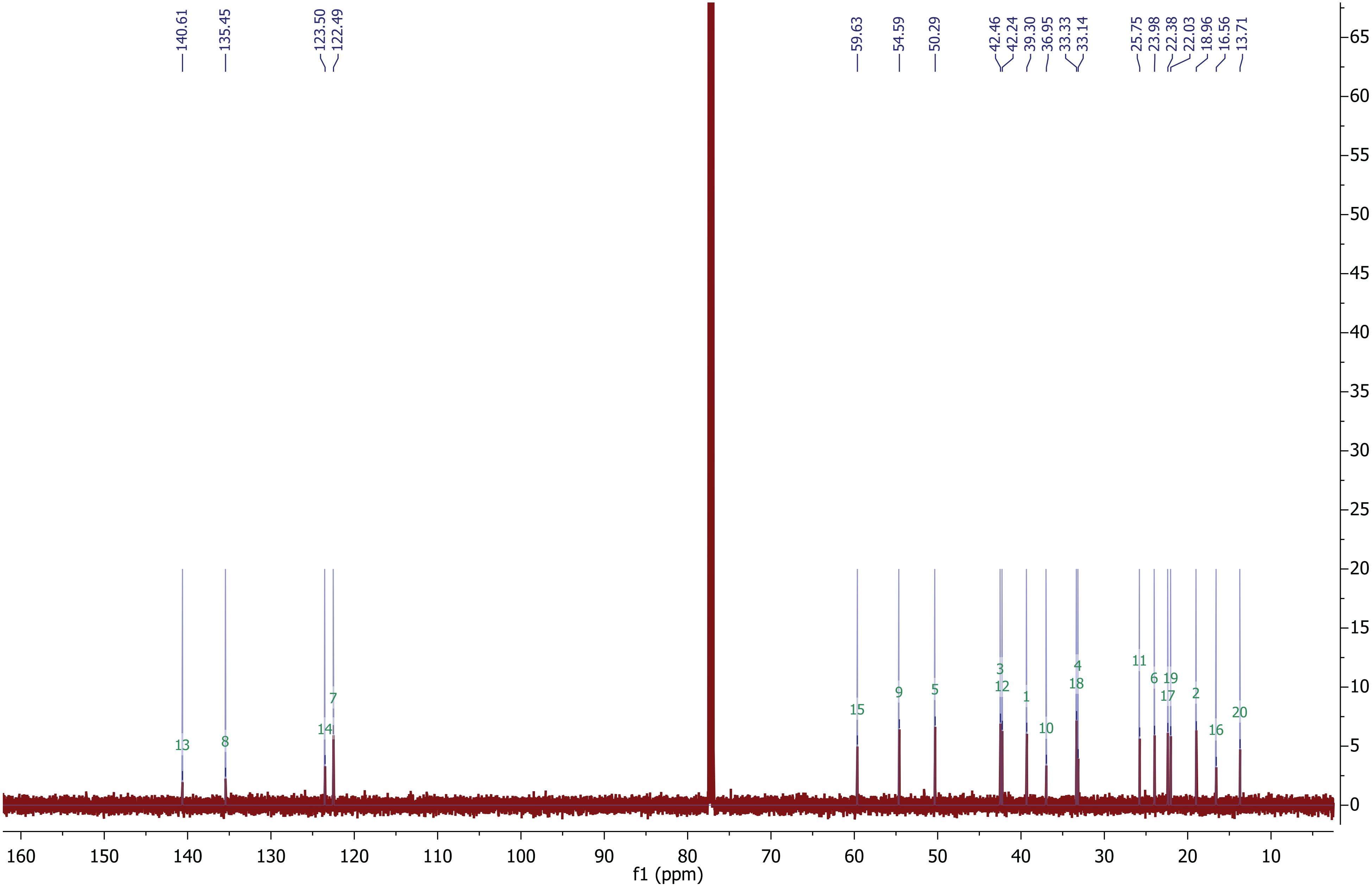

Figure S11-G. Overlay of ^{13}C NMR of labda-7,13E-dien-15-ol [**21a**] (red) with ^{13}C NMR spectrum (blue) reconstructed from shifts reported for the same compound by Mafu et al. (2011) (DOI: 10.1002/cbic.201100336).

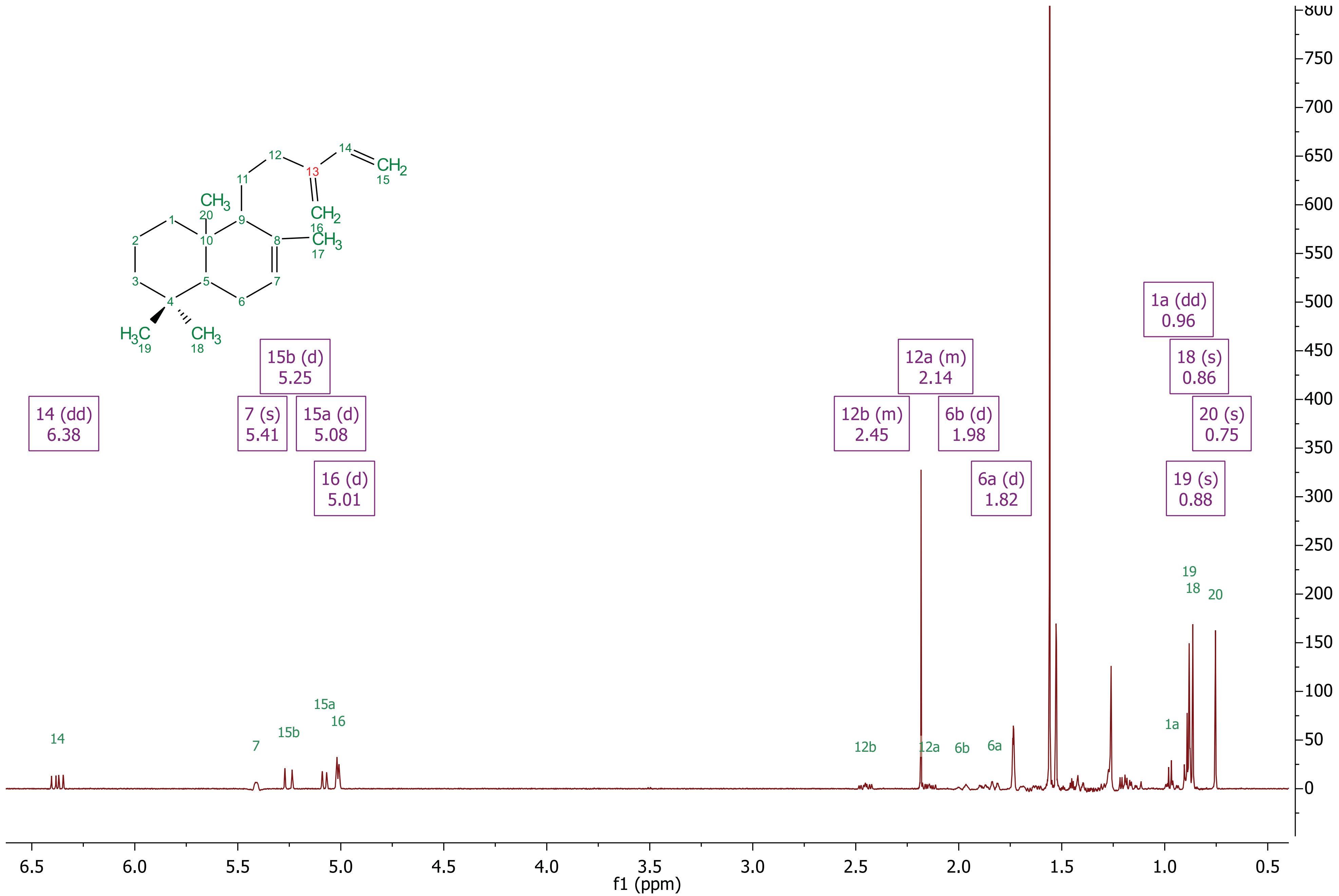

Figure S12-A. ^{1}H NMR of partially purified labda-7,13(16),14-triene [**22**].

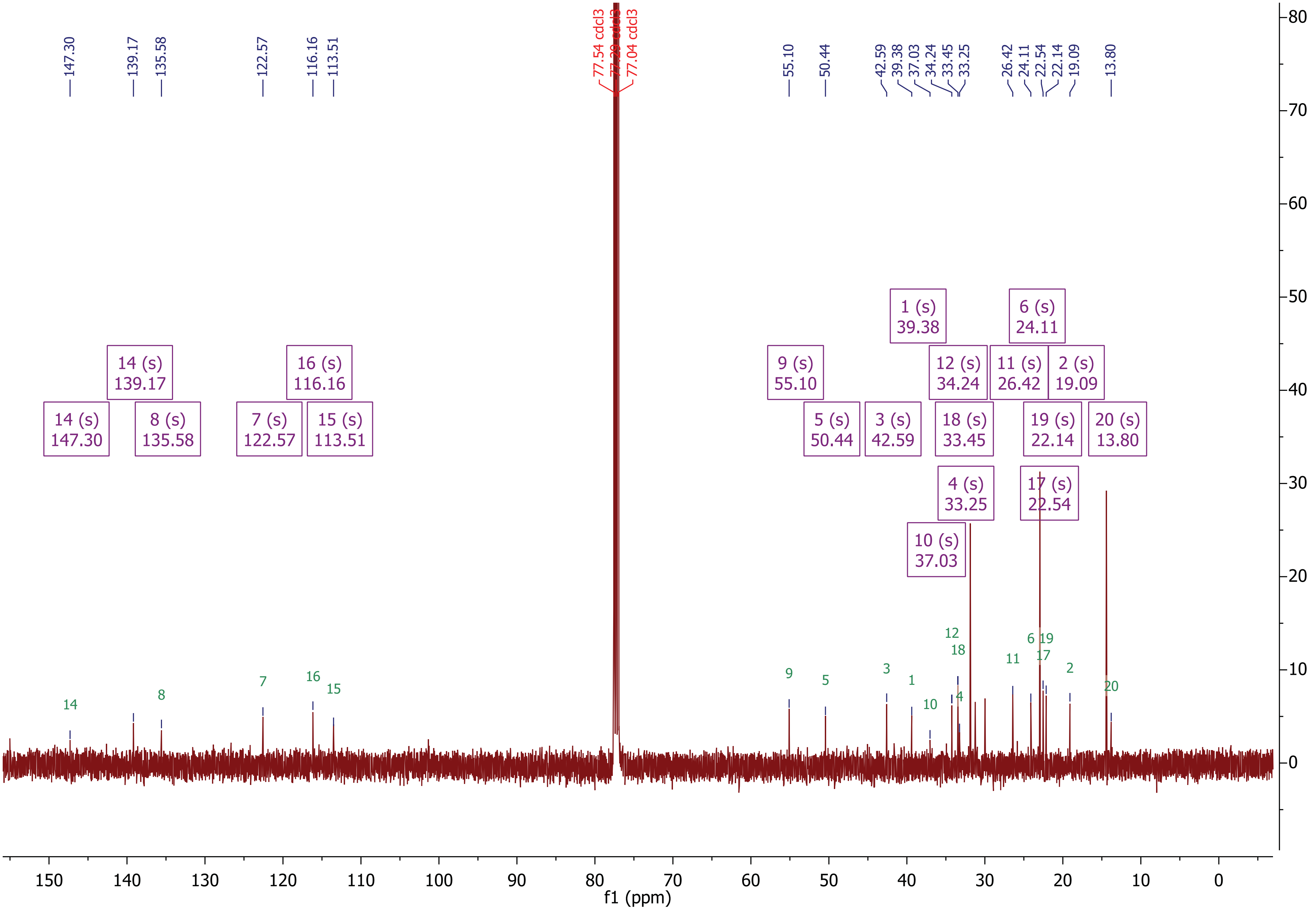

Figure S12-B. ^{13}C NMR of partially purified labda-7,13(16),14-triene [**22**].

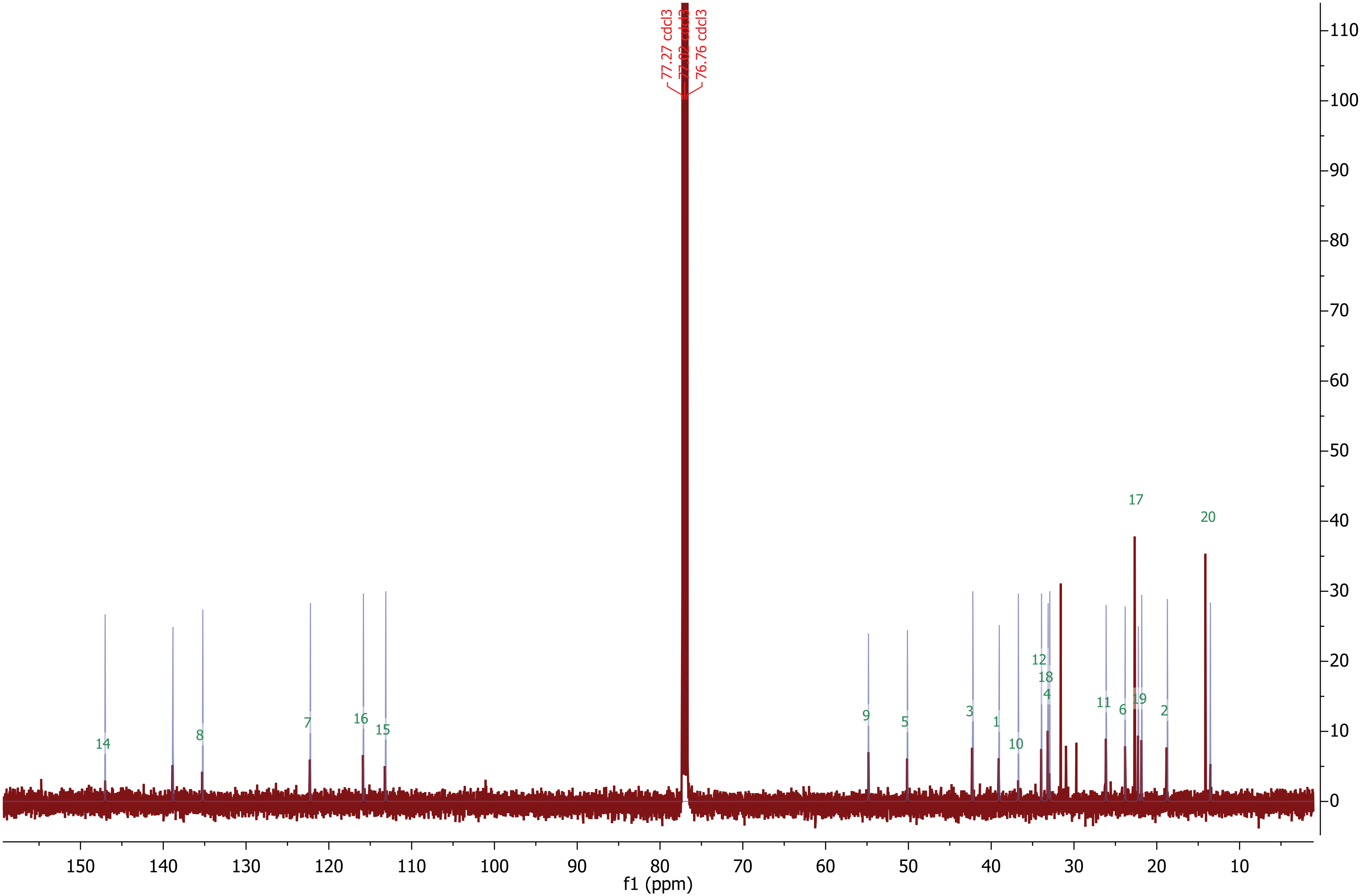

Figure S12-C. Overlay of ^{13}C NMR of partially purified labda-7,13(16),14-triene [**22**] (red) with ^{13}C NMR spectrum (blue) reconstructed from shifts reported for the same compound by Jia et al. (2016) (DOI: 10.1016/j.ymben.2016.04.001).

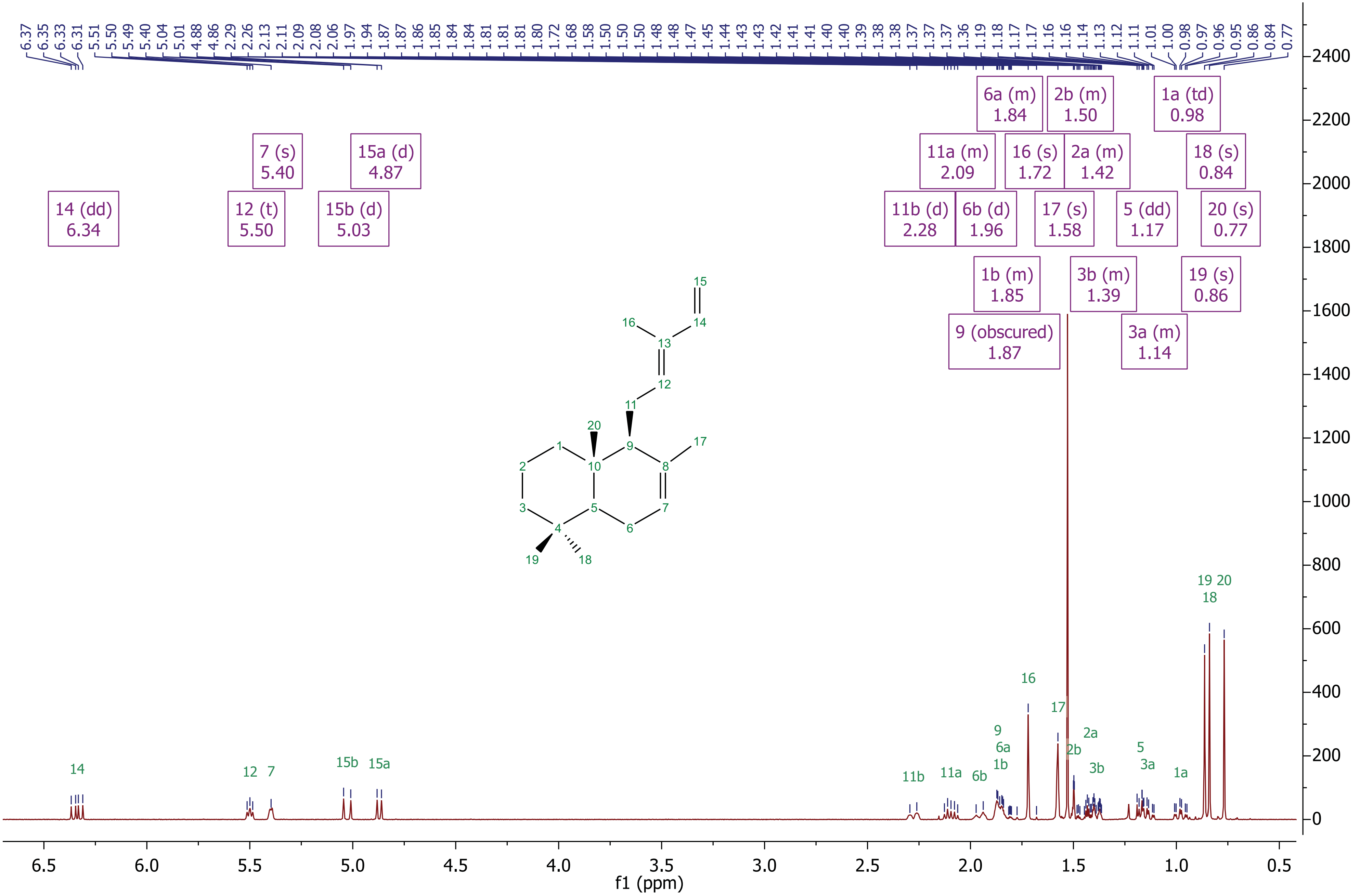

Figure S13-A. ^{1}H NMR of labda-7,12E,14-triene [**24**].

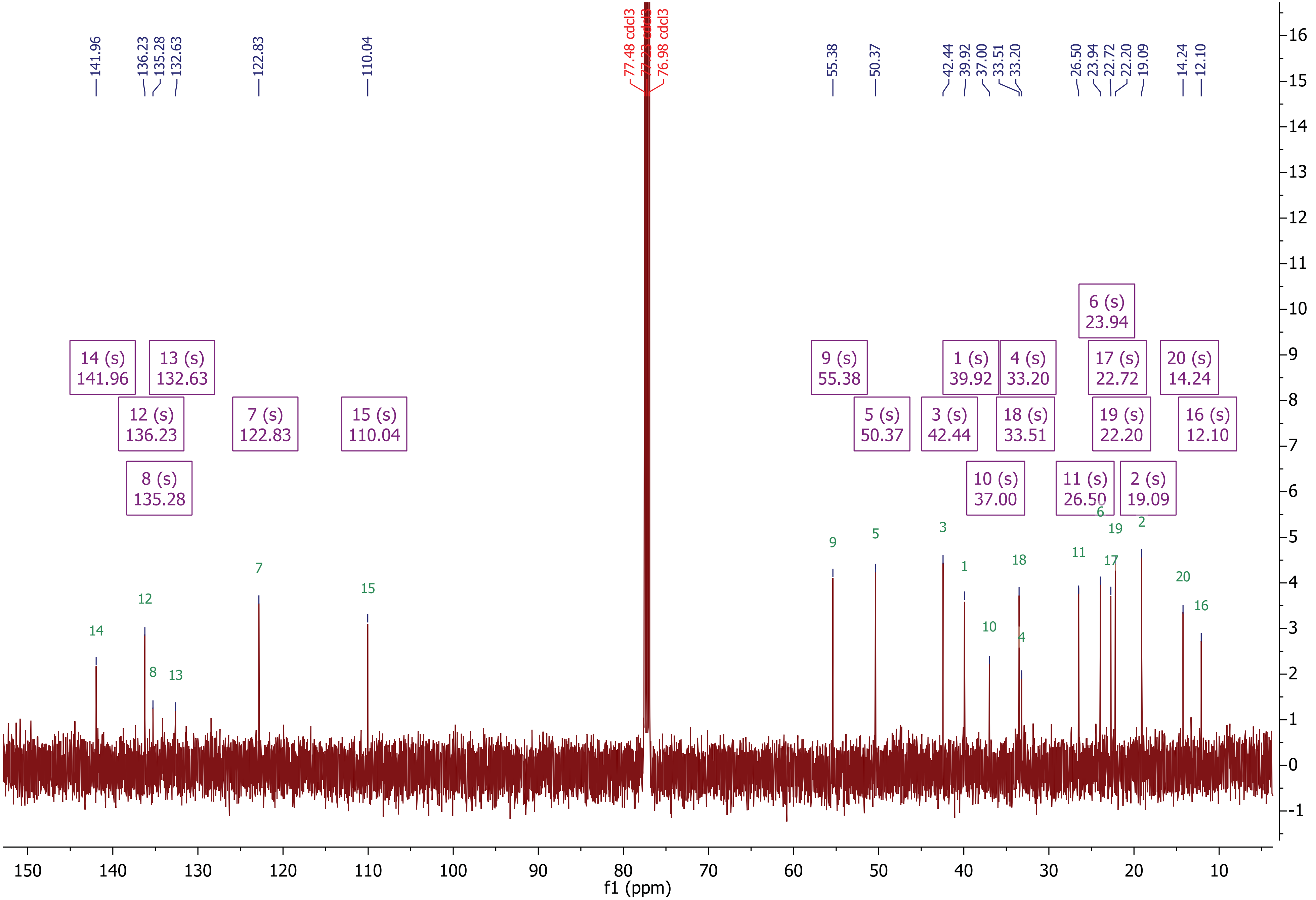

Figure S13-B. ^{13}C NMR of labda-7,12E,14-triene [**24**].

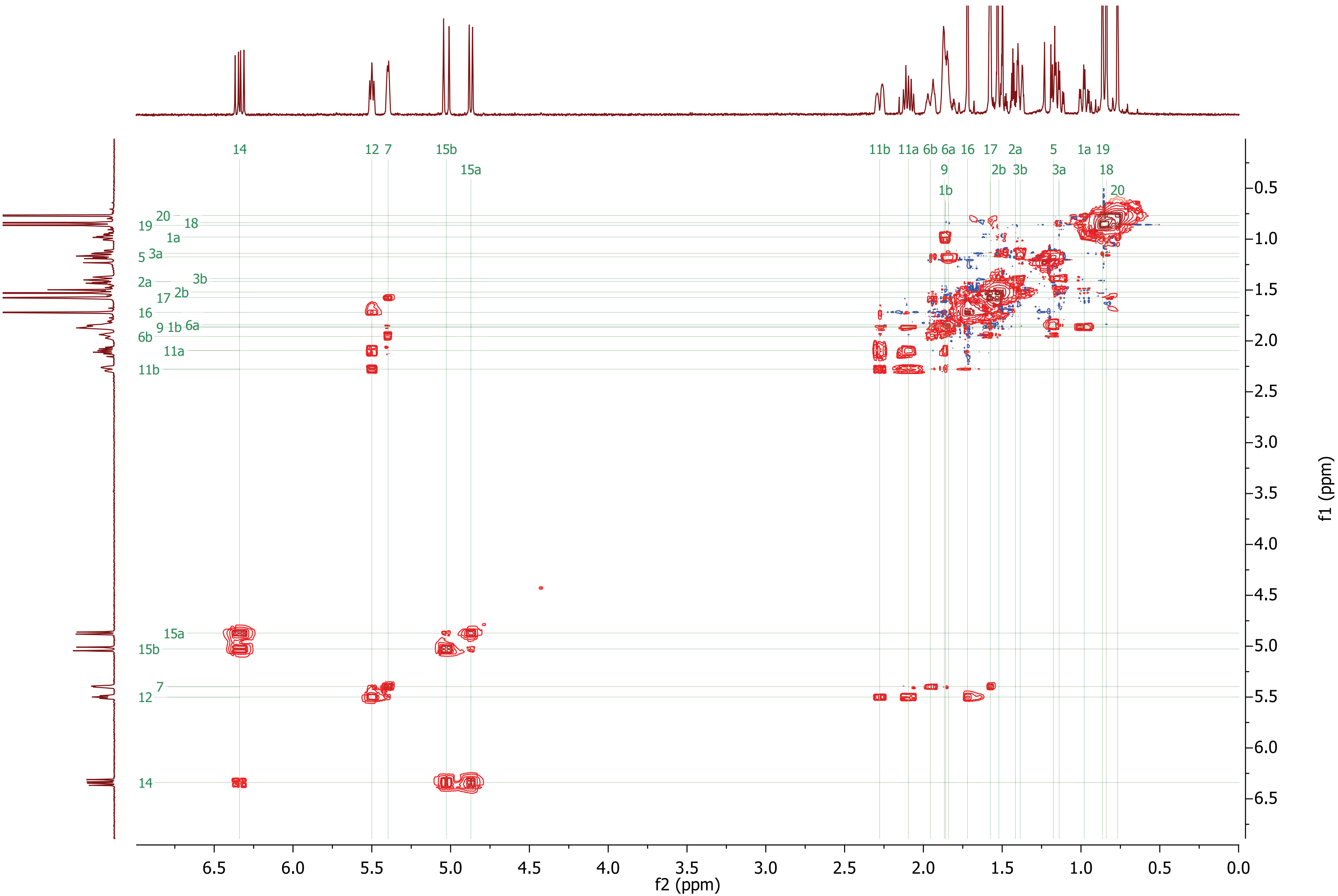

Figure S13-C. ^{1}H-^{1}H COSY of labda-7,12E,14-triene [**24**].

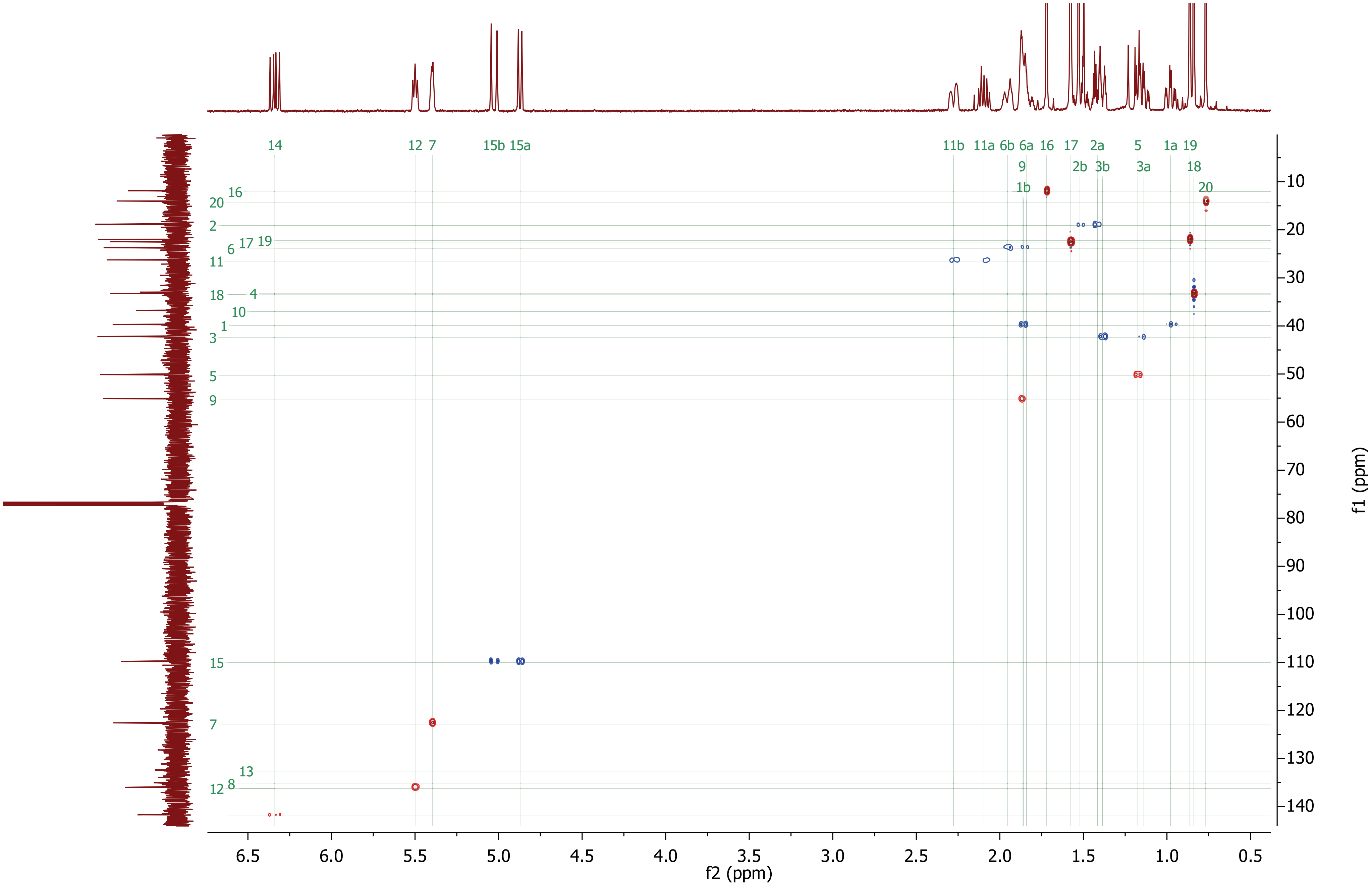

Figure S13-D. ^{1}H-^{13}C HSQC of labda-7,12E,14-triene [**24**].

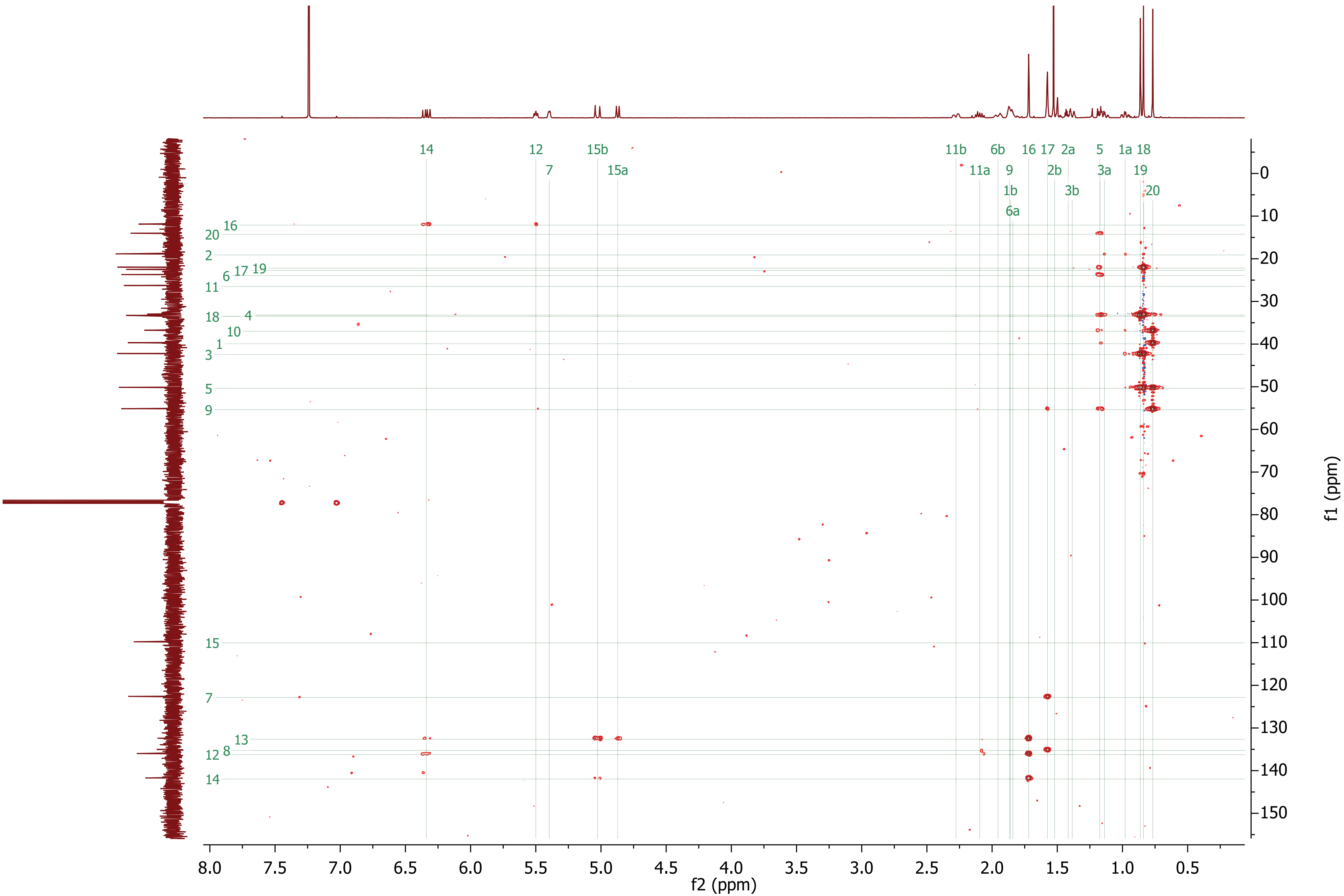

Figure S13-E. ^{1}H-^{13}C HMBC of labda-7,12E,14-triene [**24**].

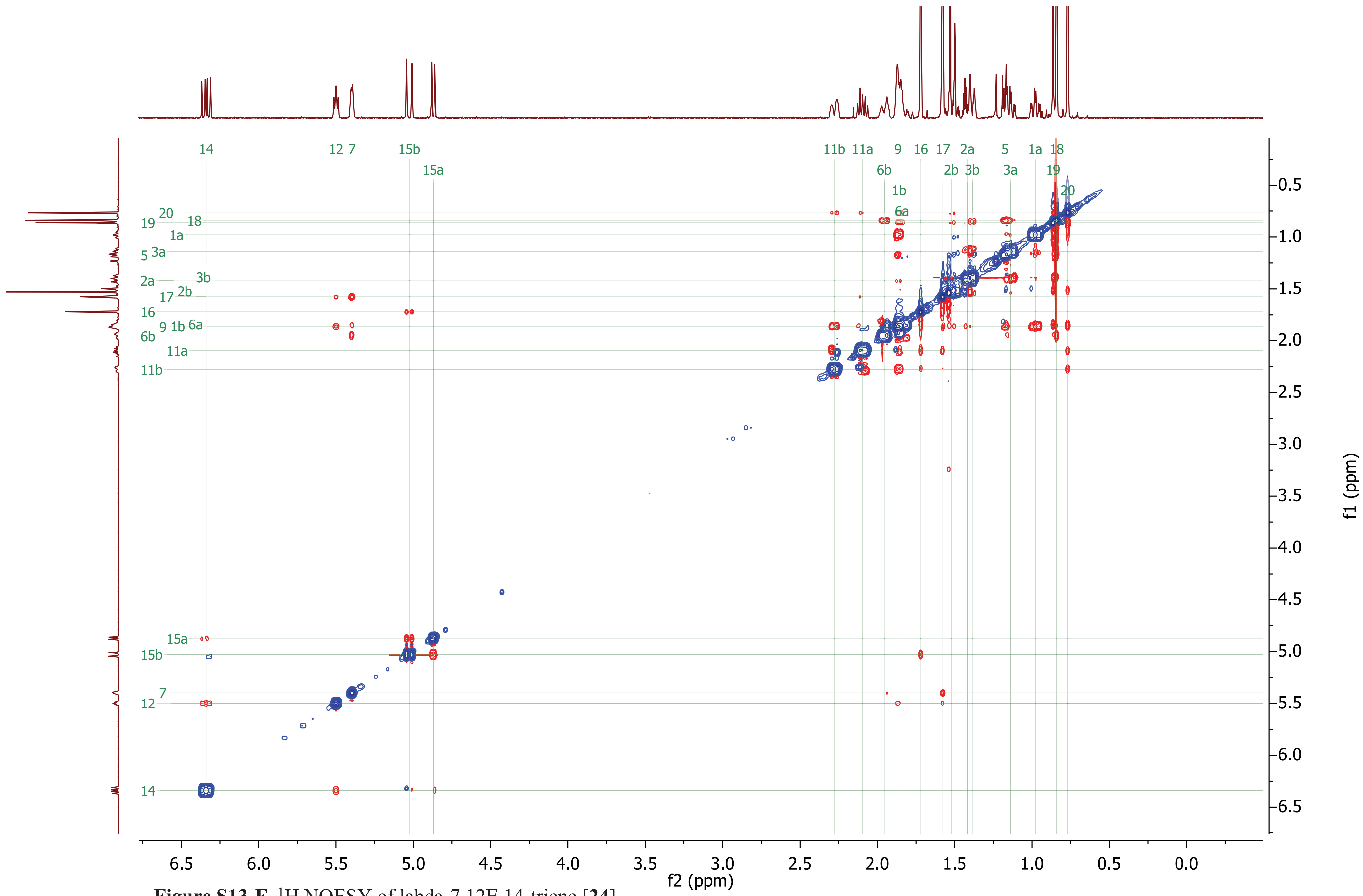

Figure S13-F. ^{1}H NOESY of labda-7,12E,14-triene [**24**].

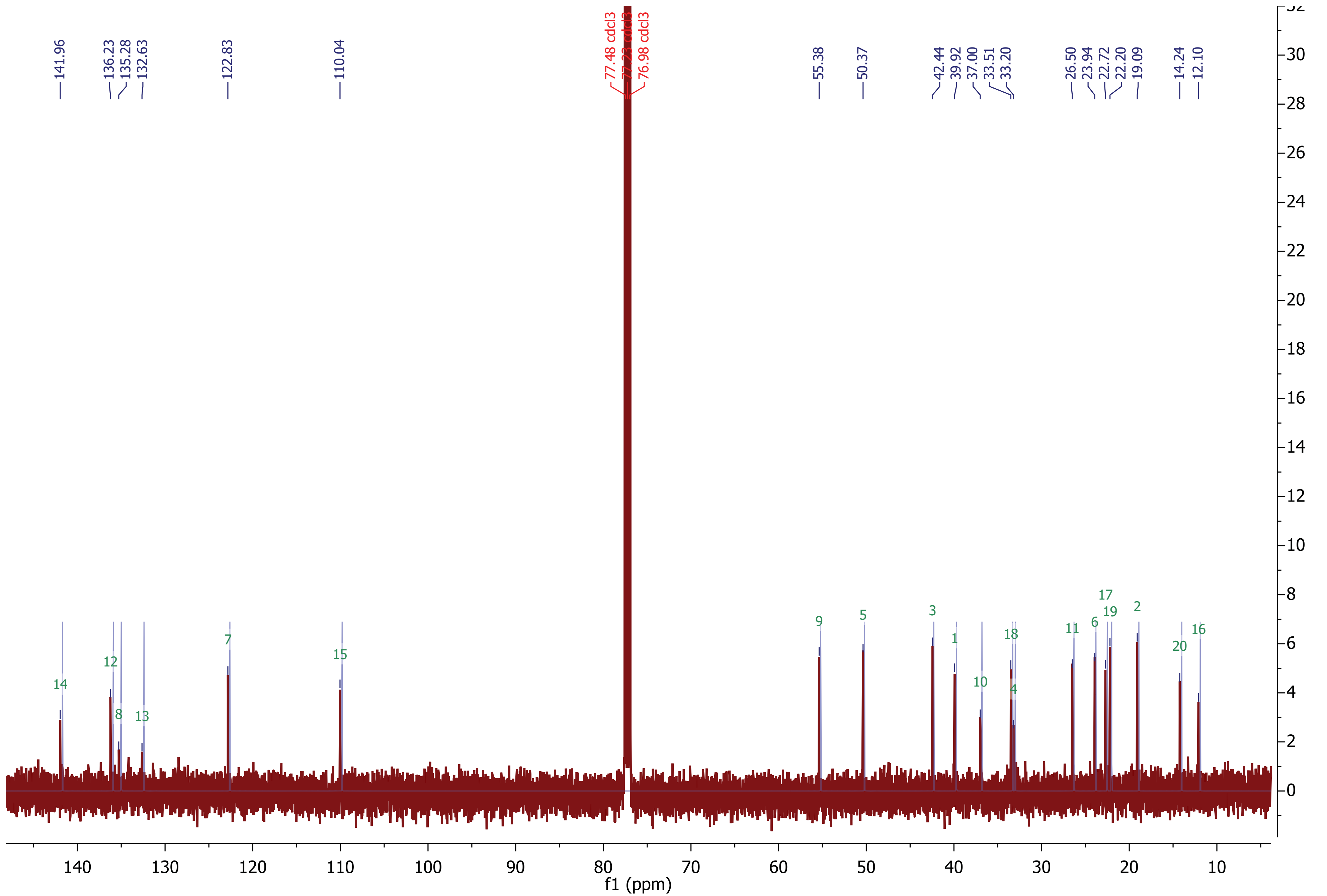

Figure S13-G. Overlay of ^{13}C NMR of labda-7,12E,14-triene [**24**] (red) with ^{13}C NMR spectrum (blue) reconstructed from shifts reported for the same compound by Roengsumran et al. (1999) (DOI: 10.1016/S0031-9422(98)00604-9).

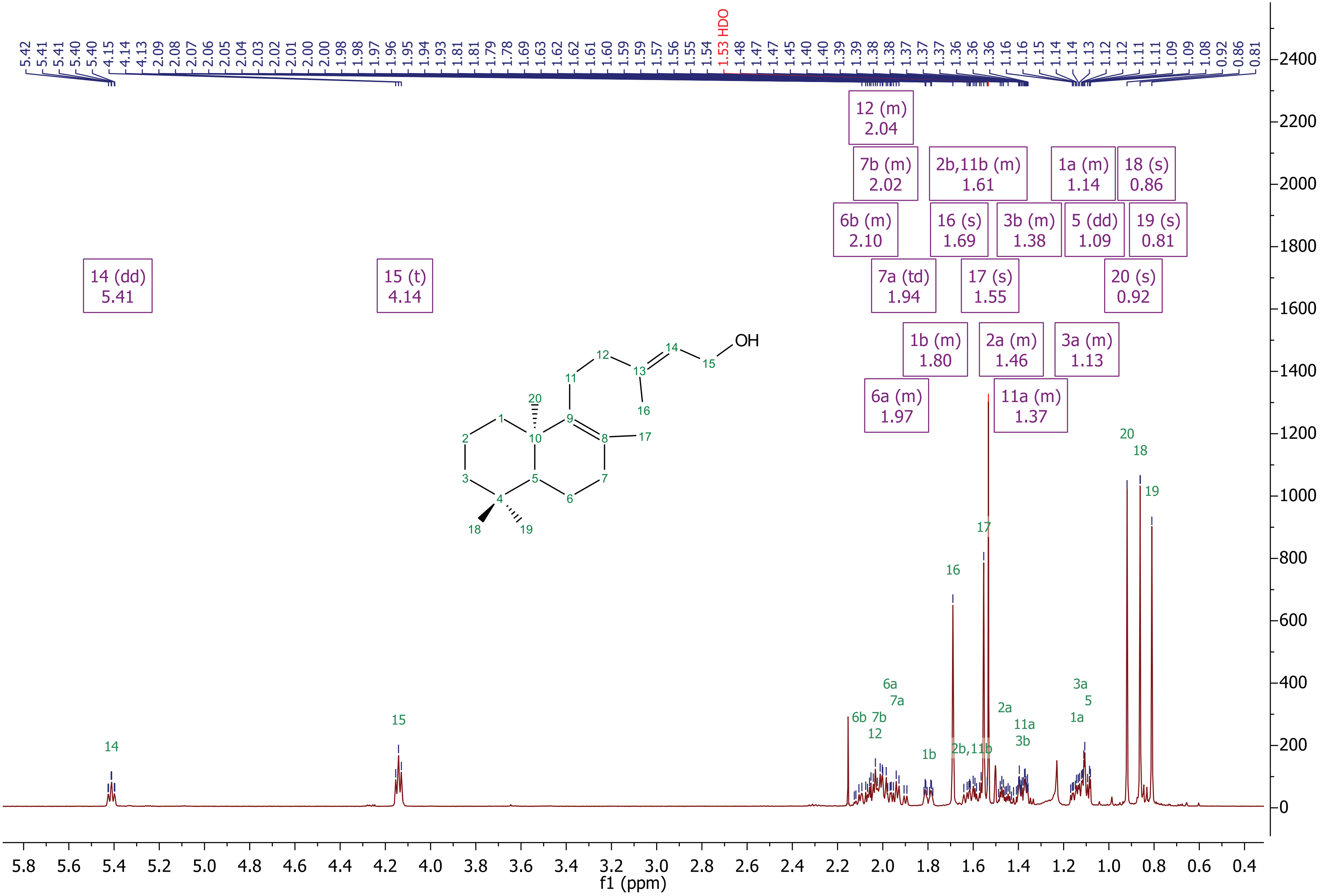

Figure S14-A. ^{1}H NMR of (10R)-labda-8,13E-diene-15-ol [**25a**].

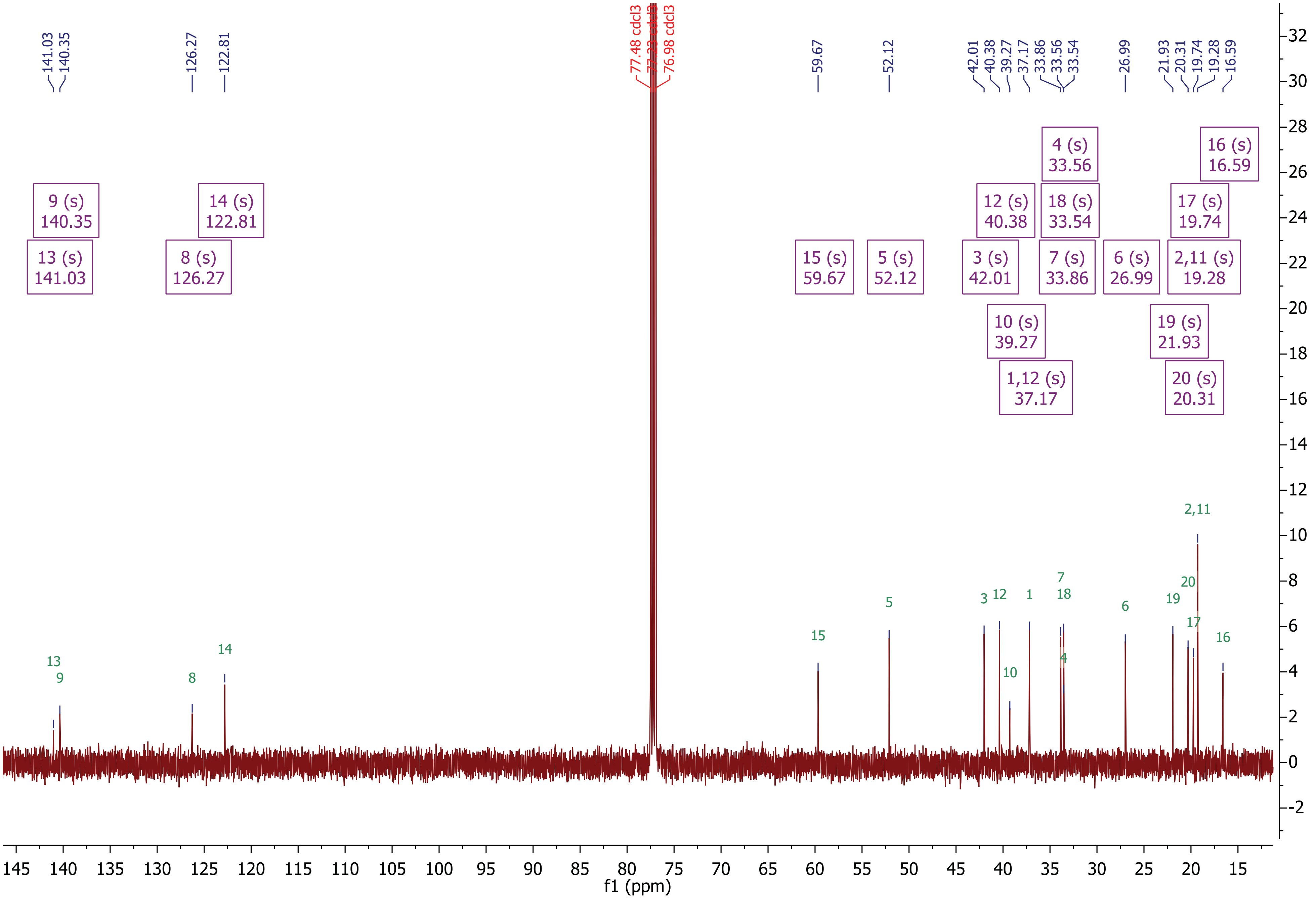

Figure S14-B. ^{13}C NMR of (10R)-labda-8,13E-diene-15-ol [**25a**].

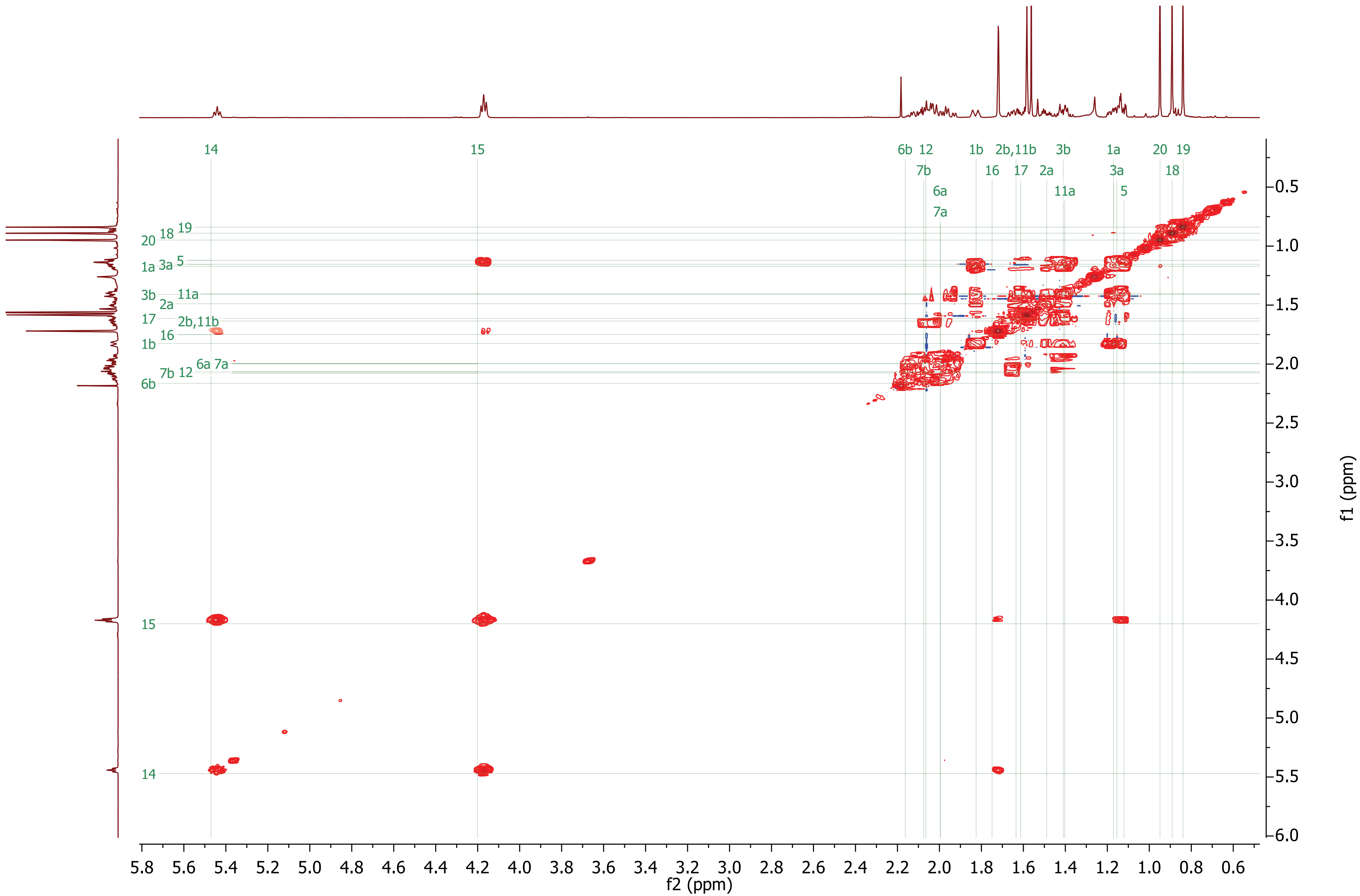

Figure S14-C. ¹H-¹H COSY of (10R)-labda-8,13E-diene-15-ol [**25a**].

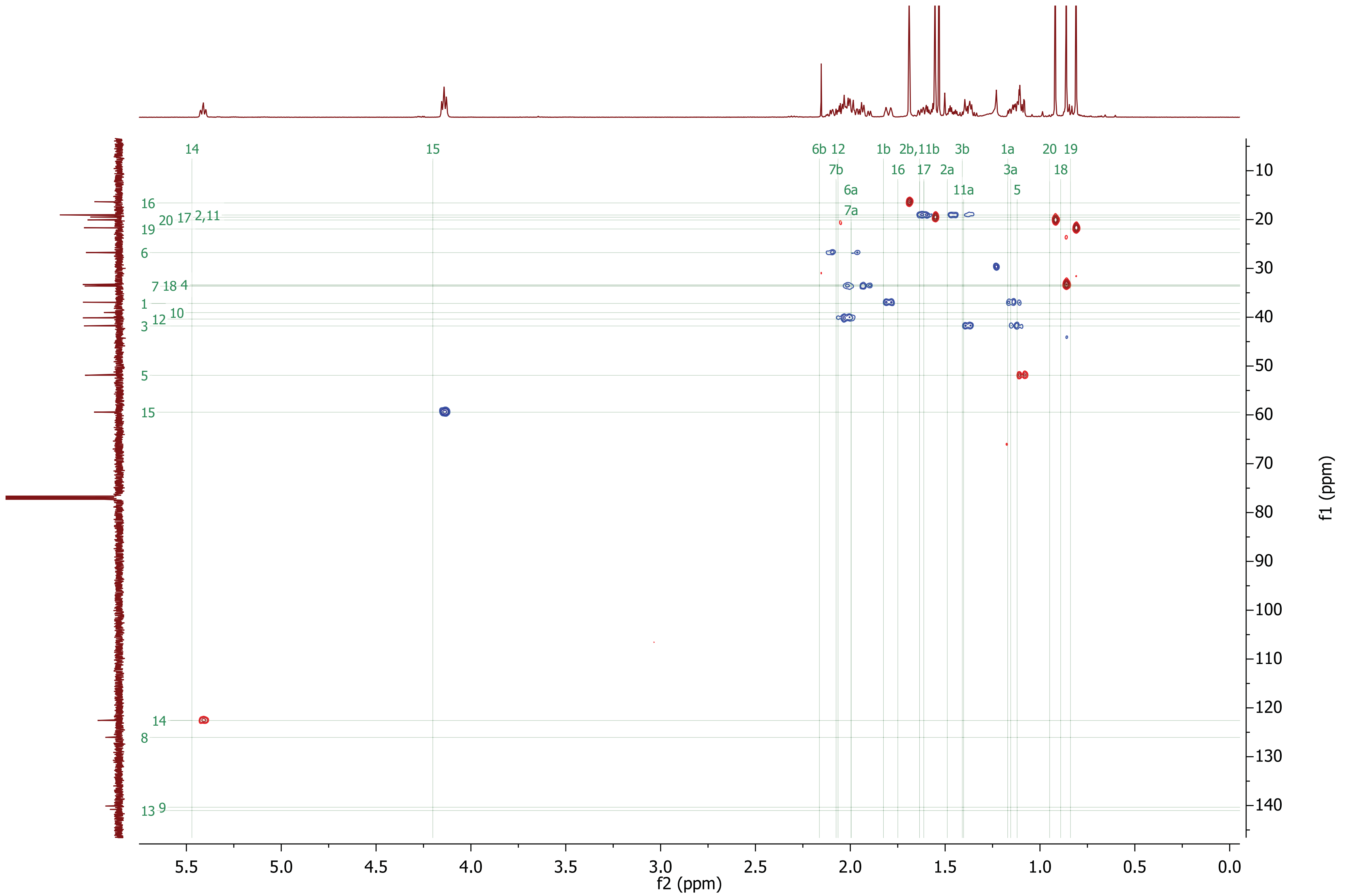

Figure S14-D. ^{1}H-^{13}C HSQC of (10R)-labda-8,13E-diene-15-ol [**25a**].

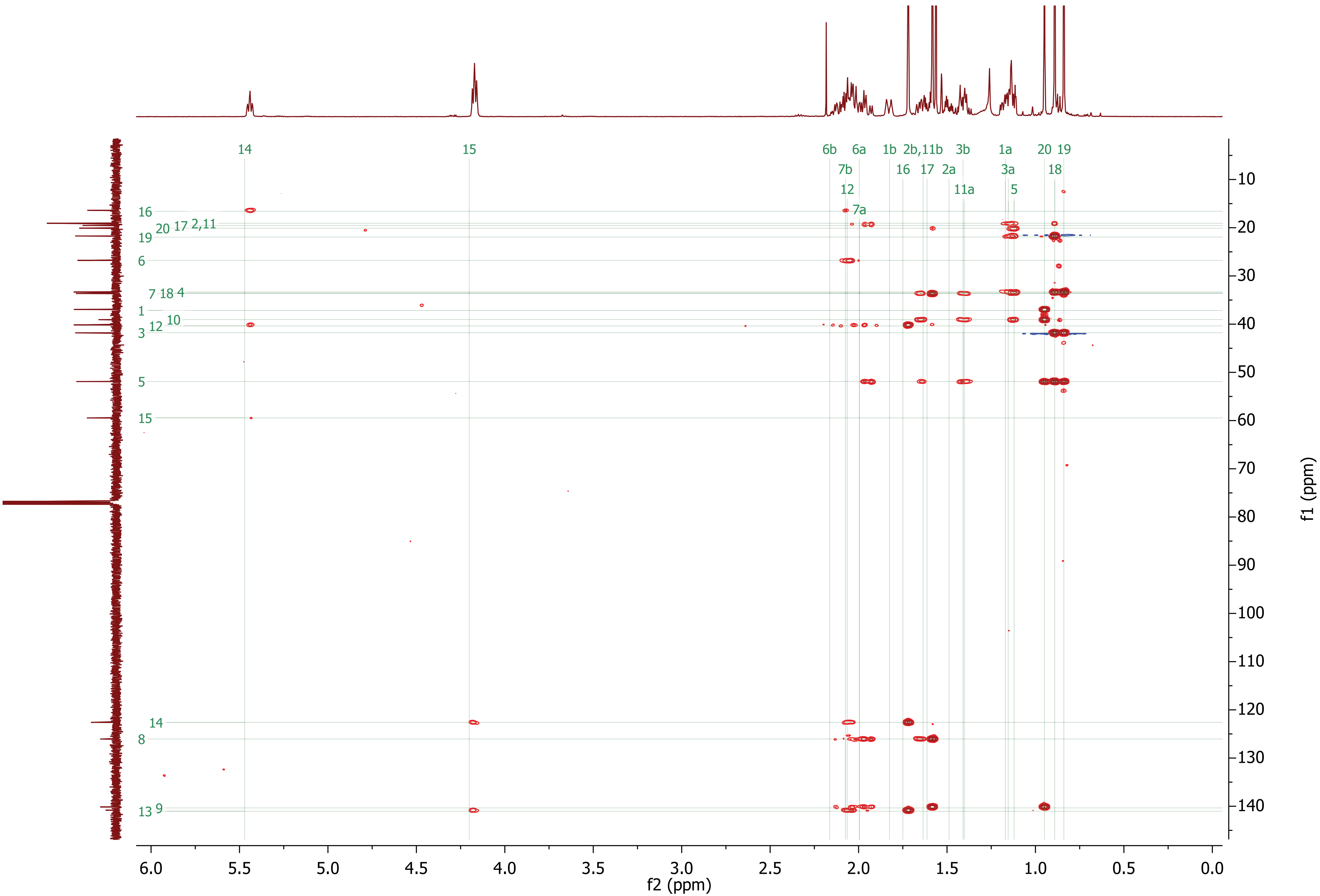

Figure S14-E. ^{1}H-^{13}C HMBC of (10R)-labda-8,13E-diene-15-ol [**25a**].

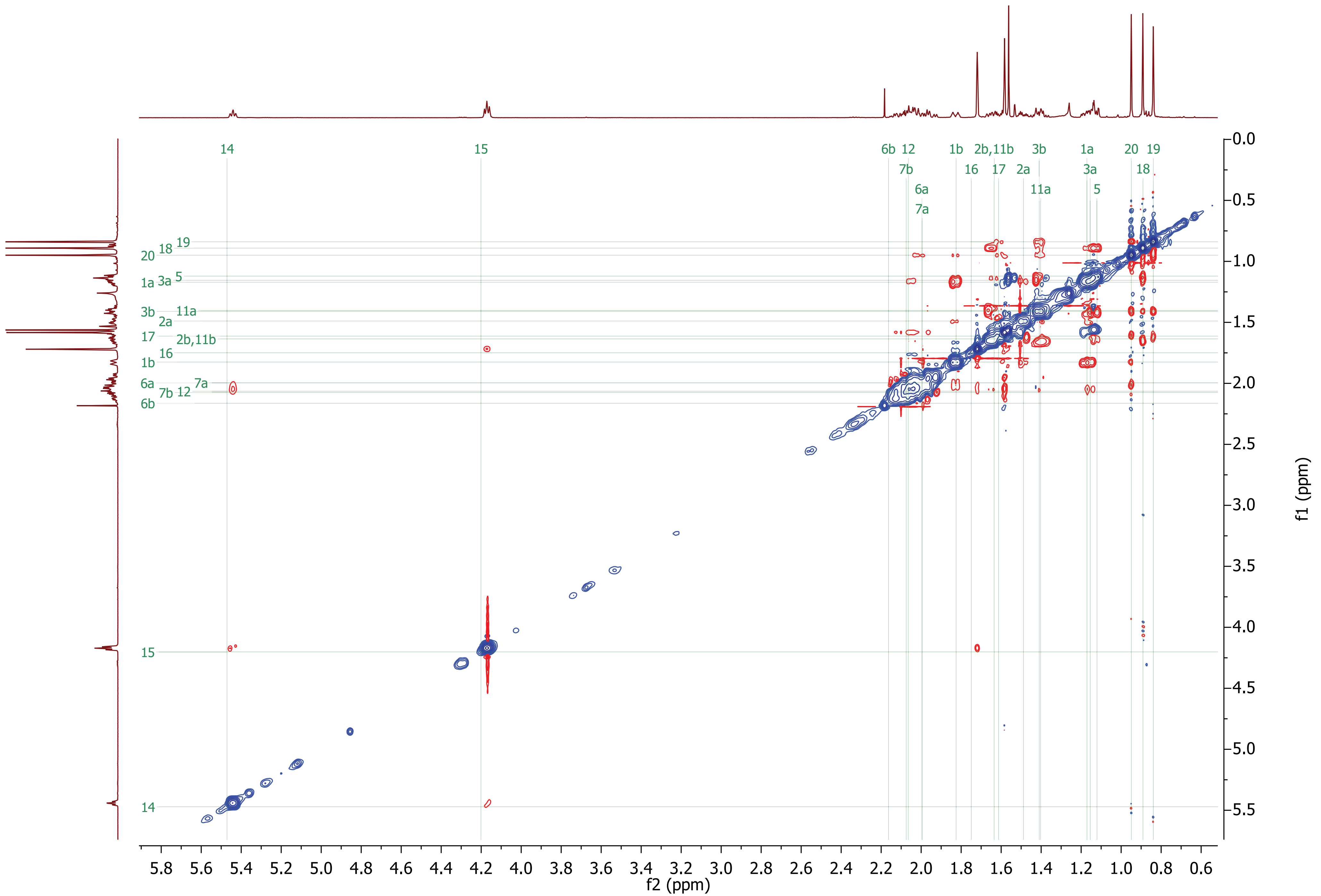

Figure S14-F. ^{1}H NOESY of (10R)-labda-8,13E-diene-15-ol [**25a**].

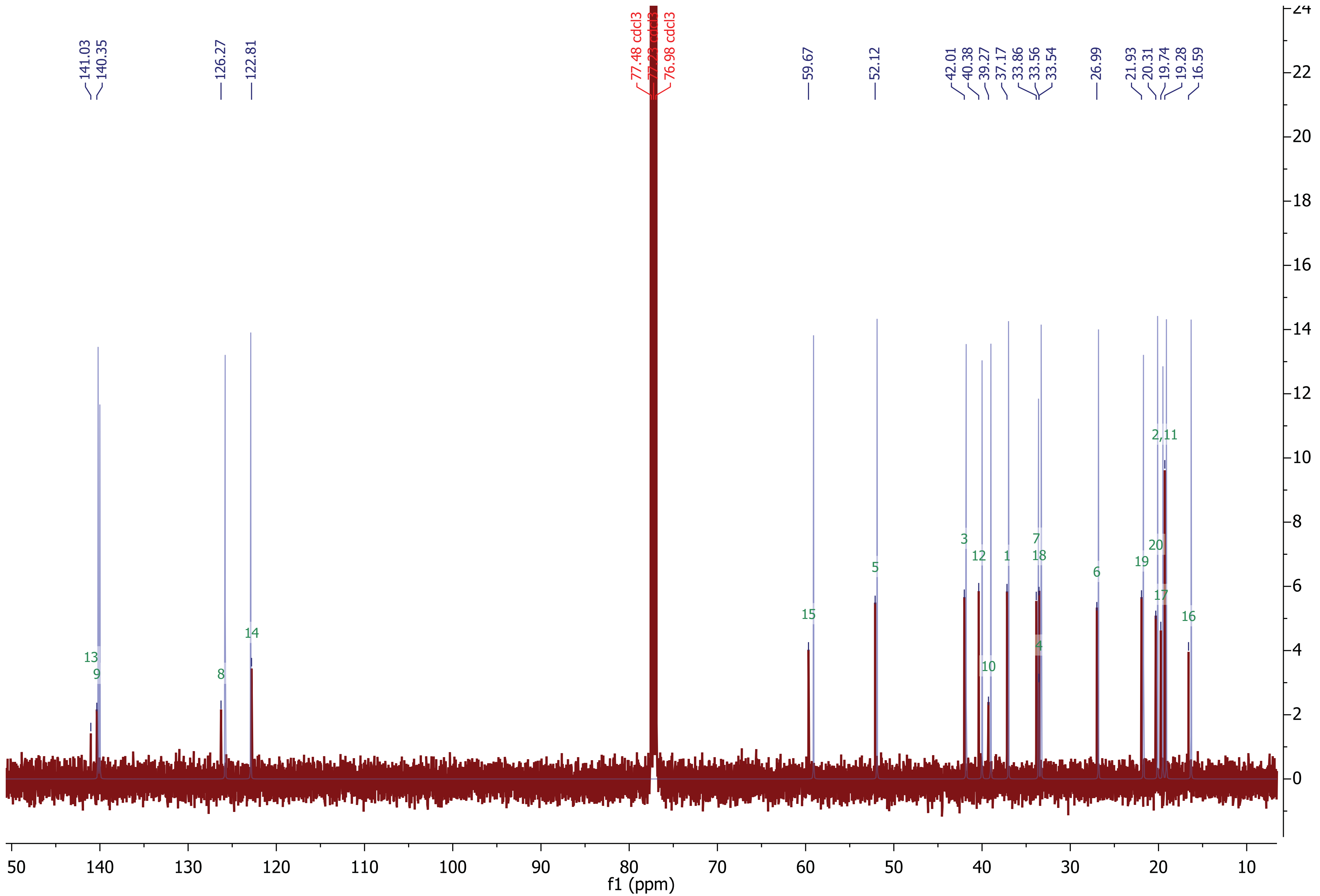

Figure S14-G. Overlay of ^{13}C NMR of (10R)-labda-8,13E-diene-15-ol [**25a**] (red) with ^{13}C NMR spectrum (blue) reconstructed from shifts reported for the same compound by Suzuki et al. (1983) (DOI: 10.1016/0031-9422(83)80249-0).

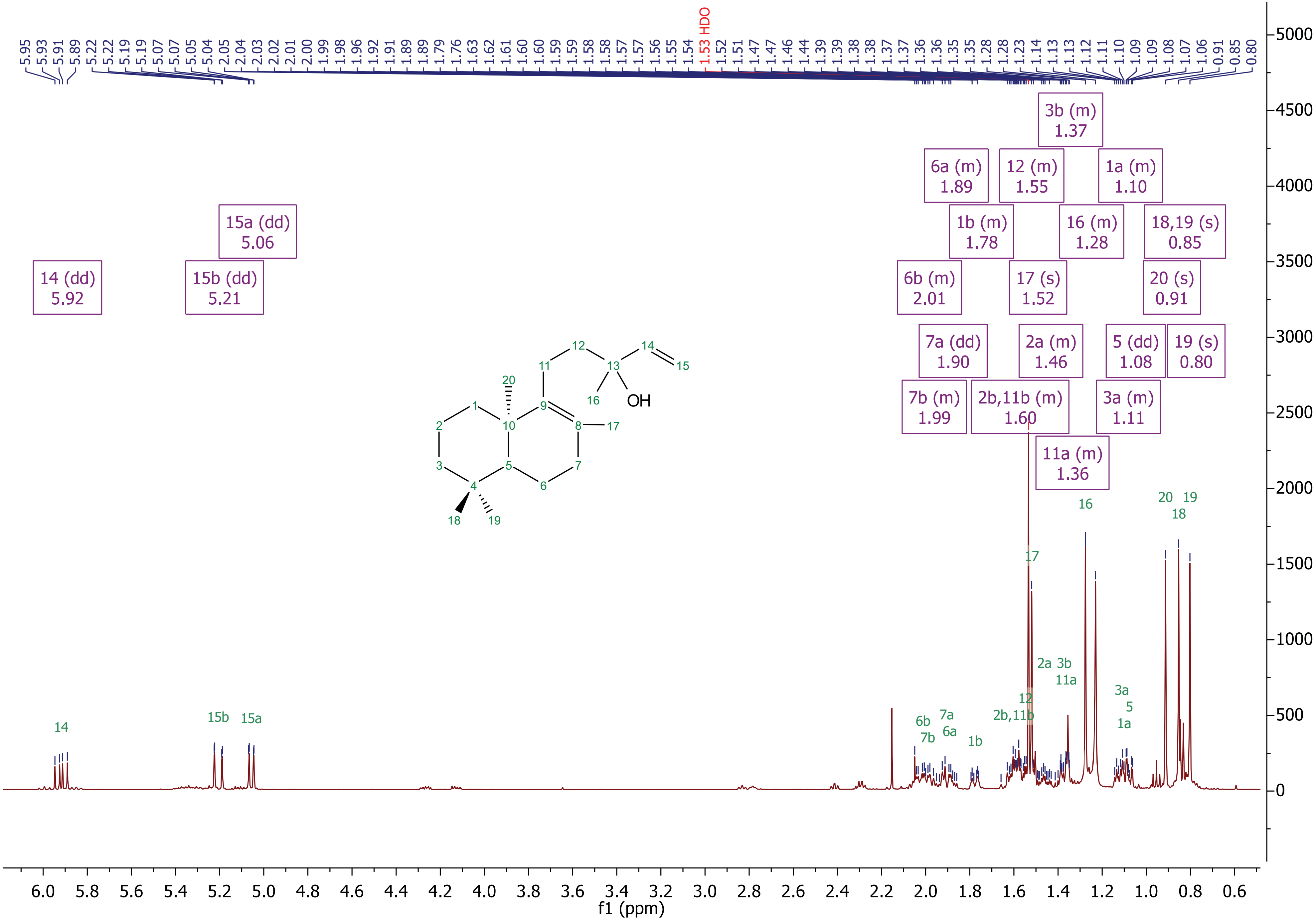

Figure S15-A. ^{1}H NMR of (10R)-labda-8,14-dien-13-ol [**26**].

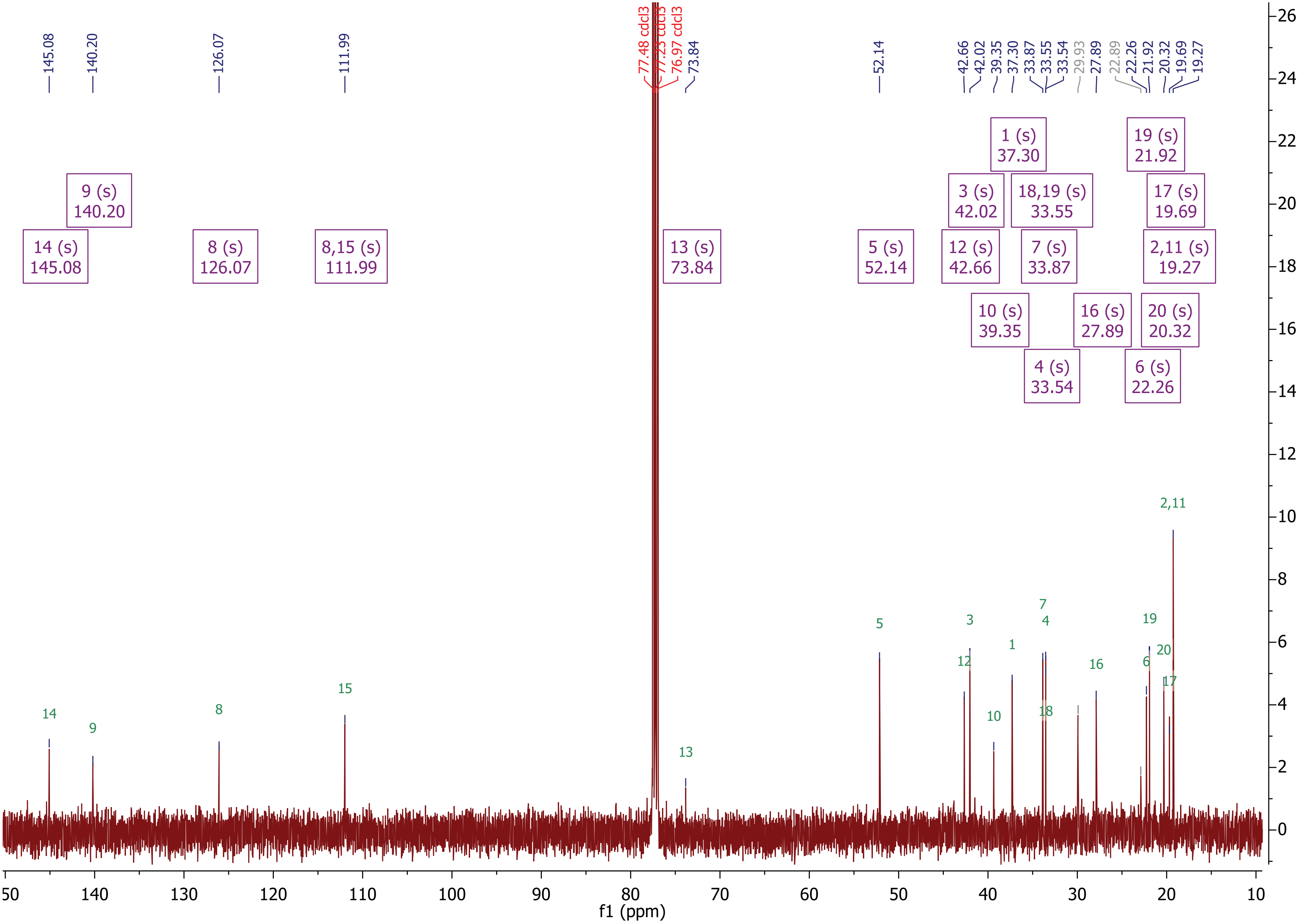

Figure S15-B. ^{13}C NMR of (10R)-labda-8,14-dien-13-ol [**26**].

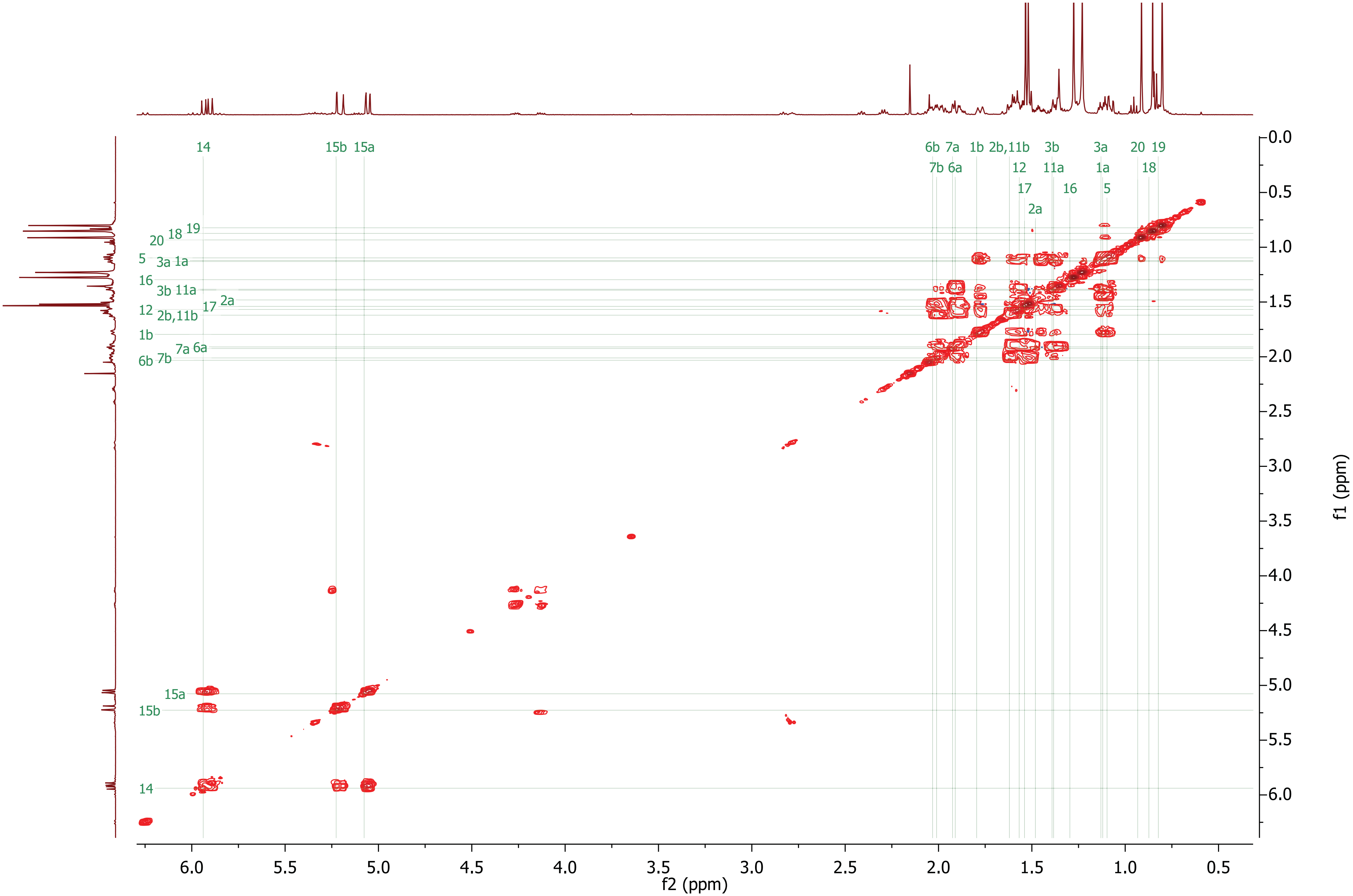

Figure S15-C. ^{1}H-^{1}H COSY of (10R)-labda-8,14-dien-13-ol [**26**].

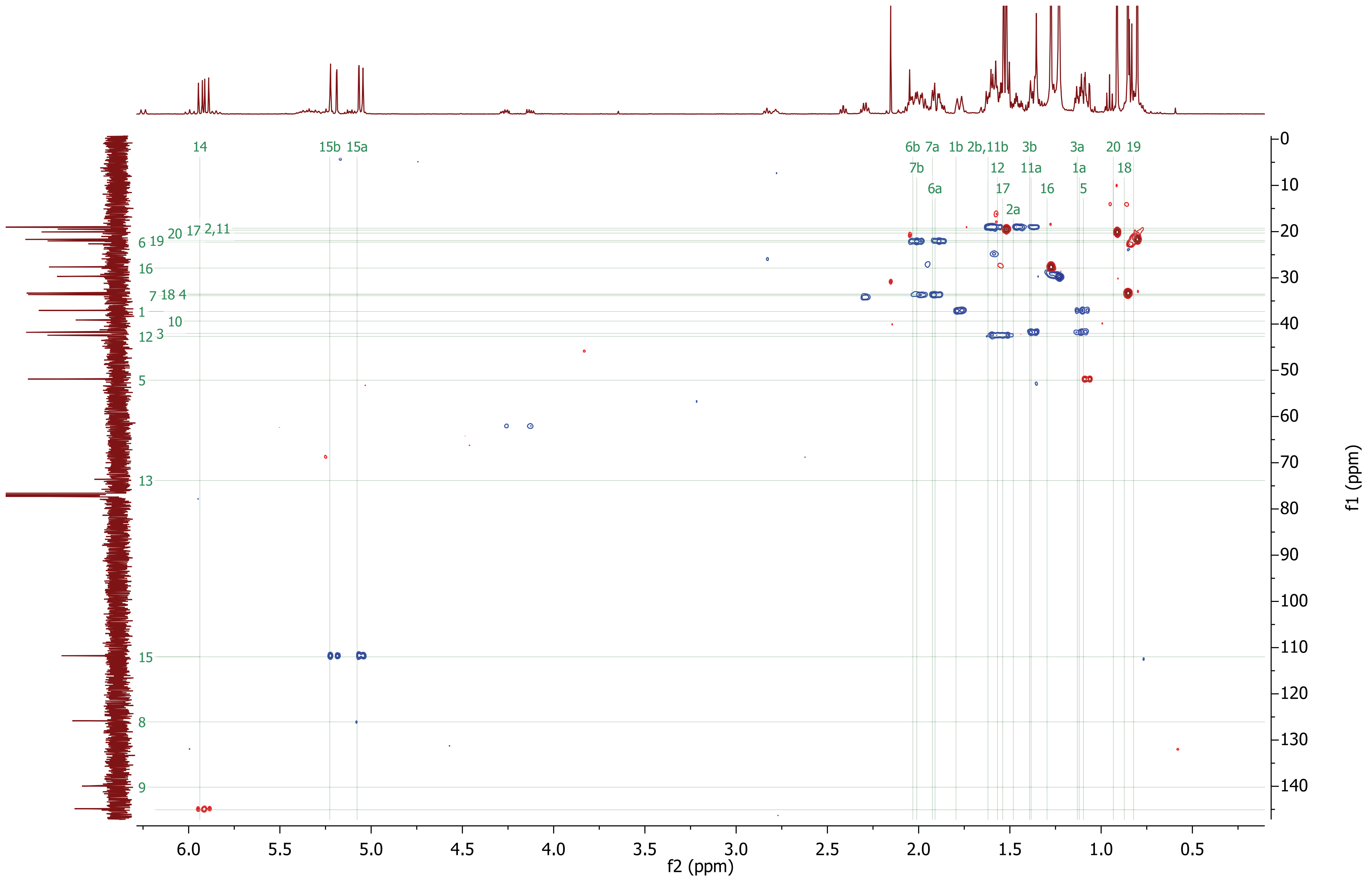

Figure S15-D. ^{1}H-^{13}C HSQC of (10R)-labda-8,14-dien-13-ol [**26**].

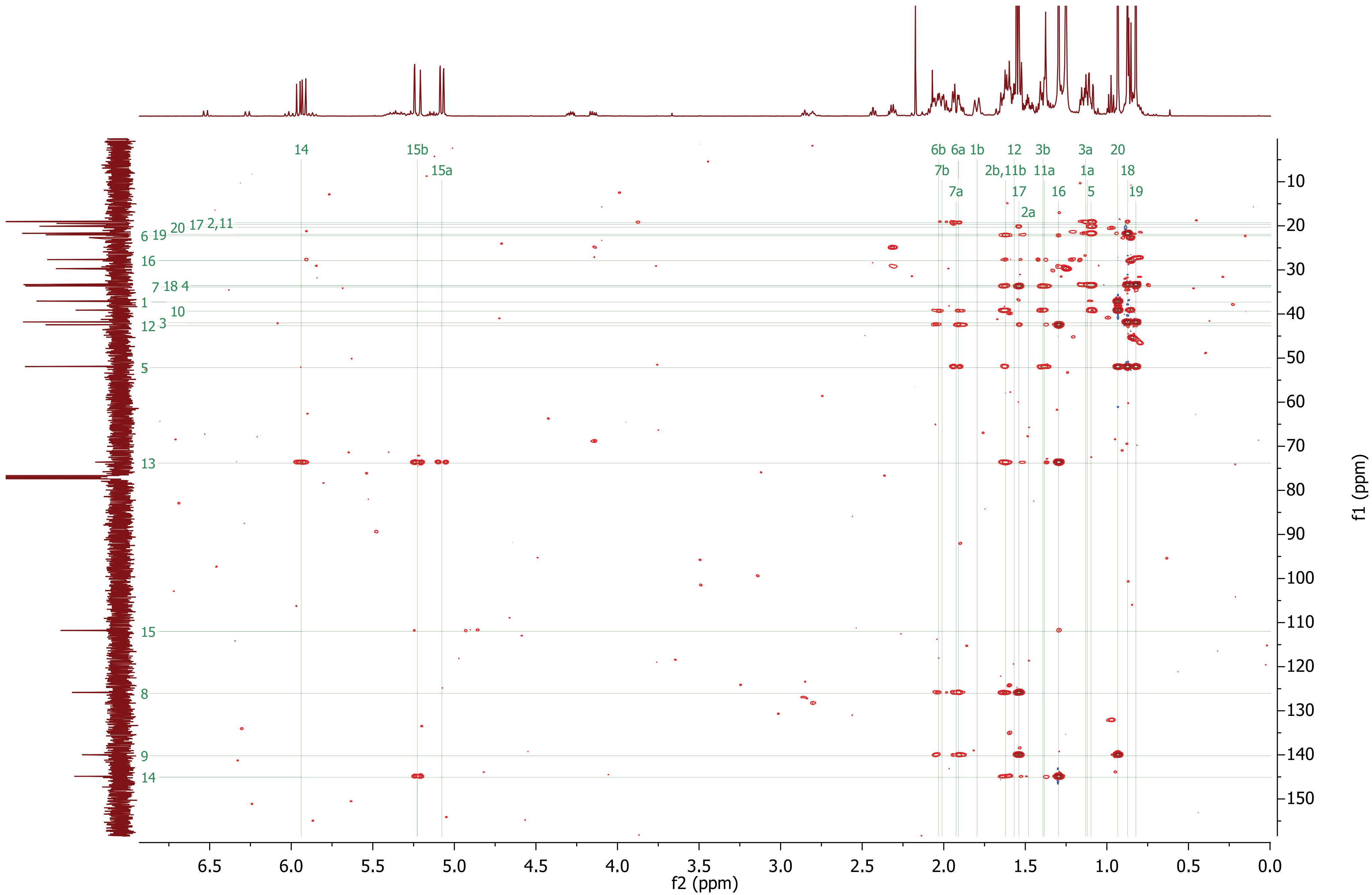

Figure S15-E. ^{1}H-^{13}C HMBC of (10R)-labda-8,14-dien-13-ol [**26**].

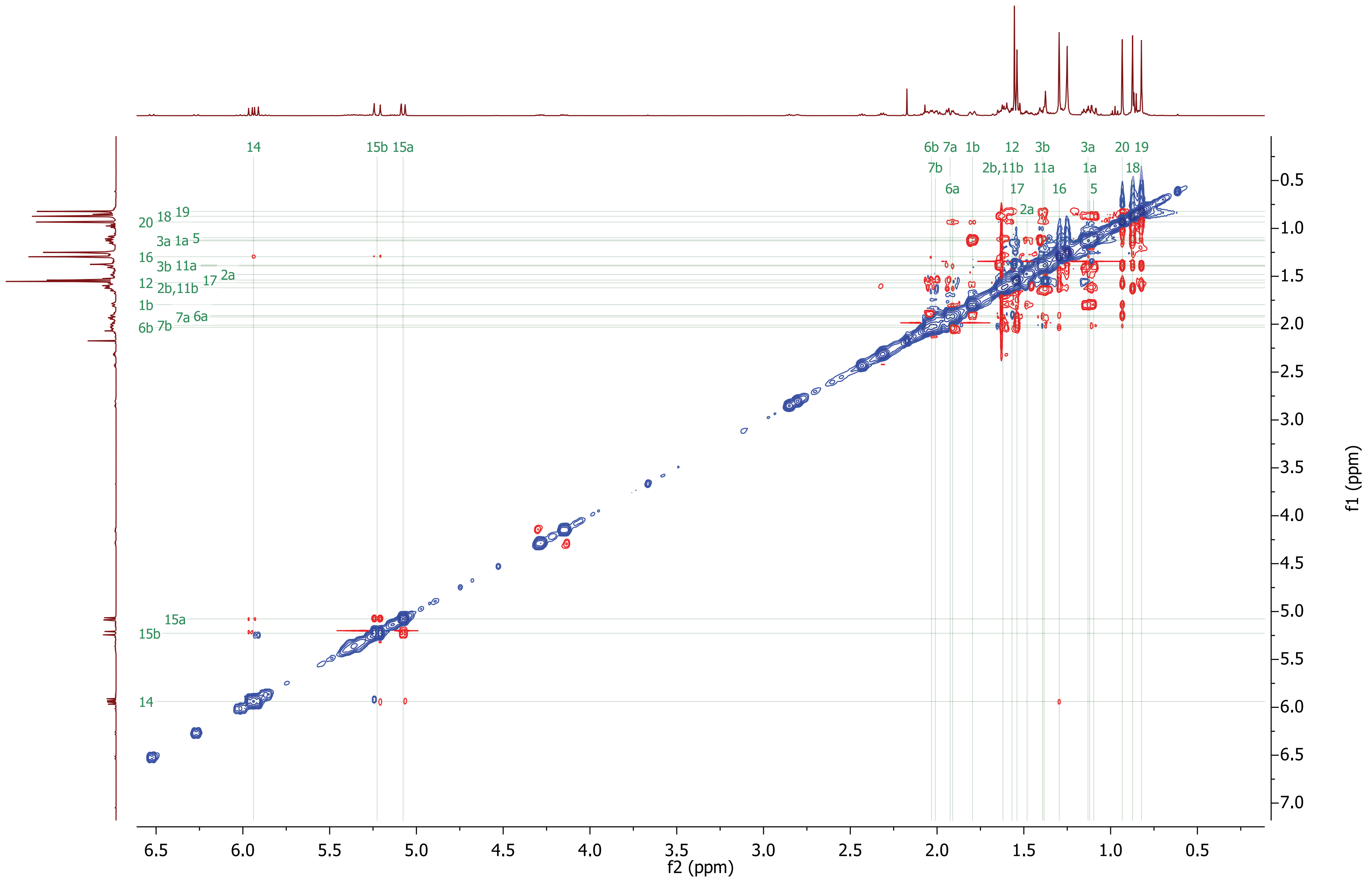

Figure S15-F. ^{1}H NOESY of (10R)-labda-8,14-dien-13-ol [**26**].

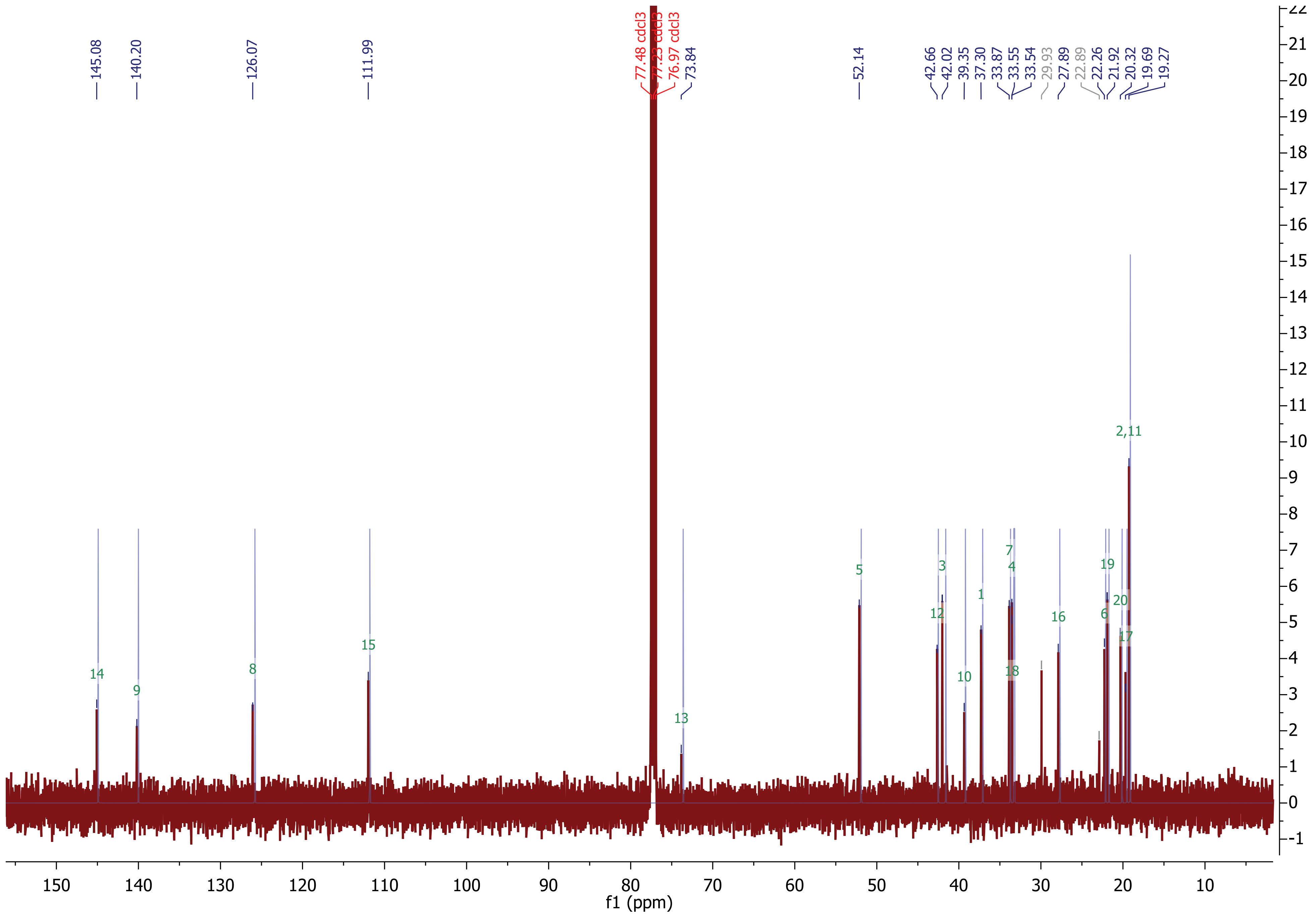

Figure S15-G. Overlay of ^{13}C NMR of (10R)-labda-8,14-dien-13-ol [**26**] (red) with ^{13}C NMR spectrum (blue) reconstructed from shifts reported for the same compound by Wu and Lin (1997) (DOI: 10.1016/S0031-9422(96)00519-5).

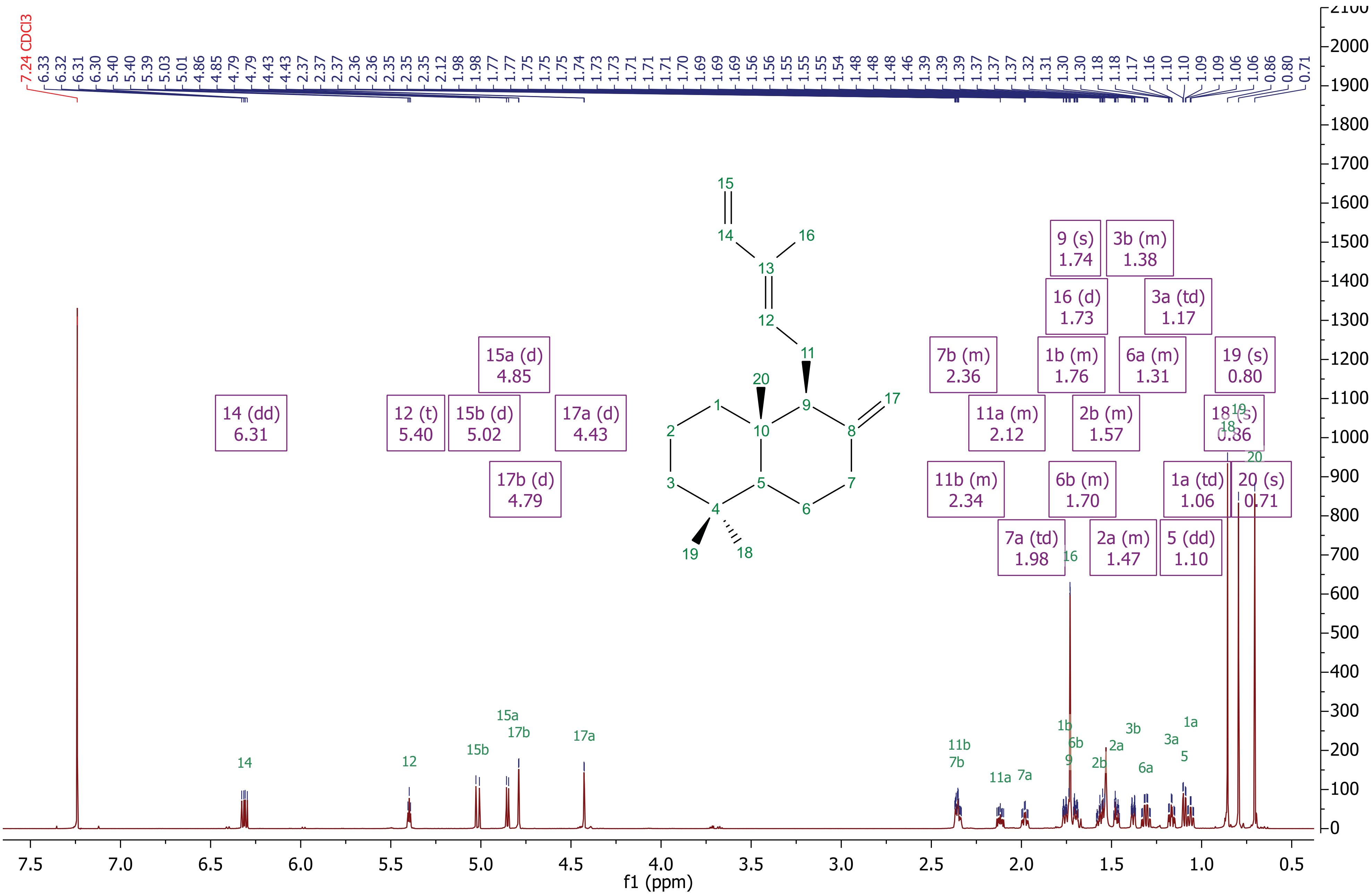

Figure S16-A. ^{1}H NMR of trans-biformene [**34**].

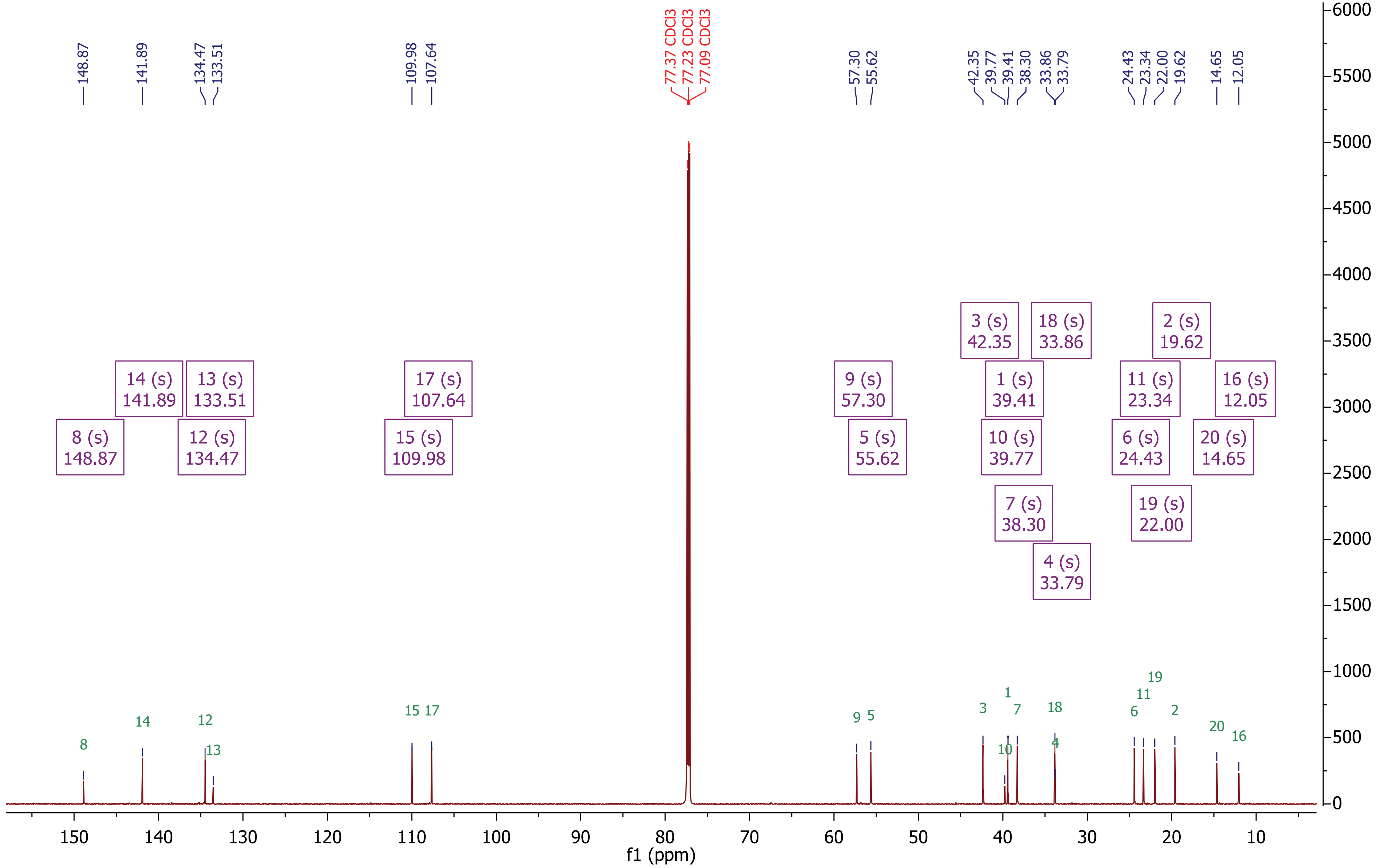

Figure S16-B. ^{13}C NMR of trans-biformene [**34**].

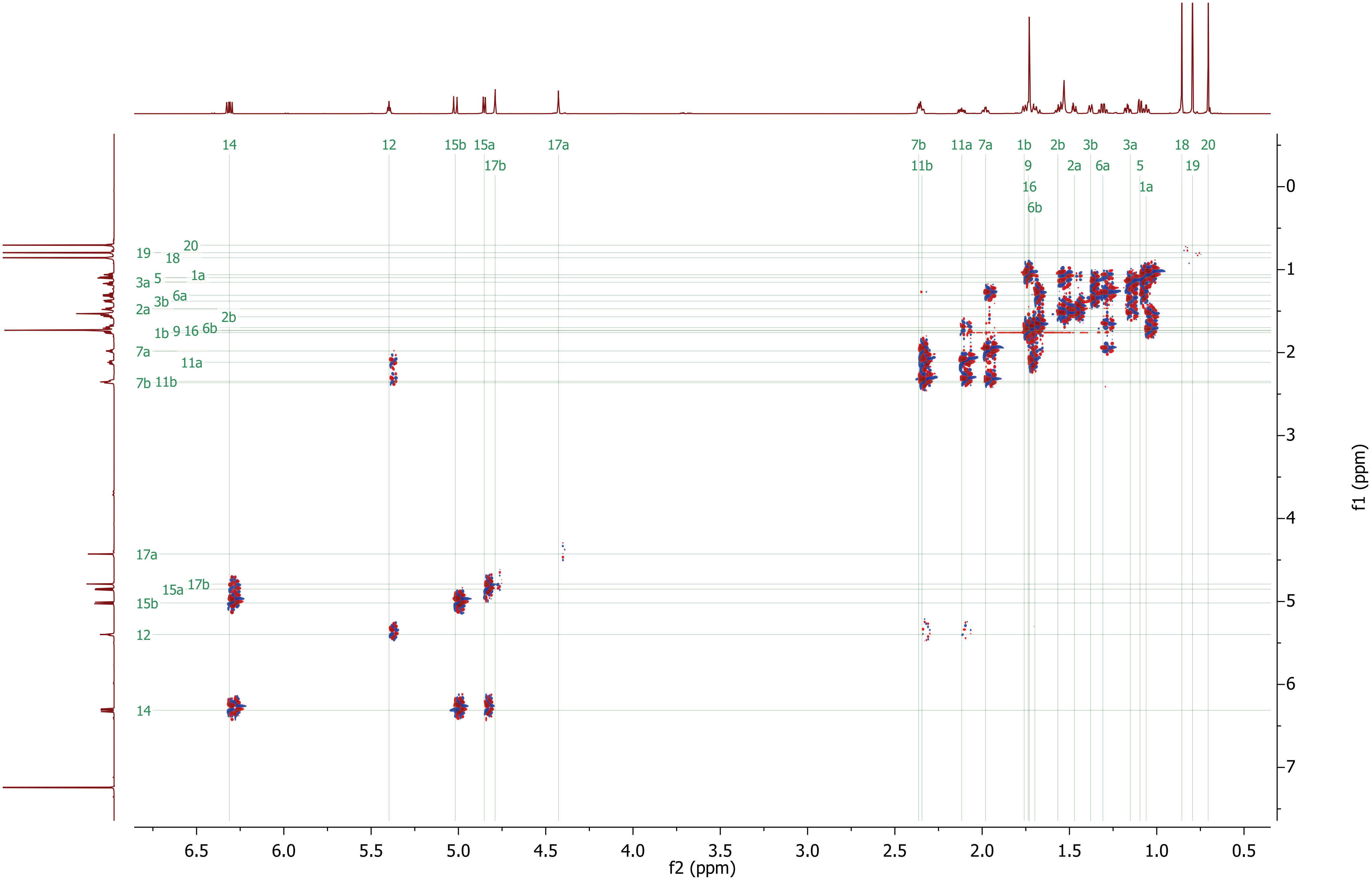

Figure S16-C. ^{1}H-^{1}H COSY of trans-biformene [**34**].

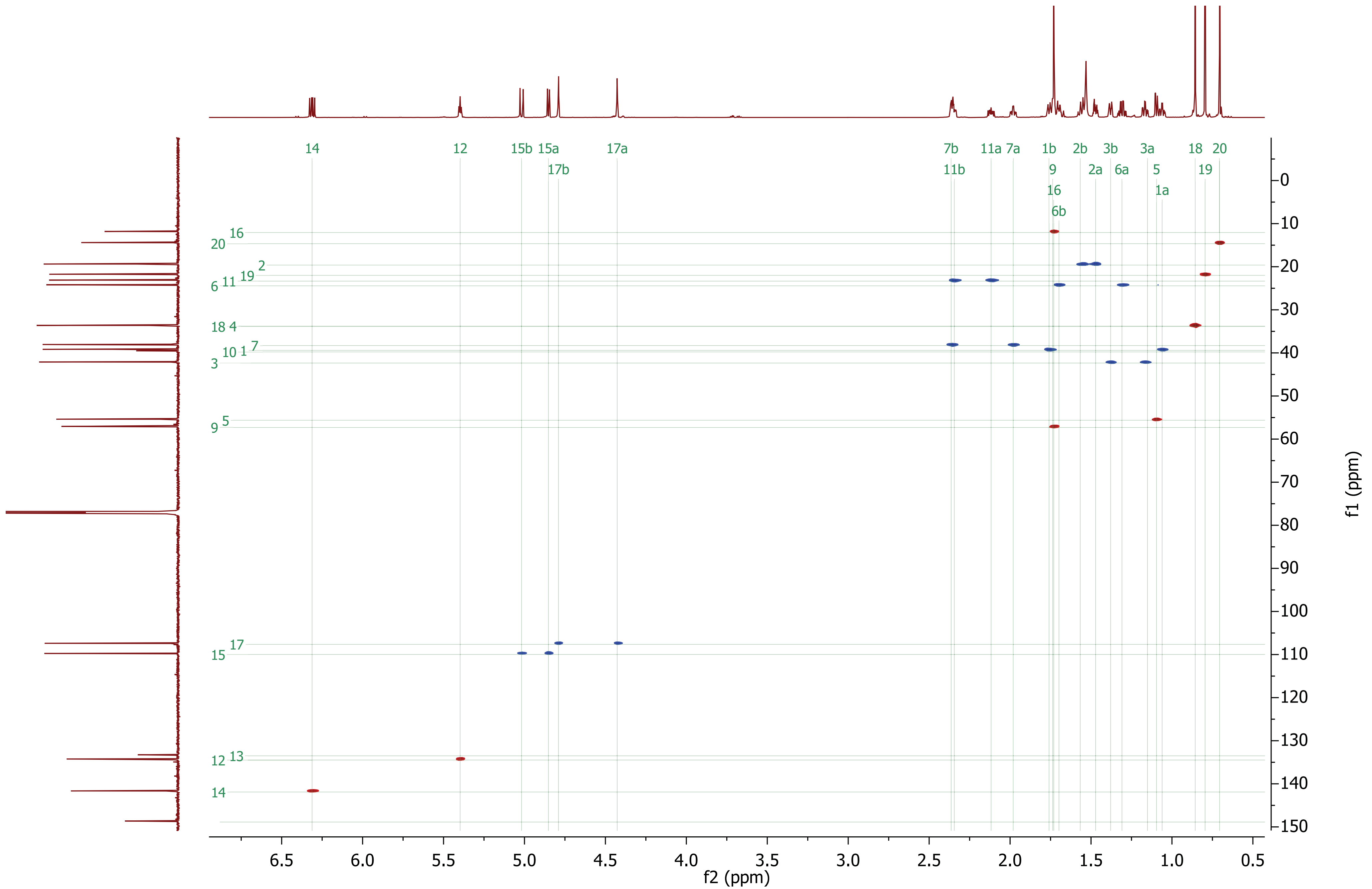

Figure S16-D. ^{1}H-^{13}C HSQC of trans-biformene [**34**].

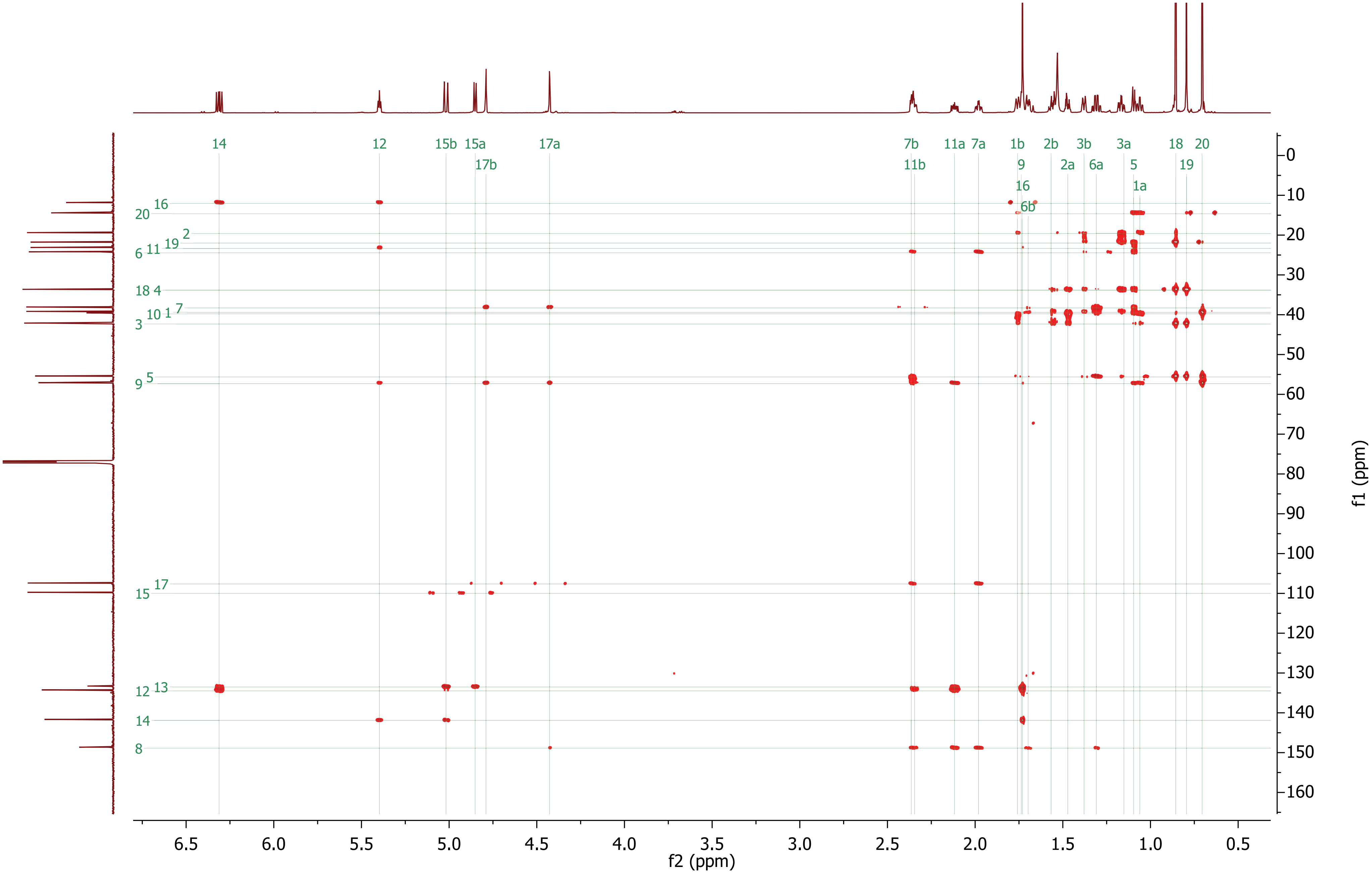

Figure S16-E. ^{1}H-^{13}C HMBC of trans-biformene [**34**].

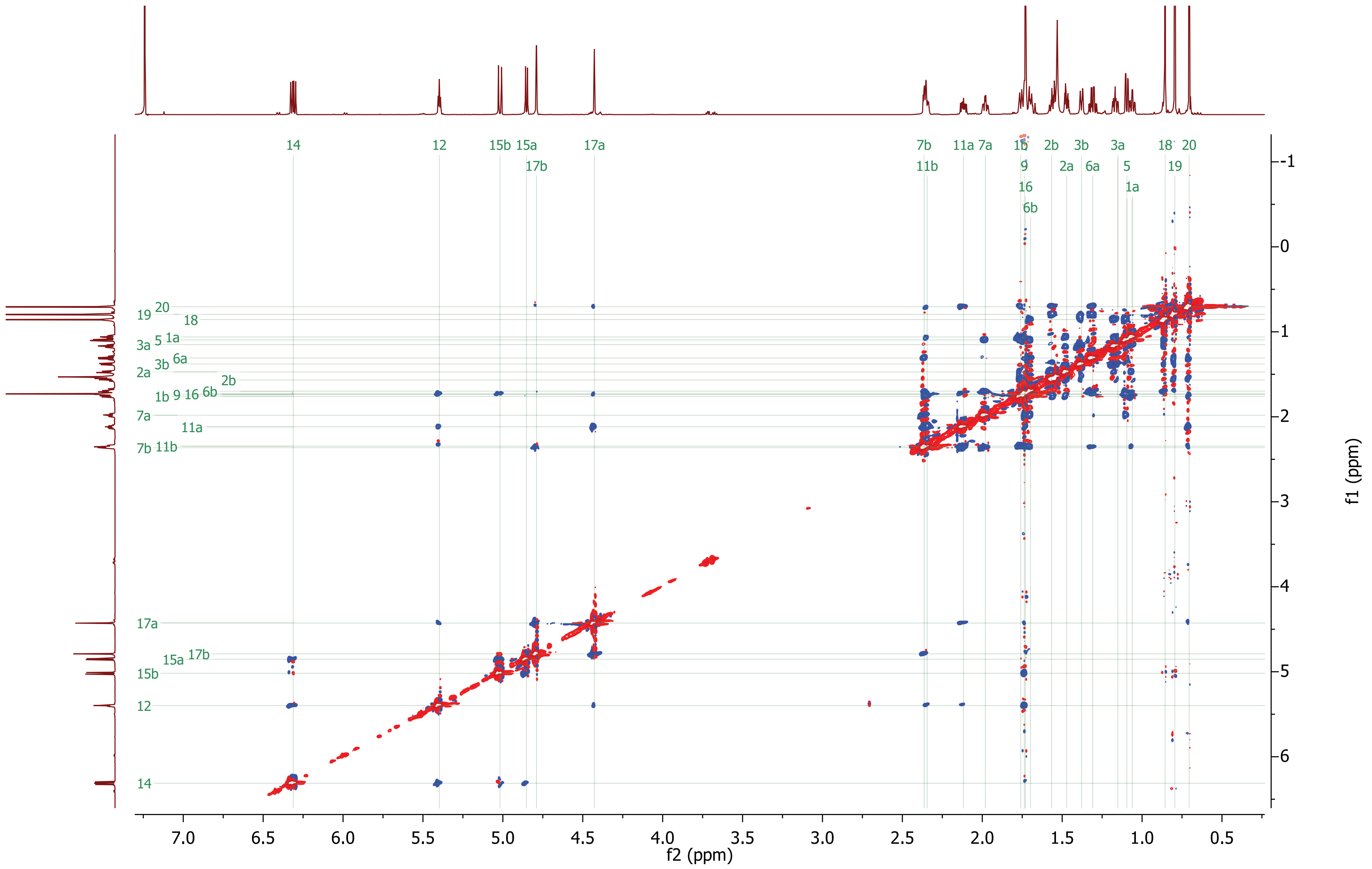

Figure S16-F. ^{1}H NOESY of trans-biformene [**34**].

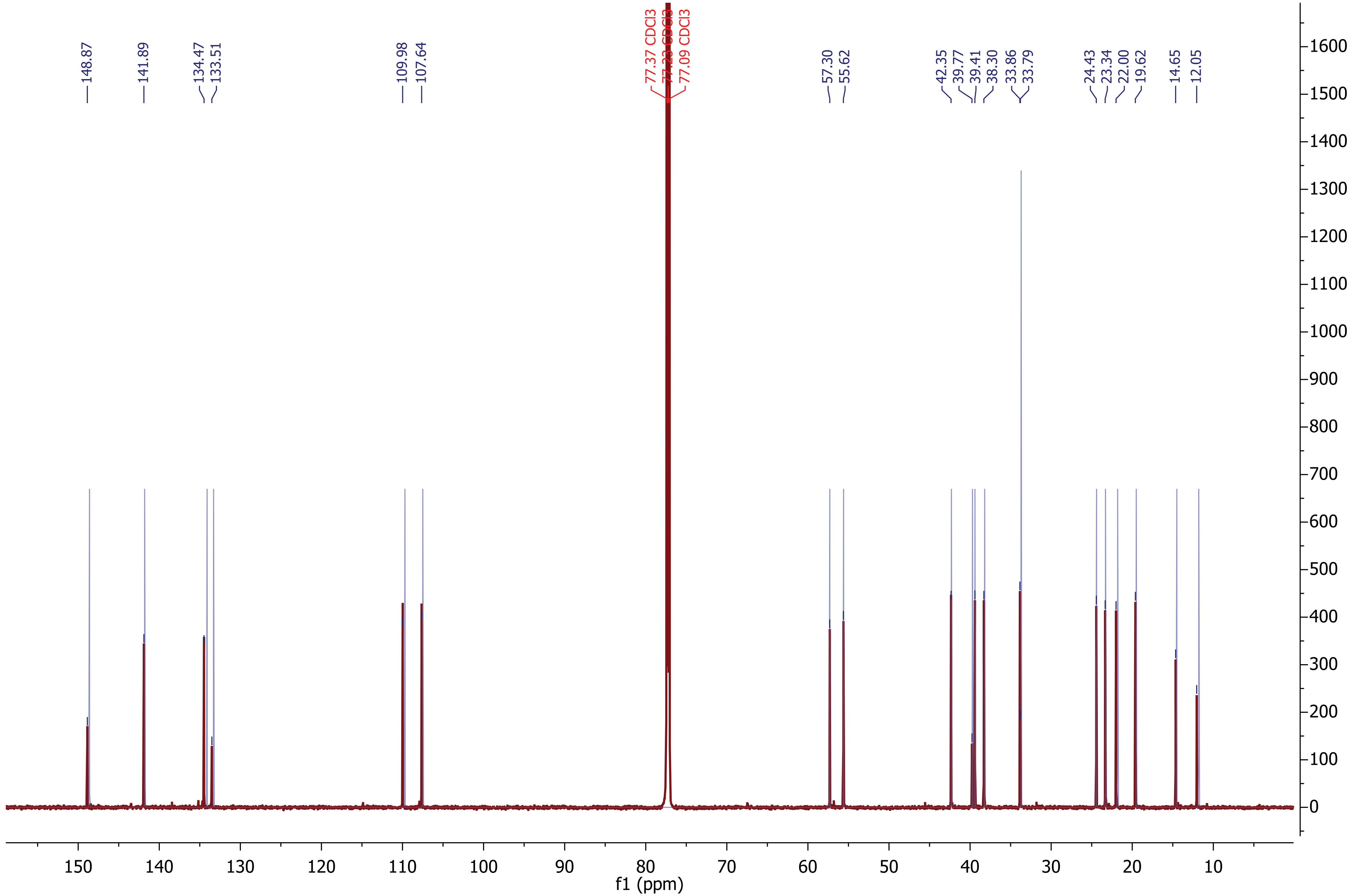

Figure S16-G. Overlay of ^{13}C NMR of trans-biformene [**34**] (red) with ^{13}C NMR spectrum (blue) reconstructed from shifts reported for the same compound by Bohlmann and Czerson (1979) (DOI: 10.1016/S0031-9422(00)90926-9).

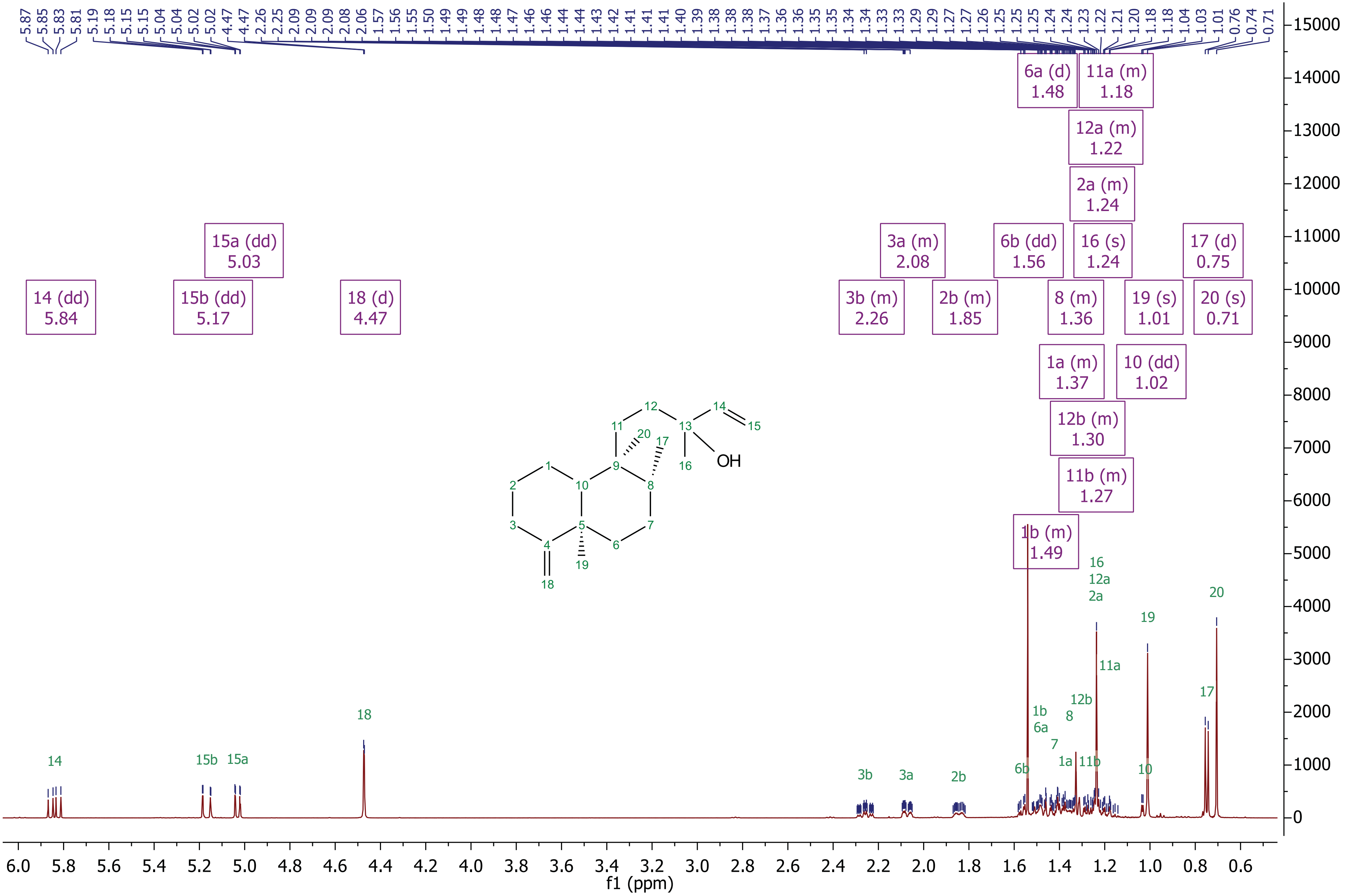

Figure S17-A. ^{1}H NMR of neo-cleroda-4(18),14-dien-13-ol [**37**].

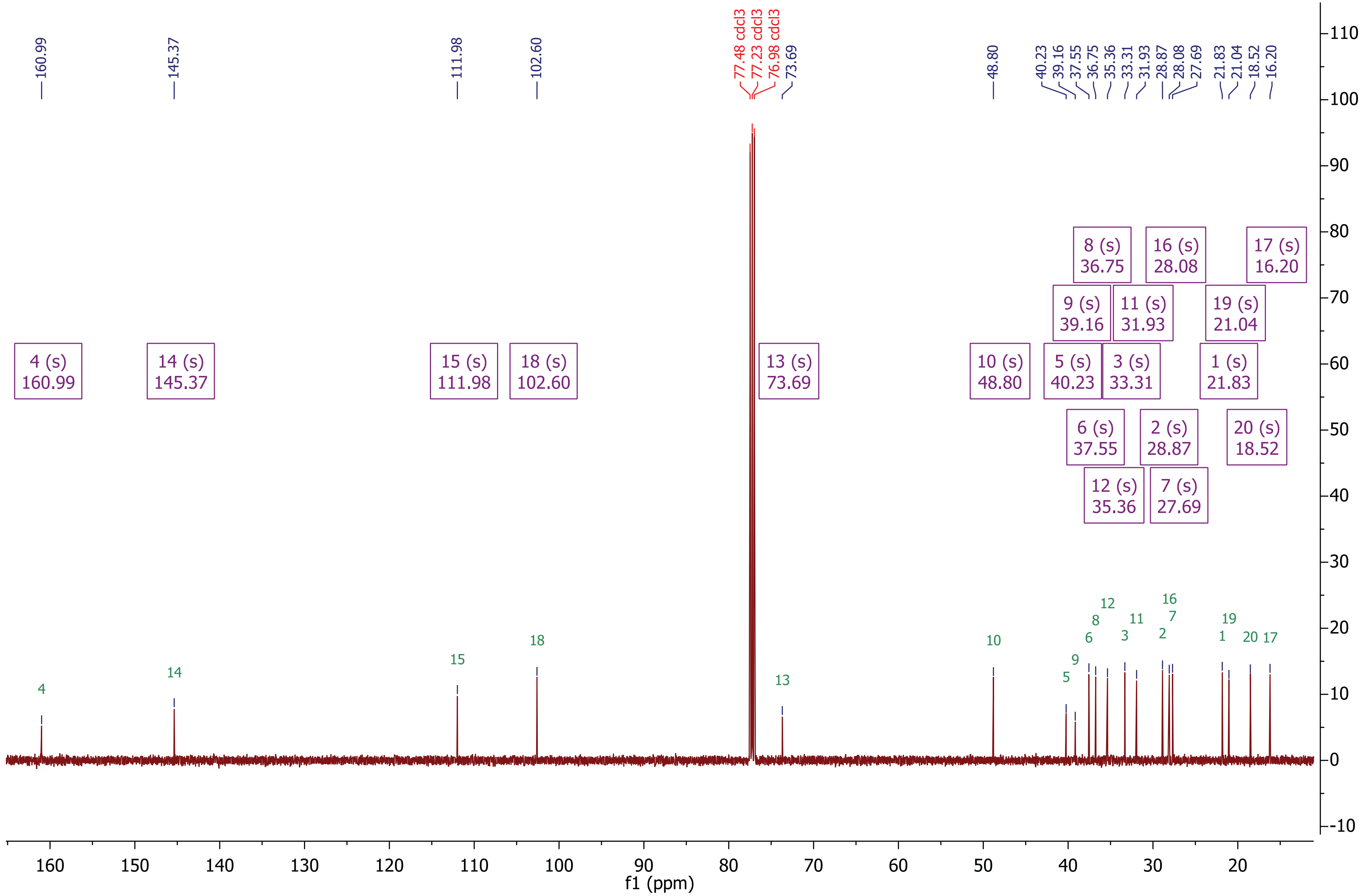

Figure S17-B. ^{13}C NMR of neo-cleroda-4(18),14-dien-13-ol [**37**].

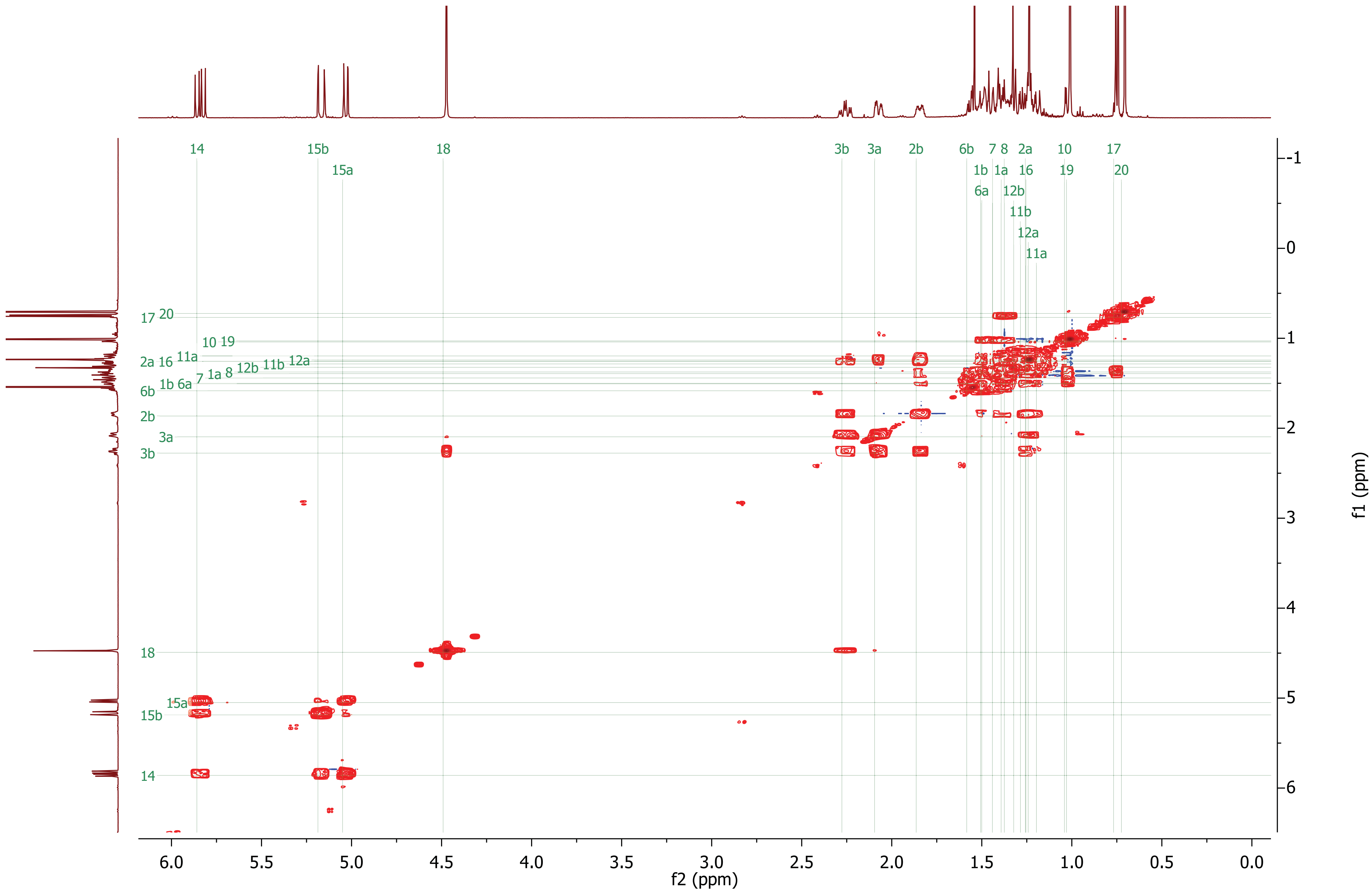

Figure S17-C. ^{1}H-^{1}H COSY of neo-cleroda-4(18),14-dien-13-ol [**37**].

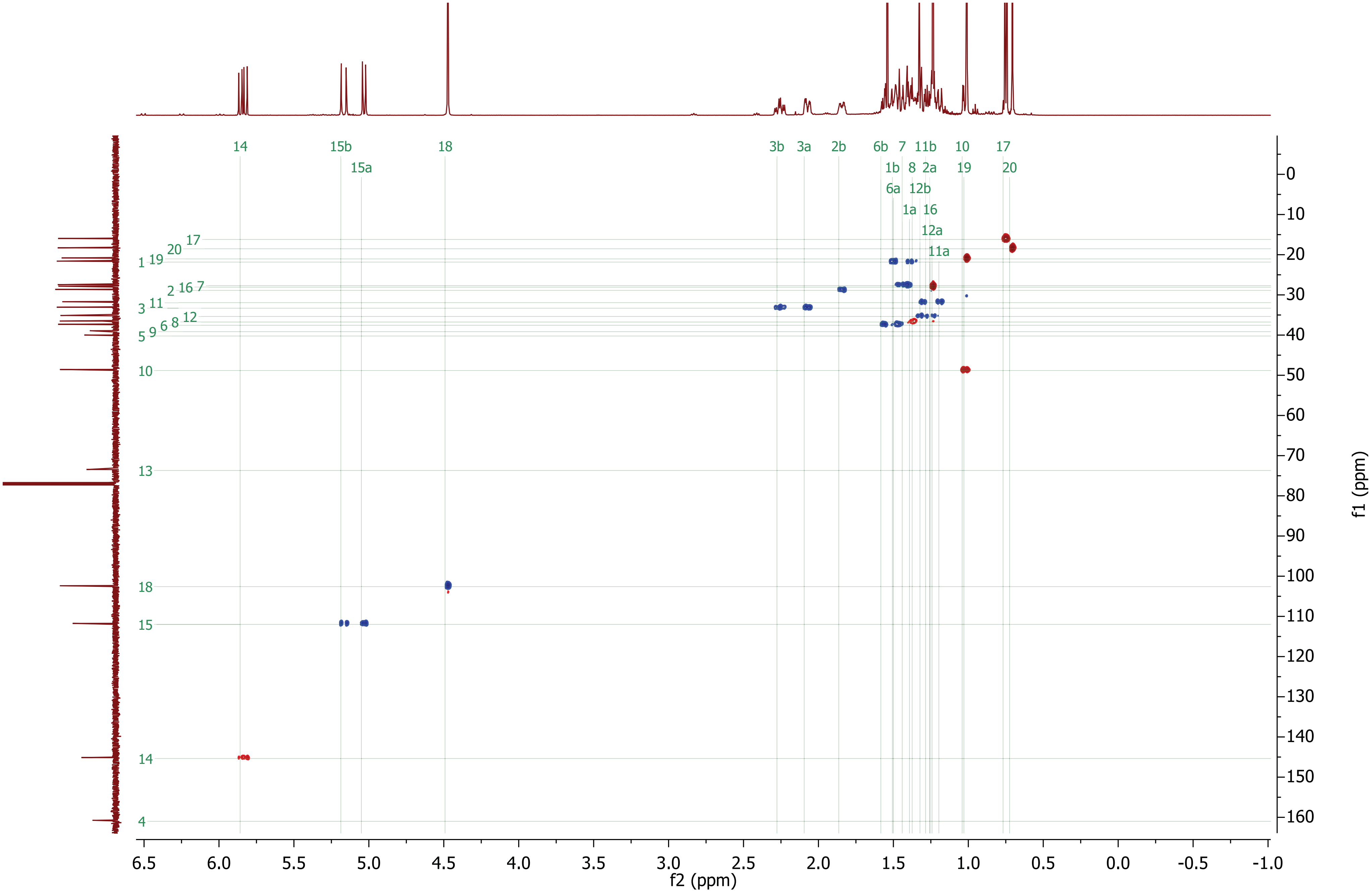

Figure S17-D. ^{1}H-^{13}C HSQC of neo-cleroda-4(18),14-dien-13-ol [**37**].

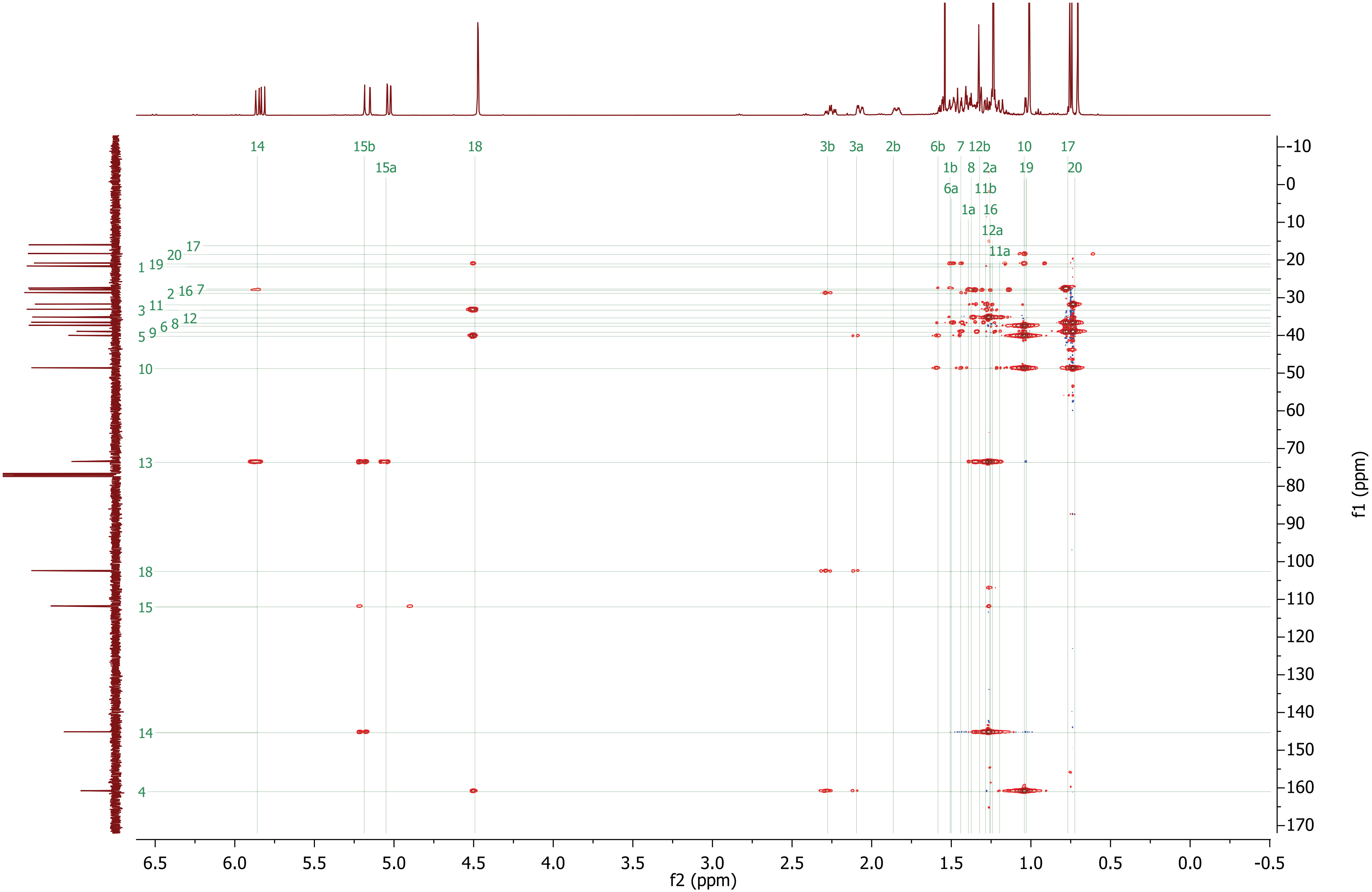

Figure S17-E. ^{1}H-^{13}C HMBC of neo-cleroda-4(18),14-dien-13-ol [**37**].

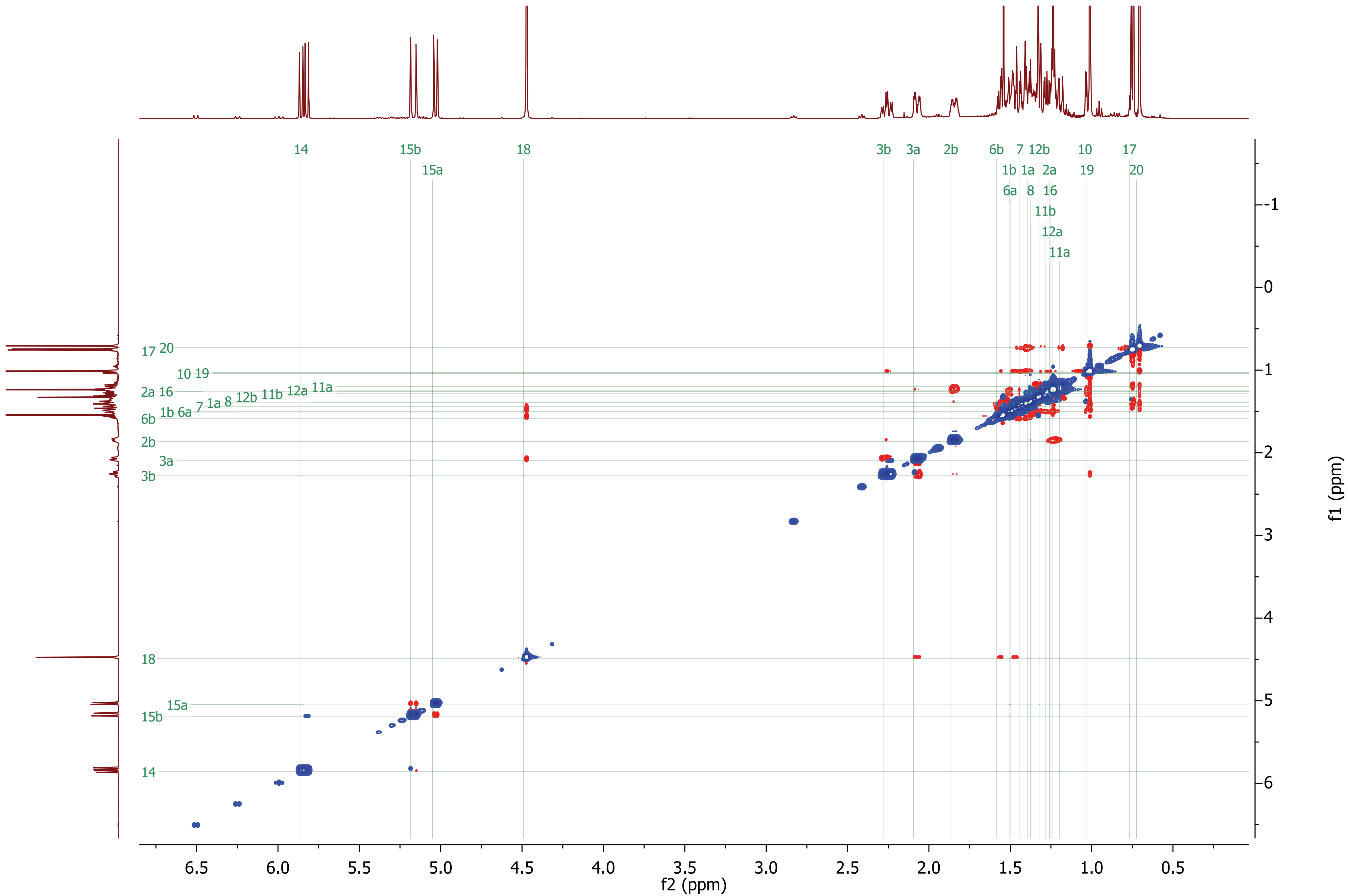

Figure S17-F. ^{1}H NOESY of neo-cleroda-4(18),14-dien-13-ol [**37**].

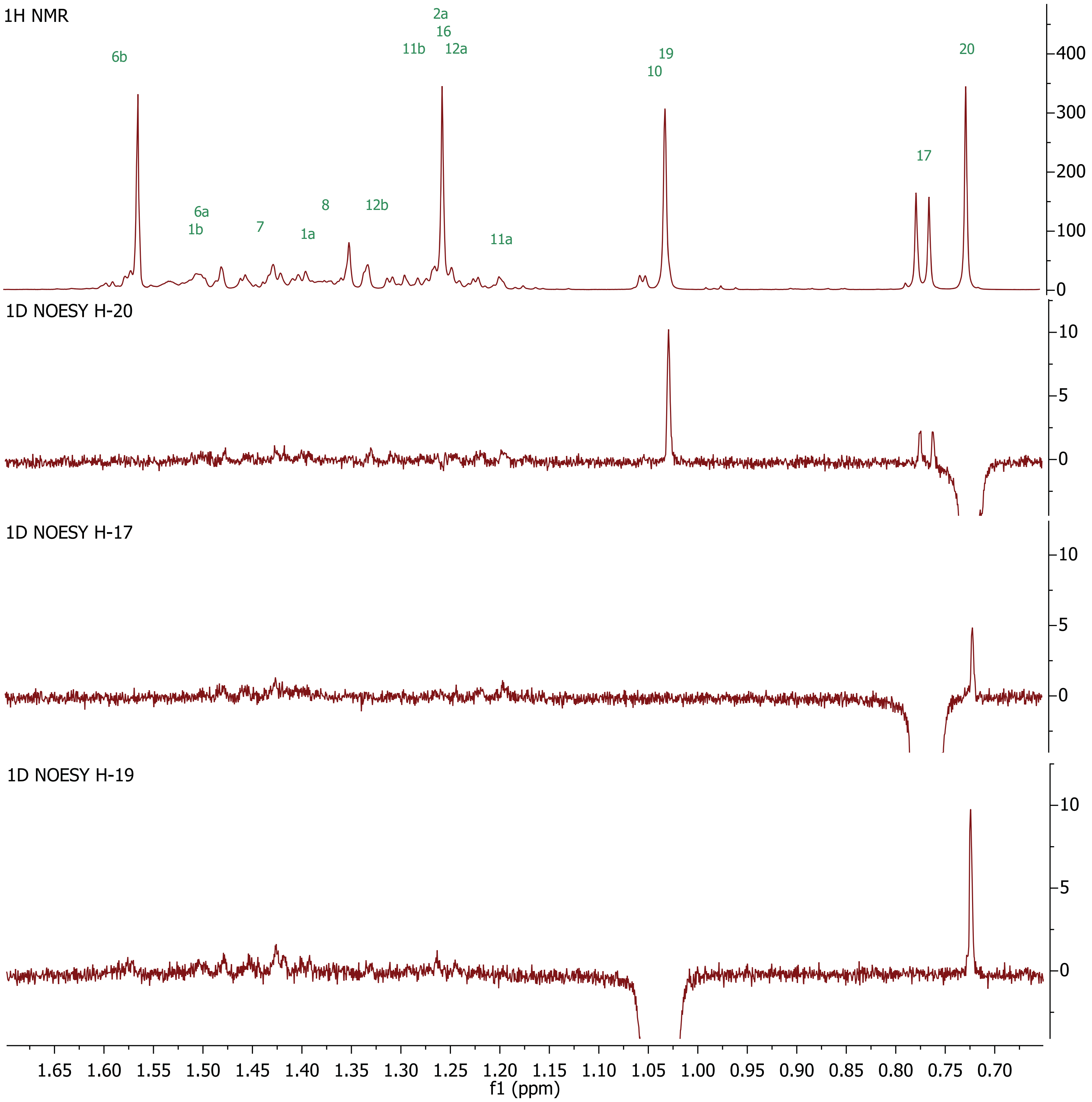

Figure S17-G. ^{1}H 1D-NOESY of neo-cleroda-4(18),14-dien-13-ol [**37**].

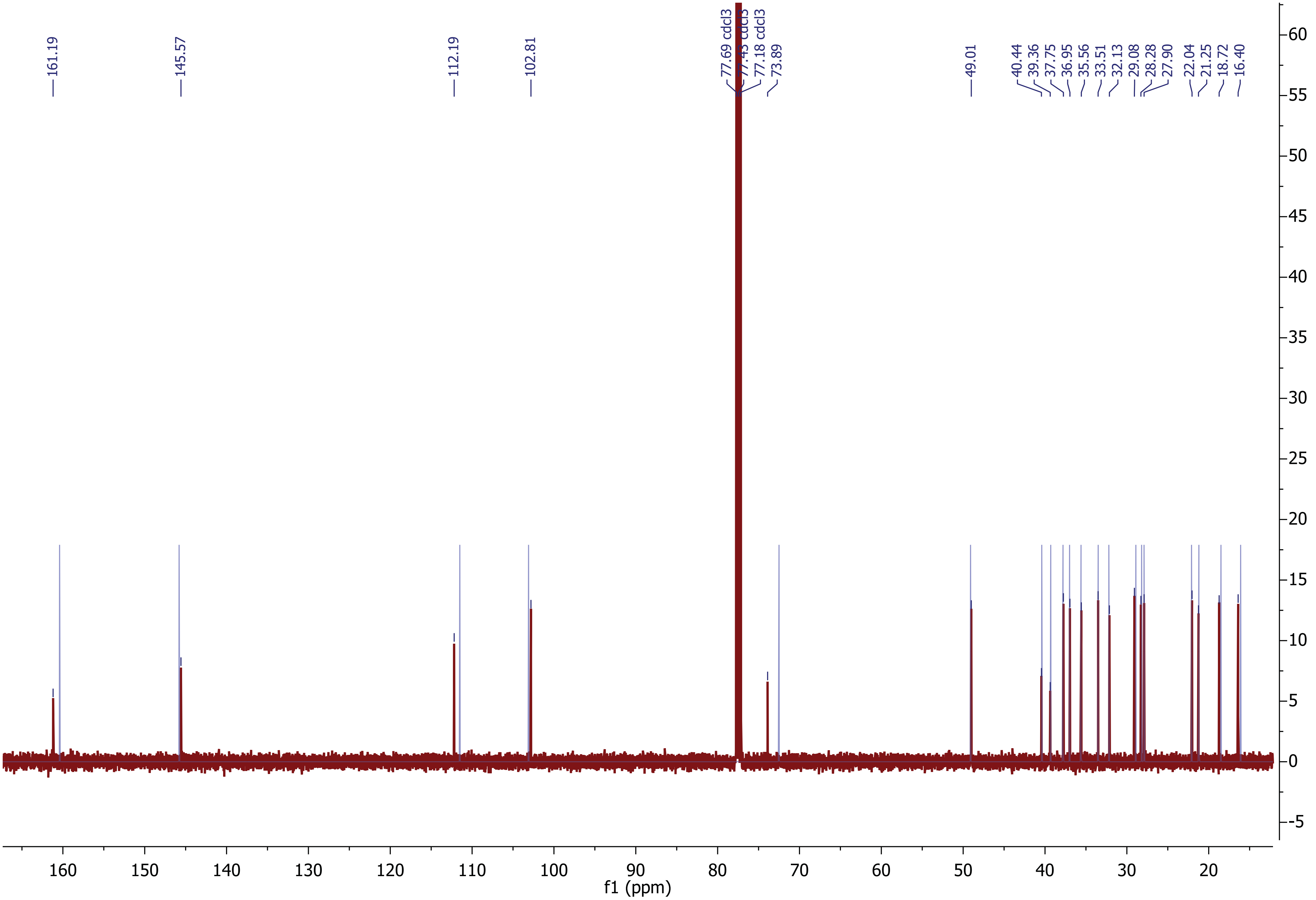

Figure S17-H. Overlay of ^{13}C NMR of neo-cleroda-4(18),14-dien-13-ol [**37**] (red) with ^{13}C NMR spectrum (blue) reconstructed from shifts reported for the same compound by Rudi and Kashman (1992) (DOI: 10.1021/np50088a004).

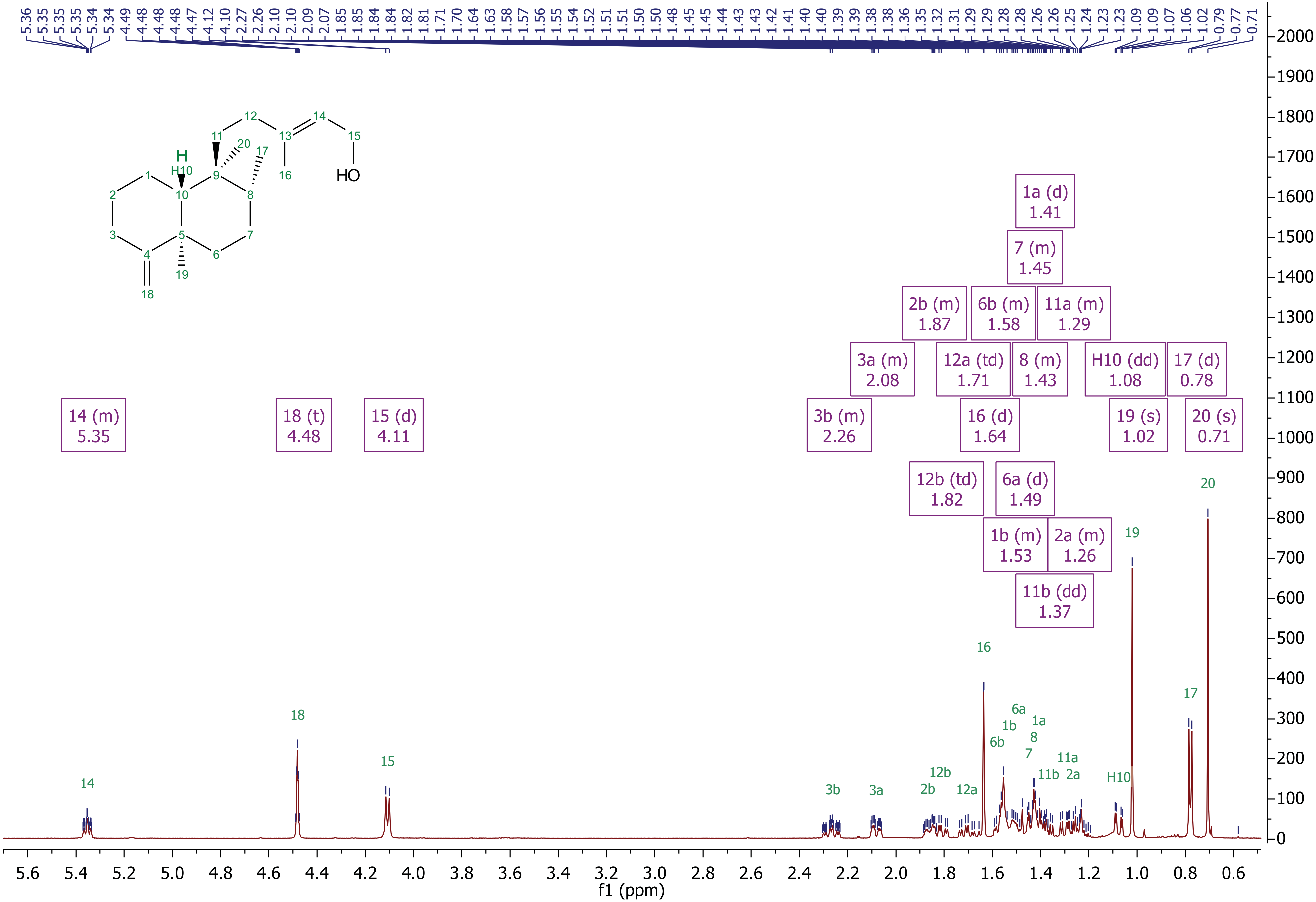

Figure S18-A. ^{1}H NMR of neo-cleroda-4(18),13E-diene-15-ol [**38a**].

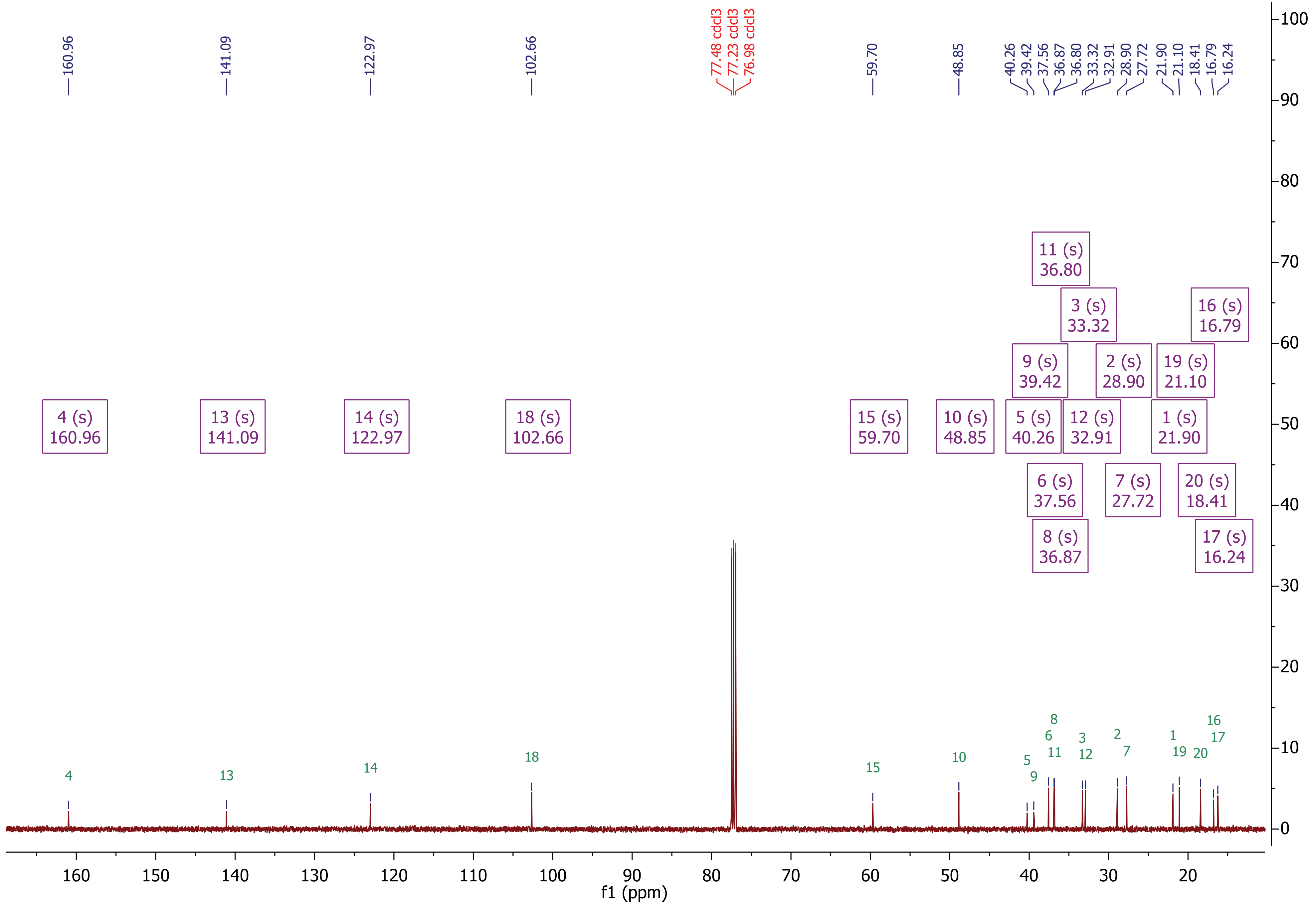

Figure S18-B. ^{13}C NMR of neo-cleroda-4(18),13E-diene-15-ol [**38a**].

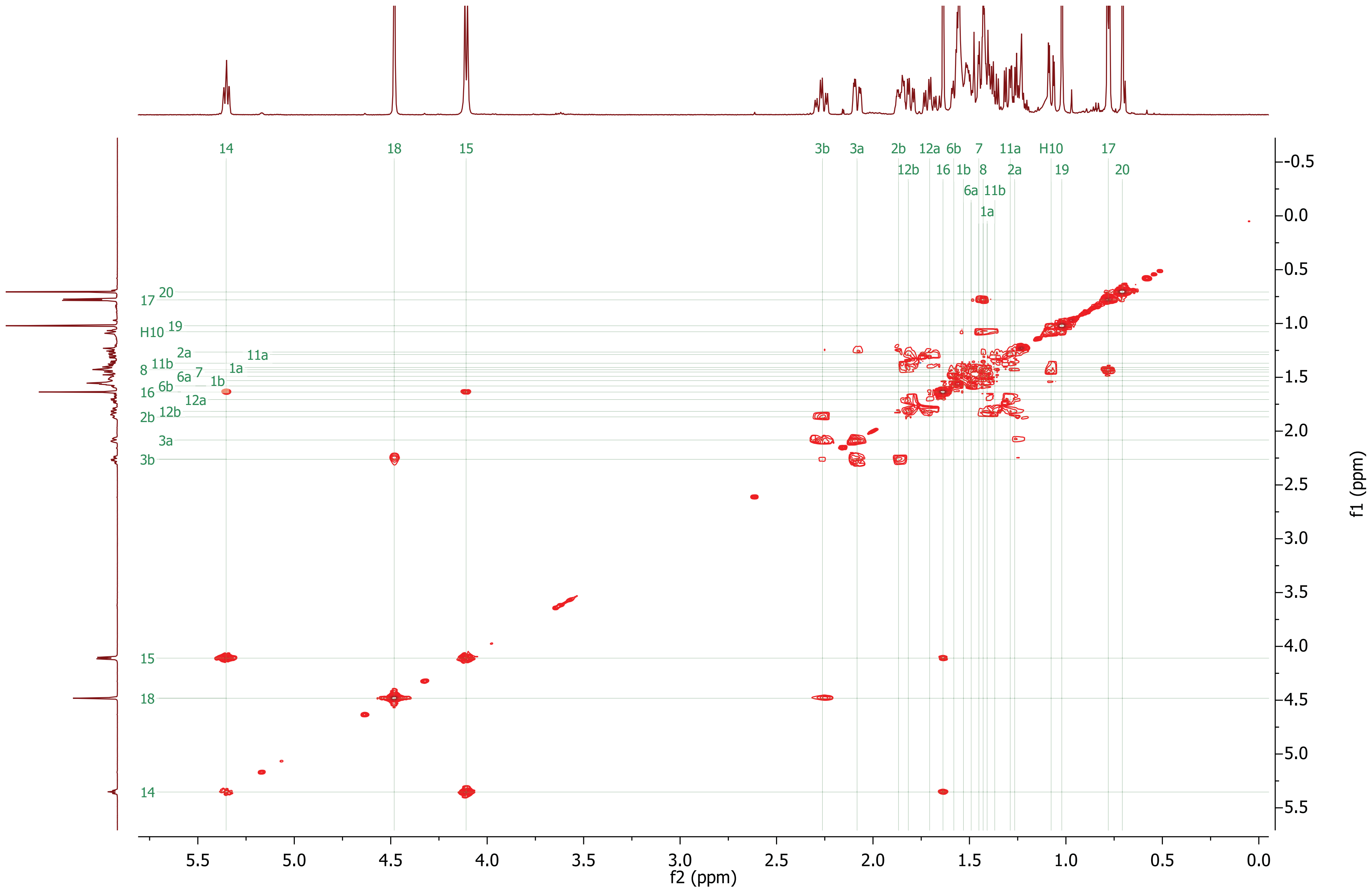

Figure S18-C. ^{1}H-^{1}H COSY of neo-cleroda-4(18),13E-diene-15-ol [**38a**].

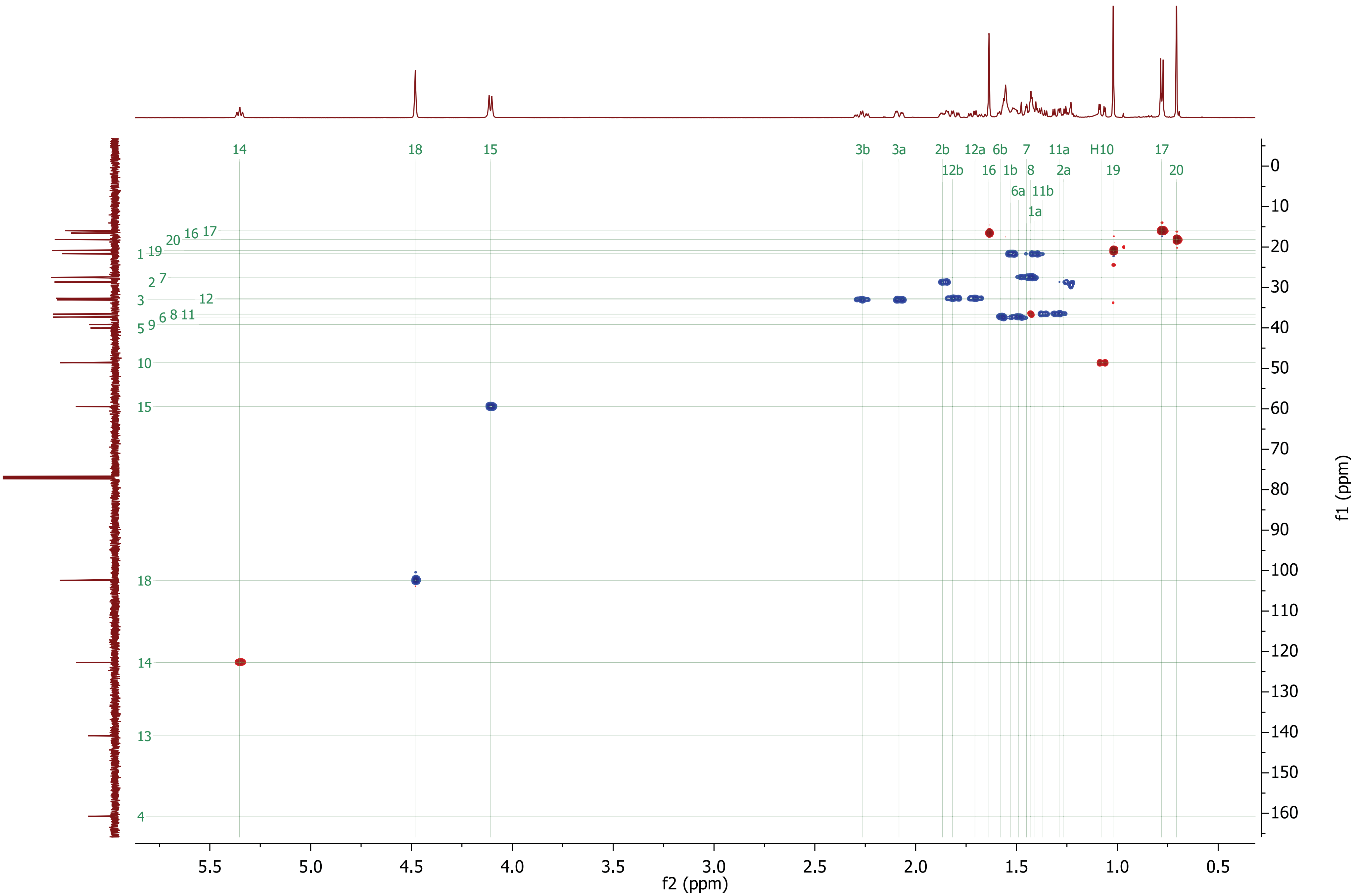

Figure S18-D. ^{1}H-^{13}C HSQC of neo-cleroda-4(18),13E-diene-15-ol [**38a**].

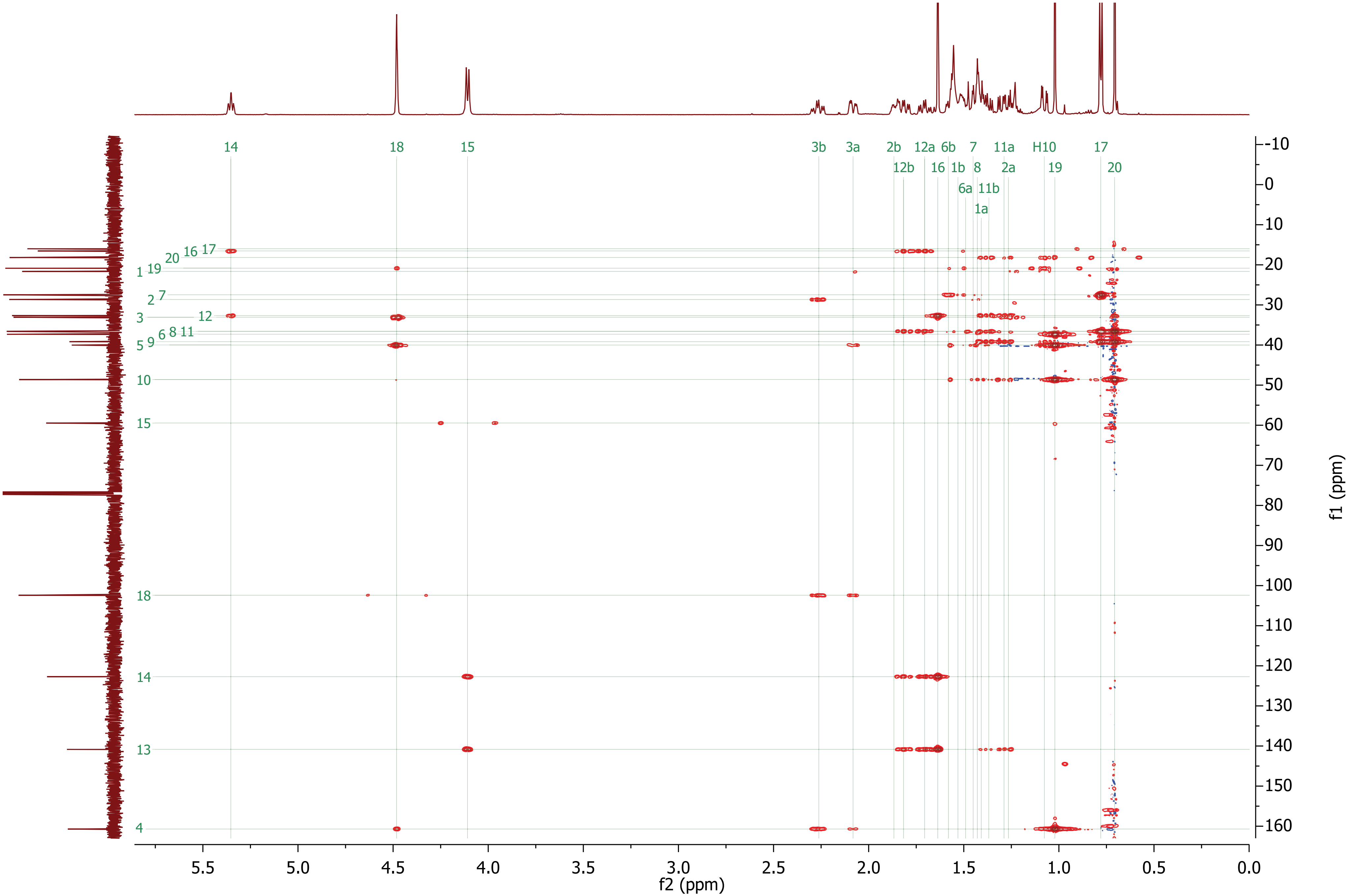

Figure S18-E. ^{1}H-^{13}C HMBC of neo-cleroda-4(18),13E-diene-15-ol [**38a**].

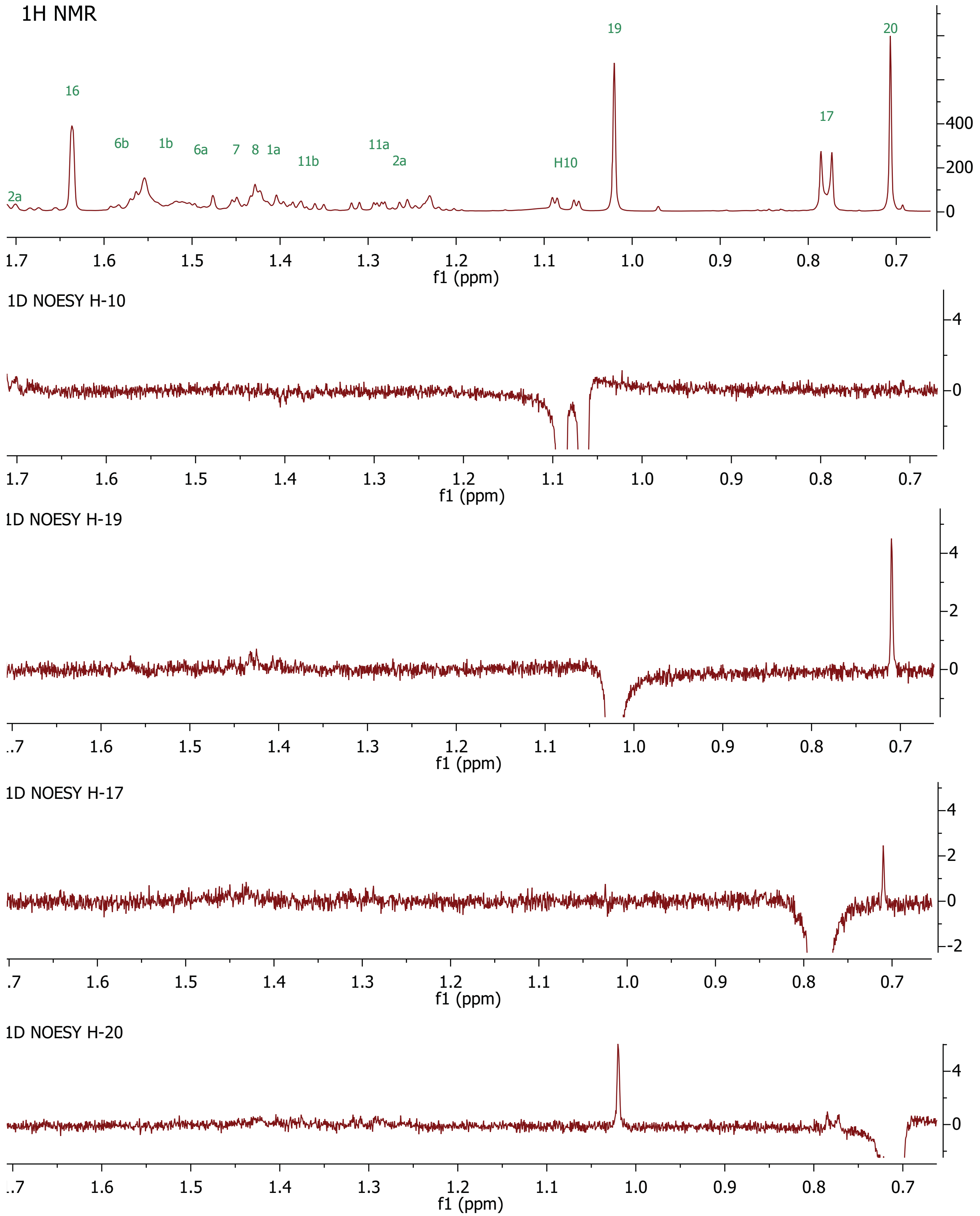

Figure S18-F. ^{1}H 1D-NOESY of neo-cleroda-4(18),13E-diene-15-ol [**38a**].

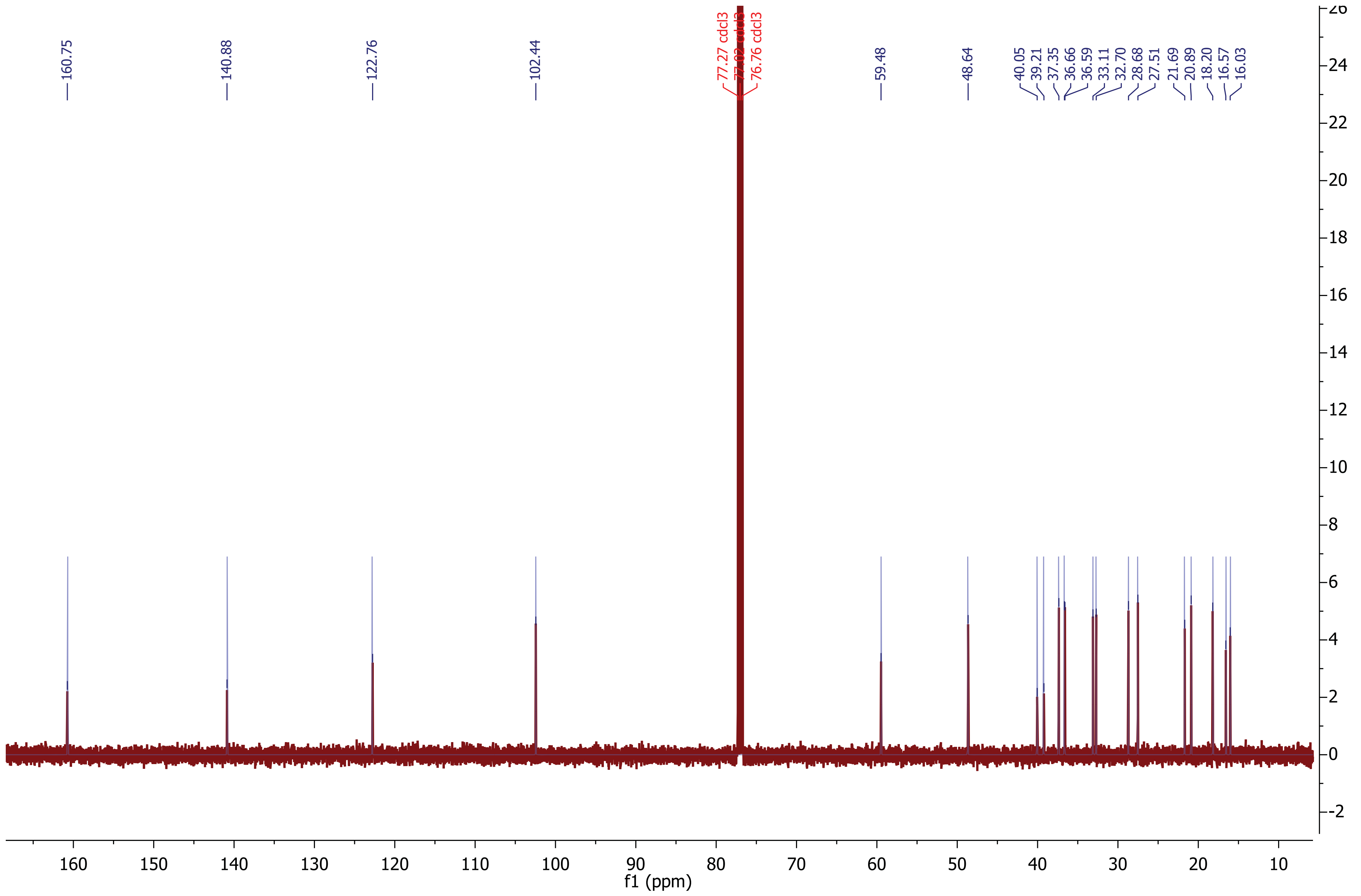

Figure S18-G. Overlay of ^{13}C NMR of neo-cleroda-4(18),13E-diene-15-ol [**38a**] (red) with ^{13}C NMR spectrum (blue) reconstructed from shifts reported for the same compound by Ohsaki (1994) (DOI: 10.1016/S0960-894X(01)80834-9).

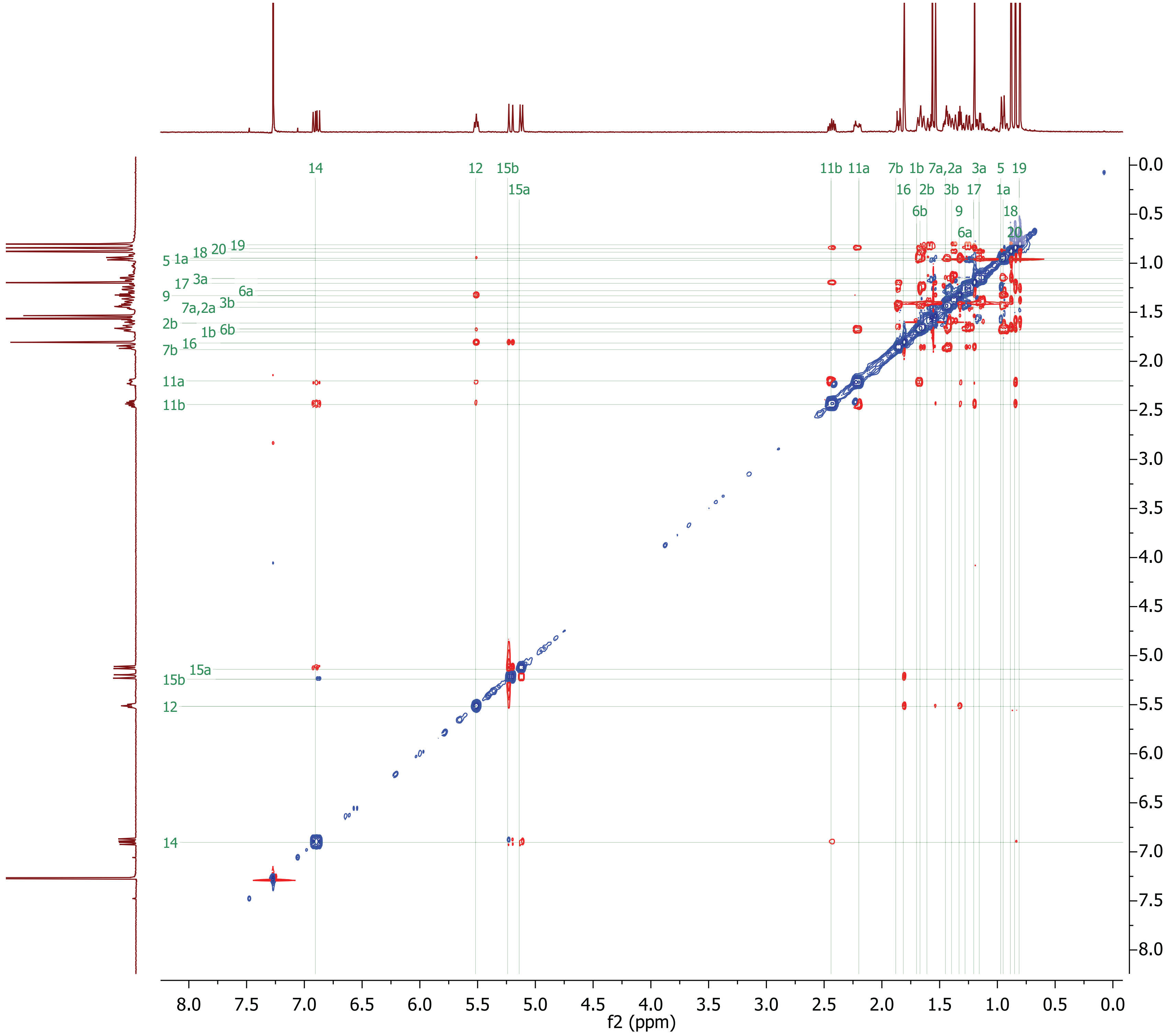

Figure S19. NOESY of (+)-cis-abienol.

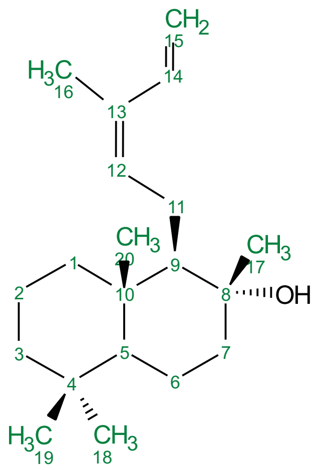

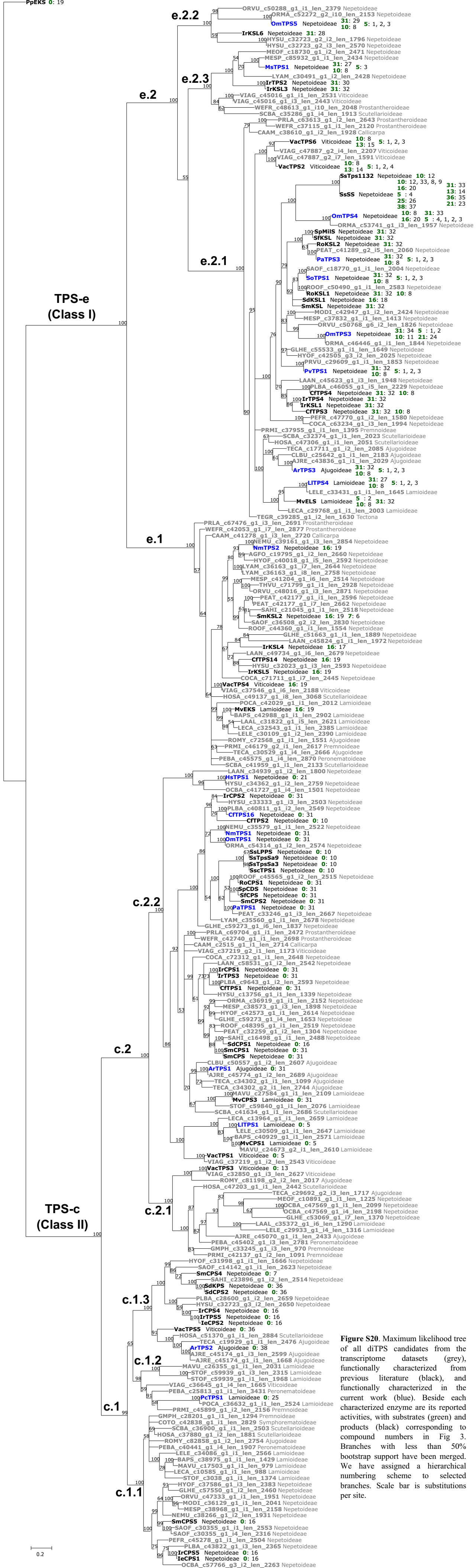

Figure S20. Maximum likelihood tree of all diTPS candidates from the transcriptome datasets (grey), functionally characterized from previous literature (black), and functionally characterized in the current work (blue). Beside each characterized enzyme are its reported activities, with substrates (green) and products (black) corresponding to compound numbers in Fig 3. Branches with less than 50% bootstrap support have been merged. We have assigned a hierarchical numbering scheme to selected branches. Scale bar is substitutions per site.

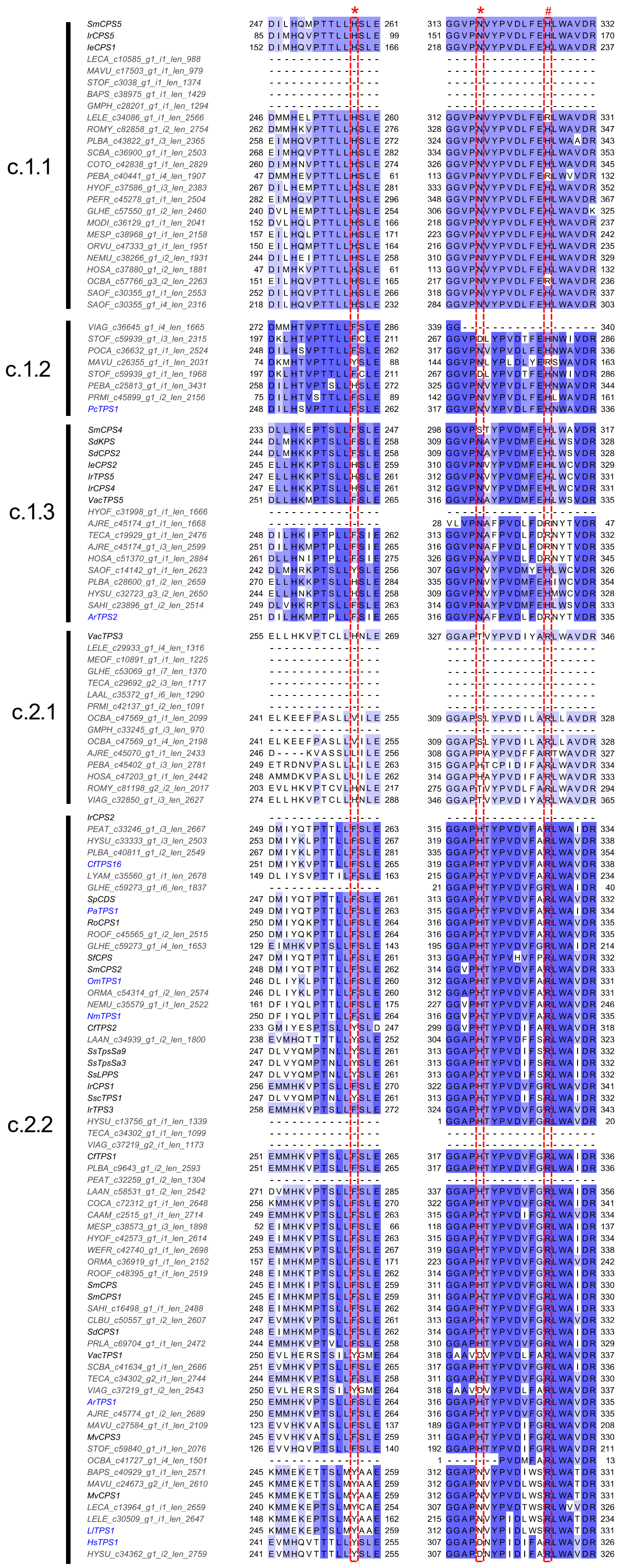

Figure S21: Activity-determining regions in an alignment of previously known (black), newly characterized (blue), and candidate (grey) TPS-c enzymes from Lamiaceae. Red stars indicate residues previously implicated in catalytic specificity. Histidine at the first position and asparagine at the second position have been associated with *ent*-CPP synthase activity. Red hash indicates residue previously implicated in Mg^{2+} driven inhibition. A histidine in this position leads to sensitivity to Mg^{2+} inhibition, which is characteristic of *ent*-CPP synthases involved in gibberellin biosynthesis, whereas enzymes of specialized metabolism lacking the histidine showed no susceptibility to Mg^{2+} inhibition. Positions are colored to indicate conservation within each subgroup.

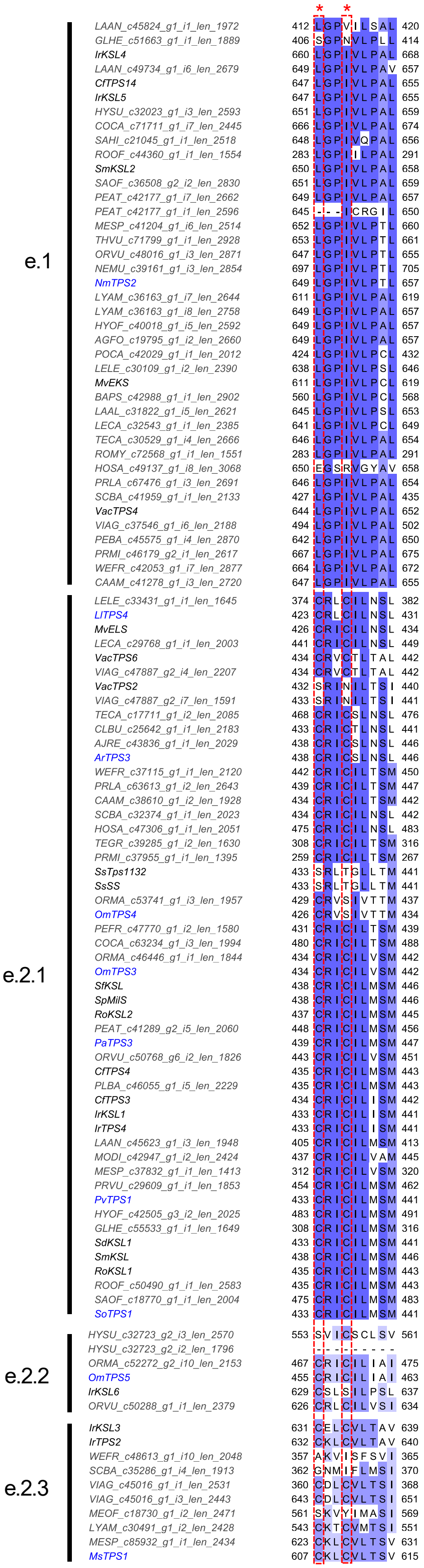

Figure S22: An activity-determining region in an alignment of previously known (black), newly characterized (blue), and candidate (grey) TPS-e enzymes from Lamiaceae. Red stars indicate residues previously implicated in catalytic specificity. The combination of leucine and isoleucine has been implicated in contributing to *ent*-kaurene synthase activity. Positions are colored to indicate conservation within each subgroup.
